# Nickel-Catalyzed Carbonylative Synthesis of β‑Ketonitriles from α‑Bromonitriles and Arylboronic Acids

**DOI:** 10.1021/acs.orglett.6c01685

**Published:** 2026-05-08

**Authors:** Jiacong Ma, Yuting Jiang, Zilin Huang, Xinxin Qi, Xiao-Feng Wu

**Affiliations:** † School of Chemistry and Chemical Engineering, Key Laboratory of Surface & Interface Science of Polymer Materials of Zhejiang Province, 12646 Zhejiang Sci-Tech University, Hangzhou, Zhejiang 310018, People’s Republic of China; ‡ Dalian National Laboratory for Clean Energy, Dalian Institute of Chemical Physics, Chinese Academy of Sciences, 116023 Dalian, Liaoning, China; ¶ 28392 Leibniz-Institut für Katalyse e.V., Albert-Einstein-Straße 29a, Rostock 18059, Germany

## Abstract

A new carbonylation reaction of α-bromonitriles with arylboronic acids has been explored for the synthesis of β-ketonitriles. Notably, the reaction is catalyzed by an inexpensive nickel catalyst and operates under mild reaction conditions without the direct use of gaseous CO. It demonstrates a general and complementary carbonylation route for the synthesis of valuable β-ketonitrile products, and a variety of products were obtained in moderate to high yields with good functional group tolerance. Moreover, the synthetic utility of this method is evidenced by a scale-up experiment and subsequent functionalization of the resulting β-ketonitriles.

## Graphical abstract

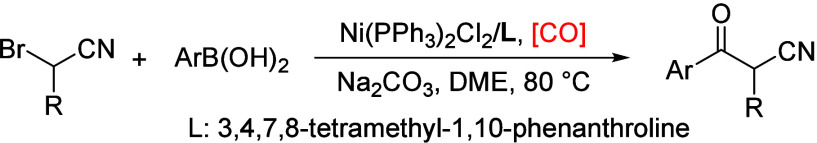

β-Ketonitriles are one of the most useful and essential structural moieties in organic and medicinal chemistry. With their impressive reactivities, they play an important role as key precursors for the synthesis of various heterocycles, such as pyrroles,^1^ furans,^2^ pyridines,^3^ thiophenes,^4^ thioazoles,^5^ etc. In addition, a wide range of biologically and pharmacologically active molecules can be prepared from them, for example, anti-inflammatory agents,^6^ antidepressants,^7^ NHE-1 inhibitors,^8^ and antimicrobials.^9^ Therefore, a series of synthetic strategies have been developed to access β-ketonitriles with conventional methods including acylation of alkyl nitriles with strong bases^10^ and cyanation of α-haloketones with cyanide anions.^11^ However, these classical methods usually suffer from some drawbacks, involving harsh reaction conditions, narrow substrate scope, and toxic reagents. In this regard, the development of novel and efficient methods for the preparation of β-ketonitriles is of great interest and highly desired.

During recent years, the palladium-catalyzed carbonylation reaction has proven to be a very effective method for the synthesis of β-ketonitriles. In 2012, Lee and co-workers reported the first carbonylative strategy toward β-ketonitriles preparation through palladium-catalyzed carbonylation reactions of aryl iodides with (trimethylsilyl)­acetonitrile (Scheme 1, eq a).^12a,12b^ Later, two similar palladium-catalyzed carbonylation-decarboxylation sequences were developed by the same group and Skrydstrup’s group.^12c,13^ Alternative protocols have also been disclosed by Beller and Skrydstrup’s group for the synthesis of α-substituted β-ketonitriles (Scheme 1, eqs b and c).^14,15^ Although these methods have provided practical and efficient routes to β-ketonitriles, they are still restricted to the use of toxic CO gas, organometallic reagents, and expensive transition-metal catalyst. Therefore, the exploration of a synthetic route that operates with alternative CO source and much lower-cost catalyst continues to be a key objective.

**Scheme 1:**
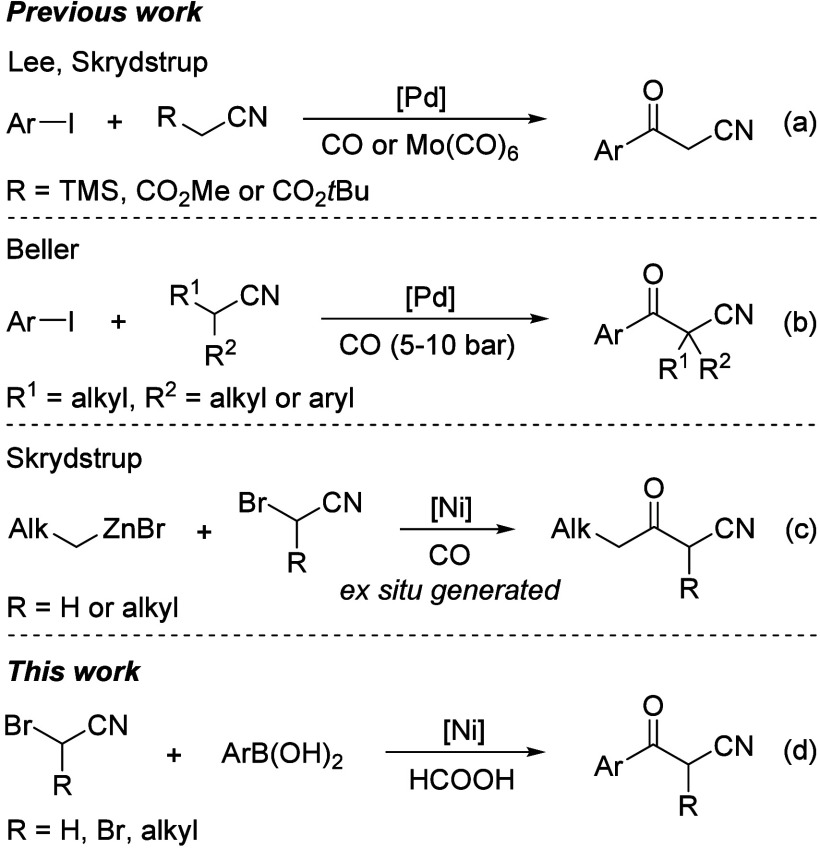
Carbonylative Synthesis of β-Ketonitriles

Nickel is a promising catalyst due to its natural abundance, low cost, and remarkable activity toward certain bonds. However, the application of nickel in carbonylation reactions is severely limited owing to its strong affinity with CO, which results in the formation of the volatile and highly toxic Ni­(CO)_4_.^16^ Such difficulties were observed, and the application of low-pressure CO gas or CO surrogate tend to be a good choice.^17^ Given our enduring interest in the nickel-catalyzed carbonylation reaction,^18^ herein, we wish to disclose a new nickel-catalyzed carbonylation reaction of α-bromonitriles with arylboronic acids for the synthesis of β-ketonitriles by using formic acid as the CO precursor, which is green and efficient in CO release (Scheme 1, eq d).

Initially, α-bromoacetonitrile **1a** and phenylboronic acid **2a** were selected as the model substrates to screen the reaction conditions. By applying Ni­(PPh_3_)_2_Cl_2_ as the catalyst, dtbbpy as the ligand, Na_2_CO_3_ as the base, and formic acid as the CO source in 1,4-dioxane at 80 °C for 20 h, the desired product **3aa** was obtained in 30% yield (Table 1, entry 1). Solvent screening showed that DME tends to be the best solvent (Table 1, entries 2–5), providing the target product **3aa** in 80% yield, which might benefit from its better coordinating ability with nickel precatalyst and then be ready for real active complex formation (Table 1, entry 4). Then, nickel catalysts, such as Ni­(acac)_2_, Ni­(OTf)_2_, NiCl_2_, NiCl_2_·DME, and Ni­(Py)_4_Cl_2_ were studied, and decreased yields were observed (Table 1, entries 6–10). Next, the effect of ligands was investigated; lower yields were formed with **L1**, **L2**, and **L4** (Table 1, entries 11–12, 14), whereas by using **L3** as the ligand, the final product **3aa** was obtained in 82% yield (Table 1, entry 13). Subsequently, K_2_CO_3_, NaHCO_3_, Et_3_N, and DIPEA were applied as the base, and no further improvement was achieved (Table 1, entries 15–18). Notably, upon heating or cooling the temperature to 100 or 60 °C, the yields dropped (Table 1, entries 19–20).

**Table 1:**
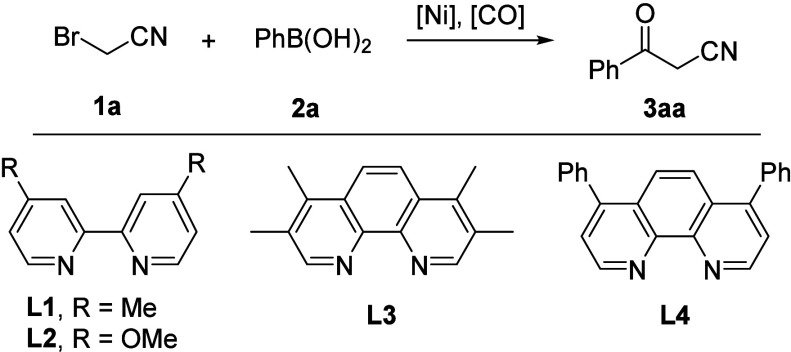
Screening of Reaction Conditions^a^

entry	catalyst	ligand	base	solvent	yield (%)
1	Ni(PPh_3_)_2_Cl_2_	dtbbpy	Na_2_CO_3_	1,4-dioxane	30
2	Ni(PPh_3_)_2_Cl_2_	dtbbpy	Na_2_CO_3_	THF	0
3	Ni(PPh_3_)_2_Cl_2_	dtbbpy	Na_2_CO_3_	CH_3_CN	0
4	Ni(PPh_3_)_2_Cl_2_	dtbbpy	Na_2_CO_3_	DME	80
5	Ni(PPh_3_)_2_Cl_2_	dtbbpy	Na_2_CO_3_	toluene	0
6	Ni(acac)_2_	dtbbpy	Na_2_CO_3_	DME	0
7	Ni(OTf)_2_	dtbbpy	Na_2_CO_3_	DME	65
8	NiCl_2_	dtbbpy	Na_2_CO_3_	DME	0
9	NiCl_2_·DME	dtbbpy	Na_2_CO_3_	DME	70
10	Ni(Py)_4_Cl_2_	dtbbpy	Na_2_CO_3_	DME	15
11	Ni(PPh_3_)_2_Cl_2_	**L1**	Na_2_CO_3_	DME	72
12	Ni(PPh_3_)_2_Cl_2_	**L2**	Na_2_CO_3_	DME	64
13	Ni(PPh_3_)_2_Cl_2_	**L3**	Na_2_CO_3_	DME	82
14	Ni(PPh_3_)_2_Cl_2_	**L4**	Na_2_CO_3_	DME	57
15	Ni(PPh_3_)_2_Cl_2_	**L3**	K_2_CO_3_	DME	trace
16	Ni(PPh_3_)_2_Cl_2_	**L3**	NaHCO_3_	DME	31
17	Ni(PPh_3_)_2_Cl_2_	**L3**	Et_3_N	DME	trace
18	Ni(PPh_3_)_2_Cl_2_	**L3**	DIPEA	DME	0
19^b^	Ni(PPh_3_)_2_Cl_2_	**L3**	Na_2_CO_3_	DME	68
20^c^	Ni(PPh_3_)_2_Cl_2_	**L3**	Na_2_CO_3_	DME	25

a Reaction conditions: α-bromoacetonitrile **1a** (0.2 mmol), phenylboronic acid **2a** (0.3 mmol), catalyst (10 mol %), ligand (10 mol %), [CO] (HCOOH + Ac_2_O, 2.0 mmol, 1:1 ratio), base (2.0 equiv), solvent (2.0 mL), 80 °C, 20 h, isolated yields. DME: dimethoxyethane.

b 100 °C.

c 60 °C.

With the optimal reaction conditions in hand, we examined the substrate scope of this nickel-catalyzed carbonylation reaction. As shown in Scheme 2, a variety of arylboronic acids were applied, and electron-rich groups such as methyl, *tert*-butyl, methoxy, trifluoromethoxy, and *N*,*N*-diphenyl were well tolerated to afford the desired products in moderate to high yields (**3ab**–**3ah**). Aryl boronic acids with halogen substituents, such as fluoro and chloro, proved to be compatible and led to the target products in good yields (**3ai**, **3aj**). Subsequently, biphenyl, naphthalen-2-yl, and 9,9-dimethyl-9*H*-fluoren-2-yl groups were investigated, and the corresponding products were formed in good to excellent yields (**3ak**–**3am**). Moreover, heteroaryl groups, including thiophen-3-yl and 9-phenyl-9*H*-carbazol-2-yl, proceeded smoothly, affording the expected products in 71% and 70% yields (**3an**, **3ao**). However, no desired product was detected when pyridine derivative boronic acids were used. Additionally, alkylboronic acid and alkenylboronic acid were also tested but without any success.

**Scheme 2:**
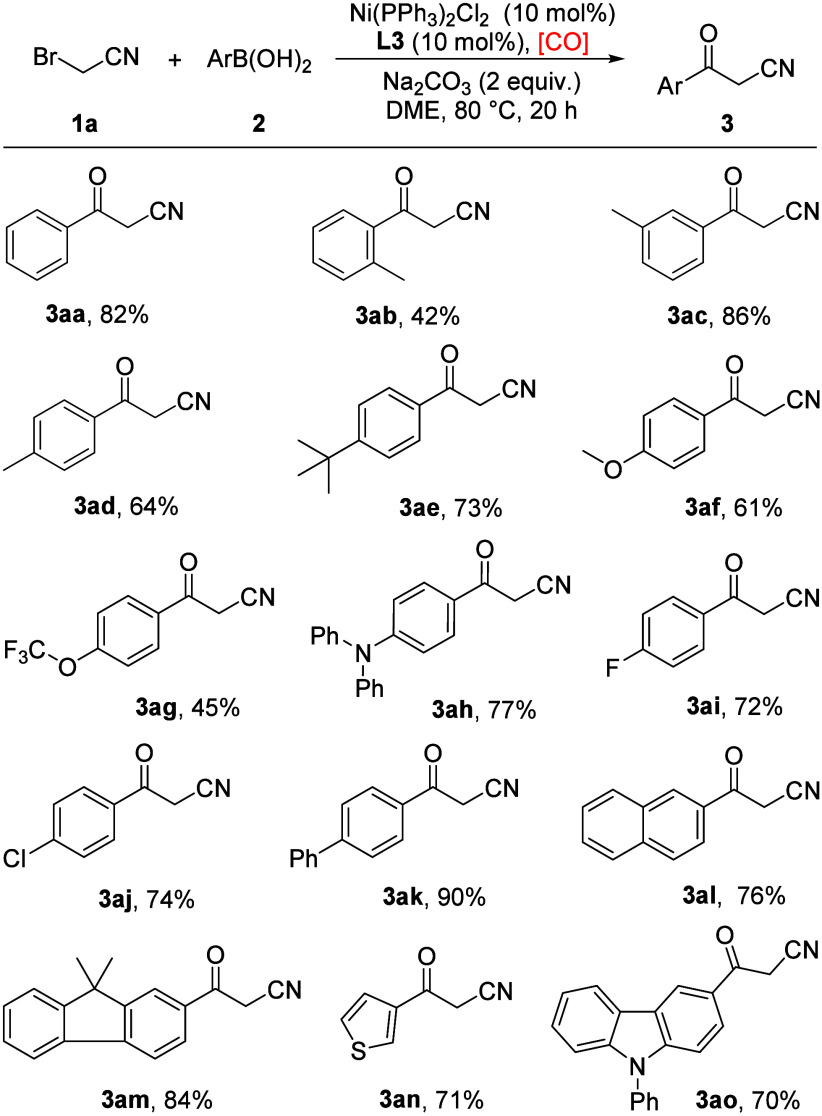
Substrate Scope of Arylboronic acids^a^

a Reaction conditions: α-bromoacetonitrile **1a** (0.2 mmol), arylboronic acids **2** (0.3 mmol), Ni­(PPh_3_)_2_Cl_2_ (10 mol %), **L3** (10 mol %), [CO] (HCOOH + Ac_2_O, 2.0 mmol, 1:1 ratio), Na_2_CO_3_ (2.0 equiv), DME (2.0 mL), 80 °C, 20 h. Isolated yield.

Next, we turned our attention to study the substrate scope of α-bromonitriles, and the results are summarized in Scheme 3. 2,2-Dibromoacetonitrile was first carried out with phenylboronic acid **2a** under standard reaction conditions; the desired product was obtained in 20% yield (**3ba**) together with a significant amount of **3aa** as the reductive debromination side product. Various unfunctionalized aliphatic α-bromonitriles were then studied, resulting in the expected products in excellent yields (**3ca**–**3fa**). Next, α-bromoacetonitriles containing cycloalkyl groups, such as cyclobutyl, cyclopentyl, and cyclohexyl, were well tolerated to give the corresponding products in good to excellent yields (**3ga**–**3ia**). Furthermore, substrates containing internal alkene and phenyl moieties also proved to be compatible, affording the target products in 86% and 75% yields (**3ja**, **3ka**). It is worth mentioning that no desired reaction occurred when α-chloroacetonitrile was tested as the substrate.

**Scheme 3:**
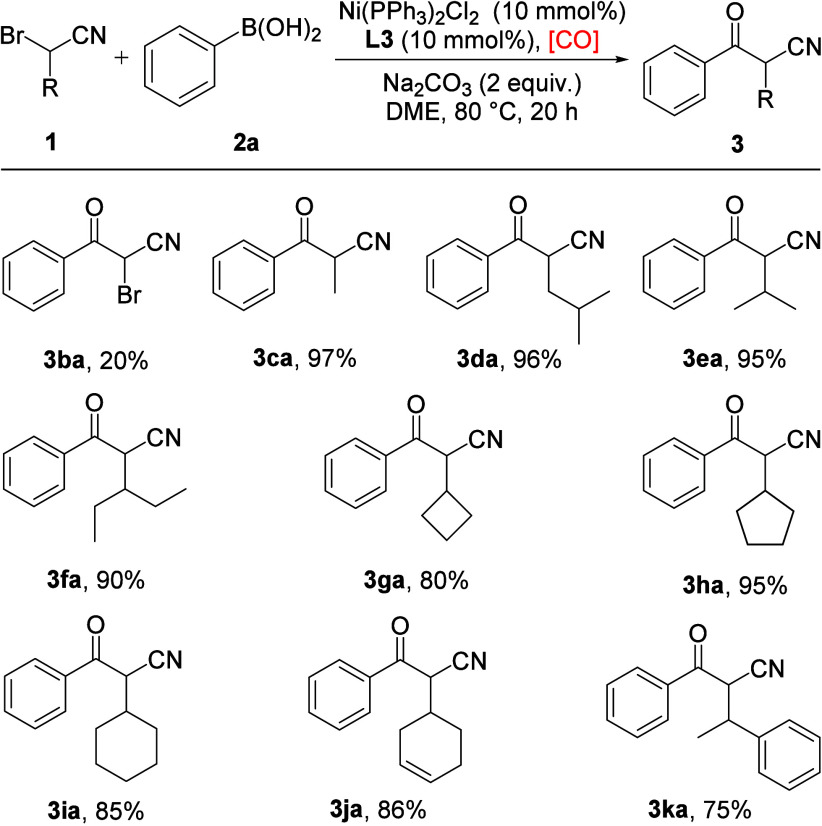
Substrate Scope of α-Bromonitriles^a^

a Reaction conditions: α-bromonitriles **1** (0.2 mmol), phenylboronic acid **2a** (0.3 mmol), Ni­(PPh_3_)_2_Cl_2_ (10 mol %), **L3** (10 mol %), [CO] (HCOOH + Ac_2_O, 2.0 mmol, 1:1 ratio), Na_2_CO_3_ (2.0 equiv), DME (2.0 mL), 80 °C, 20 h. Isolated yield.

Moreover, a control experiment was performed to gain more insight of the reaction mechanism (Scheme 4, eq a). TEMPO or DPE (1,1-diphenylethene) was added to the reaction mixture as a radical scavenger under the standard reaction conditions; the expected product **3aa** was not observed, which indicated that a free radical process may be included in this reaction. To further illustrate the synthetic utility of the developed strategy, a 1 mmol scale reaction was performed. The final product was isolated in 70% yield, even with a lower loading of Ni­(PPh_3_)_2_Cl_2_ (Scheme 4, eq b). Additionally, several chemical transformations of the β-ketonitrile products were conducted. The β-ketonitrile molecule **3aa** could react with hydroxylammonium hydrochloride and phenylhydrazine hydrochloride, respectively, and the corresponding isoxazole **4** and pyrazole **5** products were produced in 53% and 56% yields (Scheme 4, eqs c and d).

**Scheme 4:**
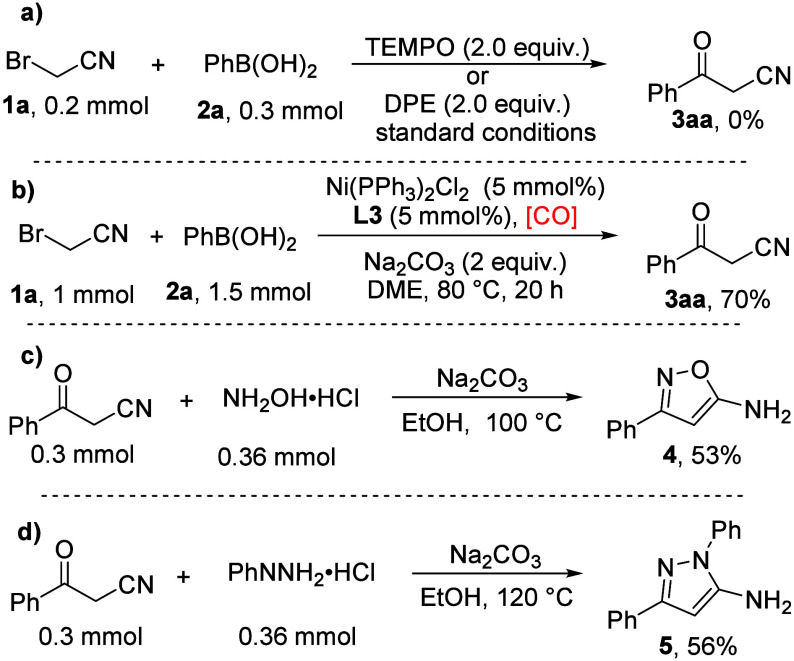
Control Experiment and Synthetic Applications

Based on above experiments and results, a plausible reaction mechanism is shown in Scheme 5. First, Ni­(I) undergoes transmetalation with arylboronic acids **2** to provide aryl Ni­(I) species **I**, which then engages in a single electron transfer (SET) process with α-bromonitriles **1**, generating radicals **A** and aryl Ni­(II) complexes **II**. Next, a CO coordination and insertion to aryl Ni­(II) complexes **II** occurs and leads to acyl Ni­(II) intermediates **III**, followed by a combination with radicals **A** to give acyl Ni­(III) intermediates **IV**. Finally, reductive elimination of intermediates **IV** affords the desired products **3** and regenerates Ni­(I) for the next catalytic cycle.

**Scheme 5:**
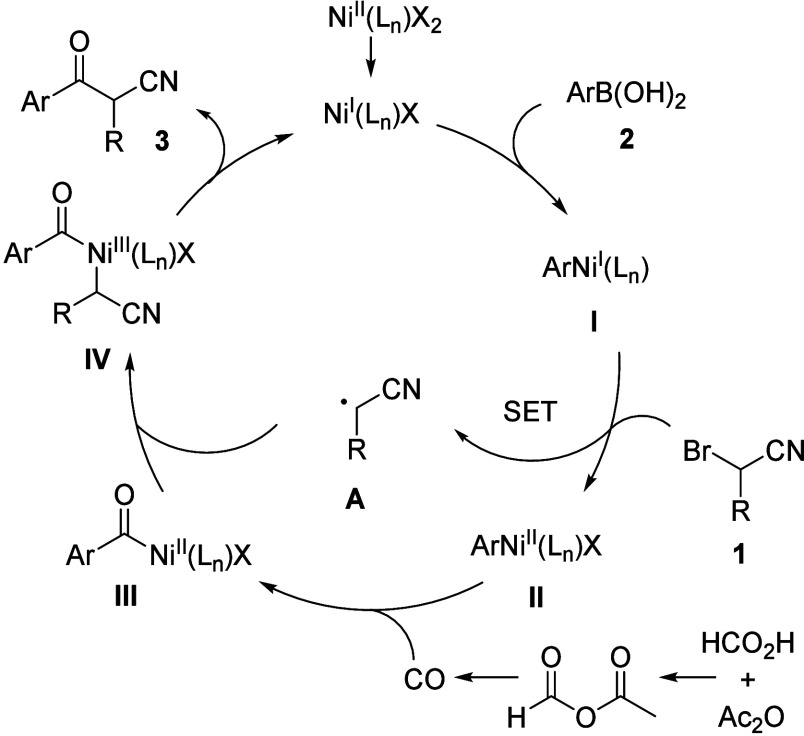
Proposed Reaction Mechanism

In summary, a general and effective strategy has been developed for the synthesis of β-ketonitriles via a nickel-catalyzed carbonylation reaction of α-bromonitriles with arylboronic acids. By using formic acid as the CO precursor, a variety of β-ketonitriles were obtained in moderate to high yields with good functional group tolerance. The approach employs an inexpensive nickel catalyst and avoids the use of toxic CO gas under mild conditions, thereby offering an efficient and practical strategy to access β-ketonitriles via a carbonylative process. Moreover, the synthetic value of this approach is highlighted by a gram-scale reaction and further transformations of the obtained β-ketonitrile products.

## Supplementary Material



## Data Availability

The data underlying this study are available in the published article and its Supporting Information.

## Notes

Experimental details, characterization data, and NMR spectra (PDF)

## References

[1] Chan C.-K., Chan Y.-L., Tsai Y.-L., Chang M.-Y. (2016). One-Pot Synthesis
of 2-Cyano-1,4-diketones: Applications to Synthesis of Cyanosubstituted
Furans, Pyrroles, and Dihydropyridazines. J.
Org. Chem..

[2] Hu J., Wei Y., Tong X. (2011). Phosphine-Catalyzed
(3 + 2) Annulations of γ-Functionalized Butynoates and 1*C*,3*O*-Bisnucleophiles: Highly Selective
Reagent-Controlled Pathways to Polysubstituted Furans. Org. Lett..

[3] Gao G., Wang P., Liu P., Zhang W., Mo L., Zhang Z. (2018). Deep eutectic solvent
catalyzed one-pot synthesis of
4,7-dihydro-1*H*-pyrazolo­[3,4-*b*]­pyridine-5-carbonitriles. Chin. J. Org. Chem..

[4] Puterová Z., Andicsová A., Végh D. (2008). Synthesis of π-conjugated thiophenes starting
from substituted 3-oxopropanenitriles via Gewald reaction. Tetrahedron.

[5] Sun J., Ge H., Zhen X., An X., Zhang G., Zhang-Negrerie D., Du Y., Zhao K. (2018). TBHP/AIBN-mediated synthesis of 2-amino-thioazoles
from active methylene ketones and thiourea under metal-free conditions. Tetrahedron.

[6] Ross J. R., Sowell J. W. (1987). Synthesis of a series of pyrrole-1-acetic
acids as
potential antiinflammatory agents. J. Heterocycl.
Chem..

[7] Wang G., Liu X., Zhao G. (2005). Polymer-supported chiral sulfonamide catalyzed one-pot
reduction of β-keto nitriles: a practical synthesis of (R)-fluoxetine
and (R)-duloxetine. Tetrahedron: Asymmetry.

[8] Lee S., Kim T., Lee B. H., Yoo S., Lee K., Yi K. Y. (2007). 3-Substituted-(5-arylfuran-2-ylcarbonyl)­guanidines
as NHE-1 inhibitors. Bioorg. Med. Chem. Lett..

[9] Loğoğlu E., Yilmaz M., Katircioğlu H., Yakut M., Mercan S. (2010). Synthesis
and biological activity studies of furan derivatives. Med. Chem. Res..

[10] Dorsch J. B., McElvain S. M. (1932). The Preparation
of Benzoylacetic Ester and Some of Its Homologs. J. Am. Chem. Soc..

[11] Kamila S., Zhu D., Biehl E. R., Hua L. (2006). Unexpected Stereorecognition in Nitrilase-Catalyzed Hydrolysis of *β*-Hydroxy Nitriles. Org. Lett..

[12] Park A., Lee S. (2012). Synthesis of Benzoylacetonitriles
from Pd-Catalyzed Carbonylation of Aryl Iodides and Trimethylsilylacetonitrile. Org. Lett..

[13] Jensen M. T., Juhl M., Nielsen D. U., Jacobsen M. F., Lindhardt A. T., Skrydstrup T. (2016). Palladium-Catalyzed
Carbonylative *α*-Arylation of *tert*-Butyl Cyanoacetate with (Hetero)­aryl
Bromides. J. Org. Chem..

[14] Schranck J., Burhardt M., Bornschein C., Neumann H., Skrydstrup T., Beller M. (2014). Palladium-Catalyzed
Carbonylative *α*-Arylation to *β*-Ketonitriles. Chem.—Eur. J..

[15] Donslund A. S., Neumann K. T., Corneliussen N. P., Grove E. K., Herbstritt D., Daasbjerg K., Skrydstrup T. (2019). Access to *β*-Ketonitriles through
Nickel-Catalyzed Carbonylative Coupling of *α*-Bromonitriles with Alkylzinc Reagents. Chem.—Eur.
J..

[16] Li L.-J., He Y., Yang Y., Guo J., Lu Z., Wang C., Zhu S., Zhu S.-F. (2024). Recent
Advances in Mn, Fe, Co, and Ni-Catalyzed Organic Reactions. CCS Chem..

[17] n Zhang, Y. ; Zhao, N. ; Lv, B. ; Chen, Y. Recent advances in ligand-accelerated nickel-catalyzed carbonylation under CO gas. Green Synth. Catal. 2025, in press; 10.1016/j.gresc.2025.12.009.

[18] Ma H., Hou C.-Y., Zhao R., Qi X., Wu X.-F. (2025). Nickel-Catalyzed Cyclization/Carbonylation Reaction
of *N*-Allylbromoacetamides with Arylboronic Acids
toward 2-Pyrrolidinones. Org. Lett..

