# Supplementary material for: Nickel-Catalyzed Carbonylative Synthesis of β‑Ketonitriles from α‑Bromonitriles and Arylboronic Acids
Source: Org Lett. 2026 May 8;28(20):6473–7. doi: 10.1021/acs.orglett.6c01685 (PMC13200237; doi:10.1021/acs.orglett.6c01685)

Supporting Information

Nickel-Catalyzed Carbonylative Synthesis of *β*-Ketonitriles from *α*-Bromonitriles and Arylboronic Acids

Jiacong Ma,† Yuting Jiang,† Zilin Huang,† Xinxin Qi,*,† Xiao-Feng Wu*,‡

† School of Chemistry and Chemical Engineering, Key Laboratory of Surface & Interface Science of Polymer Materials of Zhejiang Province, Zhejiang Sci-Tech University, Hangzhou, Zhejiang 310018, People's Republic of China

E-mail: xinxinqi@zstu.edu.cn

‡ Dalian National Laboratory for Clean Energy, Dalian Institute of Chemical Physics, Chinese Academy of Sciences, 116023, Dalian, Liaoning, China

E-mail: xwu2020@dicp.ac.cn

Leibniz-Institut für Katalyse e.V. an der, Institution Universität Rostock, Albert-Einstein-Straße 29a, Rostock 18059, Germany

E-mail: xiao-feng.wu@catalysis.de

Table of Contents

1. General Information 3

2. General Procedure 4

2.1 Synthesis of 2-Bromonitriles 4

2.2 Synthesis of Products 5

2.3 Scale-up Procedure 5

2.4 Synthesis of **4** and **5** 6

3. Characterization Data of Products 7

4. Characterization Data of **4** and **5** 17

5. Reference 18

6.Copy of ^{1}H and ^{13}C NMR Spectra of Products 19

7. Copy of ^{1}H NMR Spectra of **4** and **5** 46

1. General Information

Unless otherwise noted, all reactions were carried out under N_2 atmosphere. All reagents were from commercial sources and used as received without further purification. All solvents were dry solvents. Column chromatography was performed on silica gel (200-300 meshes) using dichloromethane and ethyl acetate as eluent. NMR spectra were recorded on a Bruker Avance operating at for ^{1}H NMR at 400 MHz, ^{13}C NMR at 101 MHz and spectral data were reported in ppm relative to tetramethylsilane (TMS) as internal standard and $CDCl_3$ (^{1}H NMR δ 7.26, ^{13}C NMR δ 77.16) as solvent. All coupling constants (*J*) are reported in Hz. The following abbreviations were used to describe peak splitting patterns when appropriate: s = singlet, d = doublet, dd = double doublet, ddd = double doublet of doublets, t = triplet, dt = double triplet, q = quatriplet, m = multiplet, br = broad. Gas chromatography (GC) analyses were performed on a Shimadzu GC-2014C chromatograph equipped with a FID detector. Mass spectra (MS) were measured on spectrometer by direct inlet at 70 eV. Mass spectroscopy data of the products were collected on an HRMS-TOF instrument or Waters TOFMS GCT Premier using EI or ESI ionization. Melting points were measured with WRR digital point apparatus and not corrected.

2. General Procedure

2.1 Synthesis of 2-Bromonitriles

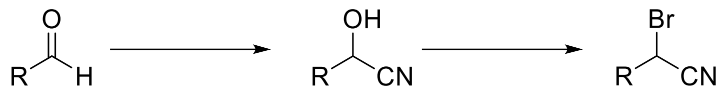

General procedure for the synthesis of cyanohydrins from aldehydes: Trimethylsilyl cyanide (4.50 mL, 30.0 mmol) was added to a solution of the aldehyde (30.0 mmol) and K_2CO_3 (0.830 g, 6.00 mmol) in Et_2O (60 mL) in a 250 mL round-bottom flask. The reaction mixture was stirred for 6 h at r.t., then the reaction was quenched by the addition of saturated aqueous $NaHCO_3$ (30 mL). The reaction mixture was extracted with Et_2O (2 × 20 mL), and the combined organic layer was concentrated. Next, an aqueous solution of HCl (1 M; 100 mL) was added to the residue, and the mixture was stirred for 2 h. Then, the reaction mixture was extracted with Et_2O (3 × 50 mL), and the combined organic layer was rinsed with saturated aqueous $NaHCO_3$ (50 mL) and brine (50 mL), dried over Na_2SO_4, and concentrated. The resulting product can proceed to the next reaction without purification.

General procedure for the synthesis of secondary bromides from cyanohydrins: Triphenylphosphine dibromide (15.2 g, 36.0 mmol) and then imidazole (2.45 g, 36.0 mmol) was added to a solution of the cyanohydrin (30.0 mmol) in dichloromethane (150 mL) at 0 °C. The solution was allowed to warm to r.t., then it was stirred for 6 h. Next, the reaction was quenched by the addition of saturated aqueous NH_4Cl (100 mL). The aqueous layer was extracted with dichloromethane (2 × 50 mL), and the combined organic layer was rinsed with brine (50 mL), dried over Na_2SO_4, and concentrated. The residue was purified by column chromatography (petroleum ether : ethyl acetate = 10 : 1 to 5 : 1).[1,2]

2.2 Synthesis of Products

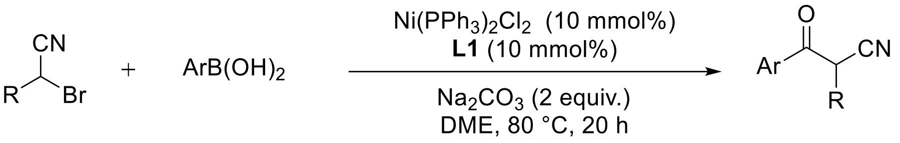

L1= 3,4,7,8-Tetramethyl-1,10-phenanthroline

1 **2** **3**

1 (0.2 mmol, 1.0 equiv.), **2** (0.3 mmol, 1.5 equiv.), $Ni(PPh_3)Cl_2$ (10 mol%), 3,4,7,8-Tetramethyl-1,10-phenanthroline (10 mol%), and Na_2CO_3(0.4 mmol, 2.0 equiv.) were added to an oven-dried tube (15 mL), which was then placed under vacuum and refilled with nitrogen for three times. Then dry DME (2.0 mL) was added into the tube via a syringe. A mixture of formic acid (2.0 mmol) and acetic anhydride (2.0 mmol), which was stirred at 30 °C for 1.5 h and then added to the small inner tube with Et_3N (2.0 mmol). The tube was sealed and the mixture was stirred at 80 °C (oil bath) for 20 h. After the reaction was completed, the reaction mixture was filtered and concentrated under vacuum. The crude product was purified by column chromatography (petroleum ether : ethyl acetate = 5 : 1 to 3 : 1) on silica gel to afford the corresponding products **3**.

2.3 Scale-up Procedure

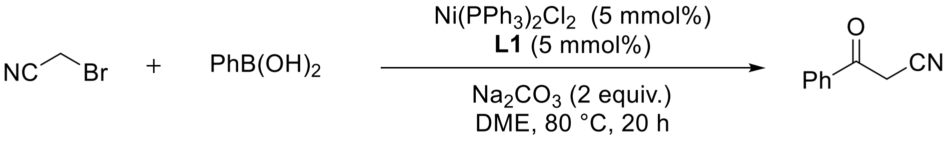

L1= 3,4,7,8-Tetramethyl-1,10-phenanthroline

1 **2** **3**

1 (1 mmol, 1.0 equiv.), **2** (1.5 mmol, 1.5 equiv.), $Ni(PPh_3)Cl_2$ (5 mol%), 3,4,7,8-Tetramethyl-1,10-phenanthroline (5 mol%), and Na_2CO_3(2 mmol, 2.0 equiv.) were added to an oven-dried tube (100 mL), which was then placed under vacuum and refilled with nitrogen for three times. Then dry DME (10 mL) was added into the tube via a syringe. A mixture of formic acid (10 mmol) and acetic anhydride (10 mmol), which was stirred at 30 °C for 1.5 h and then added to the small inner tube with Et_3N (1.0 mmol). The tube was sealed and the mixture was stirred at 80 °C (oil bath) for 20 h. After the reaction was completed, the reaction mixture was filtered and concentrated

under vacuum. The crude product was purified by column chromatography (petroleum ether : ethyl acetate = 5 : 1 to 3 : 1) on silica gel to afford the corresponding products **3** in 70% yield (101.5 mg).

2.4 Synthesis of 4 and 5

3-phenylisoxazol-5-amine

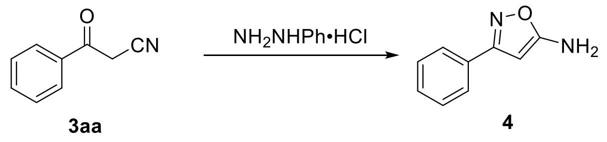

3aa (0.3 mmol), hydroxylamine hydrochloride (0.36 mmol, 1.2 equiv.), and sodium carbonate (0.6 mmol, 2equiv.) were added to a mixed solution of water (0.5 mL) and ethanol (2 mL), then heated to 100 °C for 16 hours. The reaction mixture was cooled to r.t., quenched with H_2O, extracted with EtOAc (2 x 10 mL) and dried over Na_2SO_4. The crude product was purified by column chromatography (petroleum ether : ethyl acetate = 3 : 1) on silica gel to afford the product **4** as a colorless solid.(25.5 mg, 53%)[1].

1,3-diphenyl-1*H*-pyrazol-5-amine

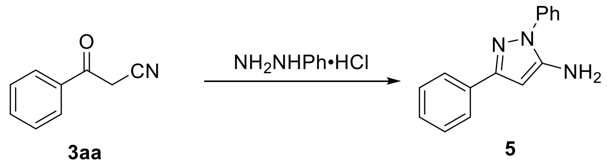

3aa (0.3 mmol), phenylhydrazine hydrochloride (0.36 mmol, 1.2 equiv.), and sodium carbonate (0.6 mmol, 2equiv.) were added to ethanol (2 mL), then heated to 120 °C for 16 hours. The reaction mixture was cooled to r.t., quenched with H_2O, extracted with EtOAc (2 x 10 mL) and dried over Na_2SO_4. The crude product was purified by column chromatography (petroleum ether : ethyl acetate = 3 : 1) on silica gel to afford the product **5** as a colorless solid.(39.5 mg, 56%)[1].

3. Characterization Data of Products

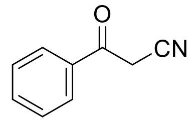

3-oxo-3-phenylpropanenitrile (3aa)[3]

Upon completion the mixture was concentrated and purified via flash column chromatography (petroleum ether : ethyl acetate = 3 : 1) to give the titled product **3aa** as a **yellow oil** (23.8 mg, 82%).

^{1}H NMR (400 MHz, CDCl$_3$) δ 7.92 (d, J = 7.4 Hz, 2H), 7.66 (t, J = 7.4 Hz, 1H), 7.52 (t, J = 7.8 Hz, 2H), 4.10 (s, 2H).

^{13}C NMR (101 MHz, CDCl$_3$) δ 187.1, 134.7, 134.2, 129.1, 128.4, 113.8, 29.4.

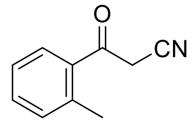

3-oxo-3-(*o*-tolyl)propanenitrile (3ab)[3]

Upon completion the mixture was concentrated and purified via flash column chromatography (petroleum ether : ethyl acetate = 5 : 1) to give the titled product **3ab** as a **yellow oil** (13.4 mg, 42%).

^{1}H NMR (400 MHz, CDCl$_3$) δ 7.62 (d, J = 7.8 Hz, 1H), 7.48 (dd, J = 11.3, 3.8 Hz, 1H), 7.33 (t, J = 6.9 Hz, 2H), 4.06 (s, 2H), 2.57 (s, 3H).

^{13}C NMR (101 MHz, CDCl$_3$) δ 189.2, 140.5, 133.7, 133.3, 132.8, 129.3, 126.1, 114.0, 31.4, 21.9.

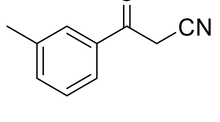

3-oxo-3-(*m*-tolyl)propanenitrile (3ac)[3]

Upon completion the mixture was concentrated and purified via flash column chromatography (petroleum ether : ethyl acetate = 5 : 1) to give the titled product **3ac** as a **yellow oil** (27.4 mg, 86%).

^{1}**H NMR (400 MHz, CDCl₃)** δ 7.72 – 7.69 (m, 2H), 7.47 (d, J = 7.5 Hz, 1H), 7.40 (t, J = 7.6 Hz, 1H), 4.08 (s, 2H), 2.42 (s, 3H).

^{13}**C NMR (101 MHz, CDCl₃)** δ 187.4, 139.2, 135.6, 134.3, 129.0, 128.0, 125.7, 114.0, 29.5, 21.4.

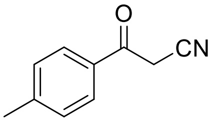

3-oxo-3-(*p*-tolyl)propanenitrile (3ad)[3]

Upon completion the mixture was concentrated and purified via flash column chromatography (petroleum ether : ethyl acetate = 5 : 1) to give the titled product **3ad** as a **yellow oil** (20.4 mg, 64%).

^{1}**H NMR (400 MHz, CDCl₃)** δ 7.81 (d, J = 8.2 Hz, 2H), 7.31 (d, J = 8.1 Hz, 2H), 4.06 (s, 2H), 2.44 (s, 3H).

^{13}**C NMR (101 MHz, CDCl₃)** δ 186.7, 146.0, 131.8, 129.9, 128.6, 114.0, 29.3, 21.9.

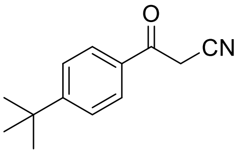

3-(4-(*tert*-butyl)phenyl)-3-oxopropanenitrile (3ae)[3]

Upon completion the mixture was concentrated and purified via flash column chromatography (petroleum ether : ethyl acetate = 5 : 1) to give the titled product **3ae** as a **yellow oil** (29.3 mg, 73%).

^{1}**H NMR (400 MHz, CDCl₃)** δ 7.85 (d, J = 8.6 Hz, 2H), 7.52 (d, J = 8.6 Hz, 2H), 4.07 (s, 2H), 1.34 (s, 9H).

^{13}**C NMR (101 MHz, CDCl₃)** δ 186.7, 158.8, 131.6, 128.4, 126.1, 114.0, 35.3, 30.9, 29.3.

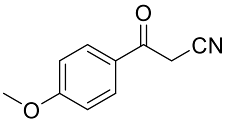

3-(4-methoxyphenyl)-3-oxopropanenitrile (3af)[3]

Upon completion the mixture was concentrated and purified via flash column chromatography (petroleum ether : ethyl acetate = 5 : 1) to give the titled product **3af**

as a **yellow oil** (21.4 mg, 61%).

^{1}H NMR (400 MHz, CDCl$_3$) δ 7.89 (d, J = 8.8 Hz, 2H), 6.97 (d, J = 8.9 Hz, 2H), 4.03 (s, 2H), 3.89 (s, 3H).

^{13}C NMR (101 MHz, CDCl$_3$) δ 185.4, 164.7, 130.9, 127.2, 114.3, 114.1, 55.6, 29.0.

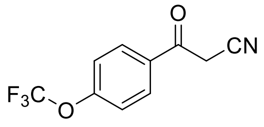

3-oxo-3-(4-(trifluoromethoxy)phenyl)propanenitrile (3ag)[3]

Upon completion the mixture was concentrated and purified via flash column chromatography (petroleum ether : ethyl acetate = 5 : 1) to give the titled product **3ag** as a **yellow oil** (20.6 mg, 45%).

^{1}H NMR (400 MHz, CDCl$_3$) δ 7.99 (d, J = 8.9 Hz, 2H), 7.35 (d, J = 8.2 Hz, 2H), 4.09 (s, 2H).

^{13}C NMR (101 MHz, CDCl$_3$) δ 185.6, 153.7, 132.2, 130.6, 120.7, 120.2 (d, J = 259.9 Hz), 113.4, 29.4.

^{19}F NMR (376 MHz, CDCl$_3$) δ -57.57.

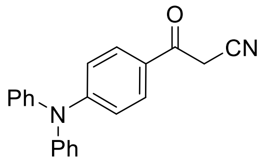

3-(4-(diphenylamino)phenyl)-3-oxopropanenitrile (3ah)[7]

Upon completion the mixture was concentrated and purified via flash column chromatography (petroleum ether : ethyl acetate = 5 : 1) to give the titled product **3ah** as a **yellow solid** (48.4 mg, 77%).

^{1}H NMR (400 MHz, CDCl$_3$) δ 7.72 (d, J = 9.0 Hz, 2H), 7.35 (t, J = 7.8 Hz, 4H), 7.21 – 7.16 (m, 6H), 6.96 (d, J = 9.0 Hz, 2H), 3.97 (s, 2H).

^{13}C NMR (101 MHz, CDCl$_3$) δ 184.7, 153.3, 145.7, 130.2, 129.7, 126.4, 125.4, 118.6, 114.3, 28.8.

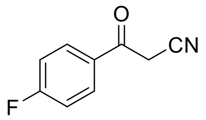

3-(4-fluorophenyl)-3-oxopropanenitrile (3ai)[3]

Upon completion the mixture was concentrated and purified via flash column chromatography (petroleum ether : ethyl acetate = 5 : 1) to give the titled product **3ai** as a **yellow oil** (23.5 mg, 72%).

^{1}H NMR (400 MHz, $CDCl_3$) δ 7.98 – 7.95 (m, 2H), 7.20 (t, J = 8.5 Hz, 2H), 4.08 (s, 2H).

^{13}C NMR (101 MHz, $CDCl_3$) δ 185.6, 166.6 (d, J = 258.1 Hz), 131.3 (d, J = 9.7 Hz), 130.7 (d, J = 2.8 Hz), 116.4 (d, J = 22.2 Hz), 113.6, 29.4.

^{19}F NMR (376 MHz, $CDCl_3$) δ -101.57.

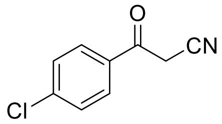

3-(4-chlorophenyl)-3-oxopropanenitrile (3aj)[3]

Upon completion the mixture was concentrated and purified via flash column chromatography (petroleum ether : ethyl acetate = 5 : 1) to give the titled product **3aj** as a **yellow oil** (26.5 mg, 74%).

^{1}H NMR (400 MHz, $CDCl_3$) δ 7.86 (d, J = 8.6 Hz, 2H), 7.50 (d, J = 8.6 Hz, 2H), 4.08 (s, 2H).

^{13}C NMR (101 MHz, $CDCl_3$) δ 186.0, 141.4, 132.5, 129.8, 129.5, 113.5, 29.4.

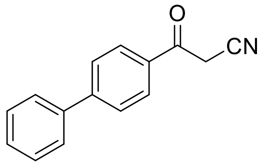

3-([1,1'-biphenyl]-4-yl)-3-oxopropanenitrile (3ak)[3]

Upon completion the mixture was concentrated and purified via flash column chromatography (petroleum ether : ethyl acetate = 5 : 1) to give the titled product **3ak** as a **yellow oil** (39.8 mg, 90%).

^{1}H NMR (400 MHz, CDCl$_3$) δ 7.99 (d, J = 8.5 Hz, 2H), 7.73 (d, J = 8.5 Hz, 2H), 7.63 (d, J = 7.1 Hz, 2H), 7.51 – 7.42 (m, 3H), 4.12 (s, 2H).

^{13}C NMR (101 MHz, CDCl$_3$) δ 186.6, 147.3, 139.1, 132.8, 129.0, 128.7, 127.6, 127.2, 113.9, 29.4.

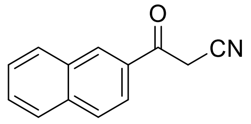

3-(naphthalen-2-yl)-3-oxopropanenitrile (3al)[3]

Upon completion the mixture was concentrated and purified via flash column chromatography (petroleum ether : ethyl acetate = 5 : 1) to give the titled product **3al** as a **yellow oil** (29.6 mg, 76%).

^{1}H NMR (400 MHz, CDCl$_3$) δ 8.39 (s, 1H), 7.98 – 7.89 (m, 4H), 7.68 – 7.58 (m, 2H), 4.22 (s, 2H).

^{13}C NMR (101 MHz, CDCl$_3$) δ 187.0, 136.1, 132.2, 131.5, 130.7, 129.7, 129.5, 129.1, 127.87, 127.4, 123.3, 114.0, 29.4.

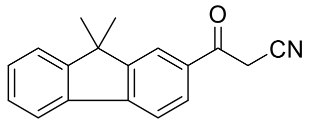

3-(9,9-dimethyl-9*H*-fluoren-2-yl)-3-oxopropanenitrile (3am)[7]

Upon completion the mixture was concentrated and purified via flash column chromatography (petroleum ether : ethyl acetate = 5 : 1) to give the titled product **3am** as a **yellow solid** (43.8 mg, 84%).

^{1}H NMR (400 MHz, CDCl$_3$) δ 8.02 (d, J = 1.2 Hz, 1H), 7.89 (dd, J = 8.0, 1.6 Hz, 1H), 7.82 – 7.79 (m, 2H), 7.50 – 7.48 (m, 1H), 7.45 – 7.37 (m, 2H), 4.16 (s, 2H), 1.52 (s, 6H).

^{13}C NMR (101 MHz, CDCl$_3$) δ 186.8, 154.9, 154.3, 145.8, 137.2, 132.9, 129.2, 128.2, 127.4, 122.9, 122.7, 121.3, 120.2, 114.1, 47.1, 29.5, 26.8.

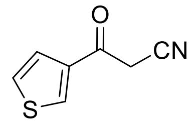

3-oxo-3-(thiophen-3-yl)propanenitrile (3an)[3]

Upon completion the mixture was concentrated and purified via flash column chromatography (petroleum ether : ethyl acetate = 5 : 1) to give the titled product **3an** as a **yellow oil** (21.4 mg, 71%).

^{1}H NMR (400 MHz, CDCl$_3$) δ 8.16 (dd, J = 2.8, 1.2 Hz, 1H), 7.55 (dd, J = 5.1, 1.1 Hz, 1H), 7.41 (dd, J = 5.1, 2.9 Hz, 1H), 3.99 (s, 2H).

^{13}C NMR (101 MHz, CDCl$_3$) δ 180.9, 139.2, 134.0, 127.6, 126.7, 113.7, 30.1.

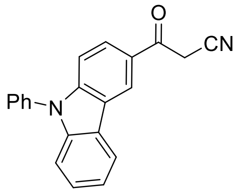

3-oxo-3-(9-phenyl-9*H*-carbazol-3-yl)propanenitrile (3ao)[7]

Upon completion the mixture was concentrated and purified via flash column chromatography (petroleum ether : ethyl acetate = 5 : 1) to give the titled product **3ao** as a **yellow solid** (43.4 mg, 70%).

^{1}H NMR (400 MHz, CDCl$_3$) δ 8.76 (d, J = 1.5 Hz, 1H), 8.24 (d, J = 7.8 Hz, 1H), 8.00 (dd, J = 8.7, 1.8 Hz, 1H), 7.71 – 7.67 (m, 2H), 7.60 – 7.56 (m, 3H), 7.55 – 7.51 (m, 1H), 7.45 – 7.40 (m, 3H), 4.24 (s, 2H).

^{13}C NMR (101 MHz, CDCl$_3$) δ 186.2, 144.4, 142.0, 136.5, 130.2, 128.5, 127.3, 127.1, 126.6, 123.5, 123.1, 122.1, 121.4, 120.7, 114.4, 110.6, 110.1, 29.4.

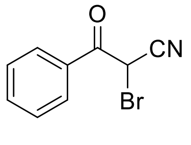

2-bromo-3-oxo-3-phenylpropanenitrile (3ba)[6]

Upon completion the mixture was concentrated and purified via flash column chromatography (petroleum ether : ethyl acetate = 5 : 1) to give the titled product **3ba** as a **yellow oil** (8.9 mg, 20%).

^{1}H NMR (400 MHz, CDCl$_3$) δ 7.93 (d, J = 8.0 Hz, 2H), 7.67 (t, J = 7.4 Hz, 1H), 7.53 (t, J = 7.8 Hz, 2H), 4.09 (s, 2H).

^{13}C NMR (101 MHz, CDCl$_3$) δ 187.1, 134.7, 129.1, 128.4, 113.8, 29.4.

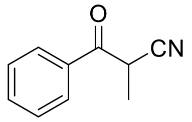

2-methyl-3-oxo-3-phenylpropanenitrile (3ca)[4]

Upon completion the mixture was concentrated and purified via flash column chromatography (petroleum ether : ethyl acetate = 5 : 1) to give the titled product **3ca** as a **yellow oil** (30.8 mg, 97%).

^{1}H NMR (400 MHz, CDCl$_3$) δ 7.98 (d, J = 7.2 Hz, 2H), 7.65 (t, J = 7.4 Hz, 1H), 7.53 (t, J = 7.7 Hz, 2H), 4.39 (q, J = 7.2 Hz, 1H), 1.64 (d, J = 7.2 Hz, 3H).

^{13}C NMR (101 MHz, CDCl$_3$) δ 190.7, 134.5, 133.6, 129.1, 128.8, 118.1, 33.7, 14.9.

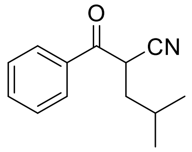

2-benzoyl-4-methylpentanenitrile (3da)[4]

Upon completion the mixture was concentrated and purified via flash column chromatography (petroleum ether : ethyl acetate = 5 : 1) to give the titled product **3da** as a **yellow oil** (38.6 mg, 96%).

^{1}H NMR (400 MHz, CDCl$_3$) δ 7.95 (d, J = 7.3 Hz, 2H), 7.64 (t, J = 7.4 Hz, 1H), 7.52 (t, J = 7.8 Hz, 2H), 4.40 (dd, J = 9.8, 5.2 Hz, 1H), 1.97-1.90 (m, J = 11.4, 3.6 Hz, 2H), 1.81 – 1.74 (m, 1H), 1.01 (dd, J = 13.2, 6.4 Hz, 6H).

^{13}C NMR (101 MHz, CDCl$_3$) δ 191.1, 134.5, 134.0, 129.2, 128.8, 117.5, 38.6, 38.4, 26.5, 22.8, 21.4.

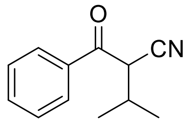

2-benzoyl-3-methylbutanenitrile (3ea)[4]

Upon completion the mixture was concentrated and purified via flash column chromatography (petroleum ether : ethyl acetate = 5 : 1) to give the titled product **3ea**

as a **yellow oil** (35.7 mg, 95%).

^{1}H NMR (400 MHz, CDCl$_3$) δ 7.92 (d, J = 7.2 Hz, 2H), 7.64 (t, J = 7.4 Hz, 1H), 7.51 (t, J = 7.8 Hz, 2H), 4.32 (d, J = 5.4 Hz, 1H), 2.46 – 2.42 (m, 1H), 1.17 (d, J = 6.8 Hz, 3H), 1.09 (d, J = 6.7 Hz, 3H).

^{13}C NMR (101 MHz, CDCl$_3$) δ 191.2, 134.3, 129.0, 128.6, 116.2, 47.8, 29.8, 21.3, 18.7.

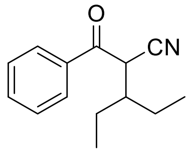

2-benzoyl-3-ethylpentanenitrile (3fa)[5]

Upon completion the mixture was concentrated and purified via flash column chromatography (petroleum ether : ethyl acetate = 5 : 1) to give the titled product **3fa** as a **yellow oil** (38.7 mg, 90%).

^{1}H NMR (400 MHz, CDCl$_3$) δ 7.90 (d, J = 7.9 Hz, 2H), 7.63 (t, J = 7.4 Hz, 1H), 7.51 (t, J = 7.7 Hz, 2H), 4.54 (d, J = 4.8 Hz, 1H), 2.04 – 1.96 (m, 1H), 1.74 – 1.65 (m, 1H), 1.61 – 1.54 (m, 1H), 1.50 – 1.42 (m, 2H), 1.04 (t, J = 7.4 Hz, 3H), 0.86 (t, J = 7.5 Hz, 3H).

^{13}C NMR (101 MHz, CDCl$_3$) δ 191.7, 134.3, 134.2, 129.0, 128.5, 116.2, 44.3, 42.2, 24.2, 23.4, 11.3, 10.9.

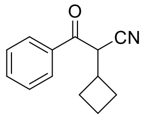

2-cyclobutyl-3-oxo-3-phenylpropanenitrile (3ga)[4]

Upon completion the mixture was concentrated and purified via flash column chromatography (petroleum ether : ethyl acetate = 5 : 1) to give the titled product **3ha** as a **yellow oil** (31.8 mg, 80%).

^{1}H NMR (400 MHz, CDCl$_3$) δ 7.93 (d, J = 7.4 Hz, 2H), 7.64 (t, J = 7.4 Hz, 1H), 7.51 (t, J = 7.7 Hz, 2H), 4.44 (d, J = 6.7 Hz, 1H), 3.01 – 2.95 (m, 1H), 2.14 – 1.89 (m, 6H).

^{13}C NMR (101 MHz, CDCl$_3$) δ 190.1, 134.3, 134.2, 129.0, 128.6, 116.4, 45.5, 35.0, 26.9, 25.8, 18.0.

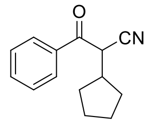

2-cyclopentyl-3-oxo-3-phenylpropanenitrile (3ha)[6]

Upon completion the mixture was concentrated and purified via flash column chromatography (petroleum ether : ethyl acetate = 5 : 1) to give the titled product **3ia** as a **yellow oil** (40.5 mg, 95%).

^{1}H NMR (400 MHz, CDCl$_3$) δ 7.95 (d, J = 7.3 Hz, 2H), 7.64 (t, J = 7.4 Hz, 1H), 7.51 (t, J = 7.9 Hz, 2H), 4.42 (d, J = 6.6 Hz, 1H), 2.58 – 2.48 (m, 1H), 1.93 – 1.84 (m, 2H), 1.75 – 1.69 (m, 2H), 1.59 – 1.51 (m, 3H), 1.44 – 1.35 (m, 1H).

^{13}C NMR (101 MHz, CDCl$_3$) δ 191.0, 134.3, 129.0, 128.6, 116.8, 45.0, 40.1, 31.2, 29.6, 25.0, 24.8.

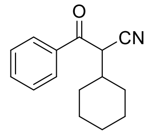

2-cyclohexyl-3-oxo-3-phenylpropanenitrile (3ia)[5]

Upon completion the mixture was concentrated and purified via flash column chromatography (petroleum ether : ethyl acetate = 5 : 1) to give the titled product **3ja** as a **yellow oil** (38.6 mg,85%).

^{1}H NMR (400 MHz, CDCl$_3$) δ 7.93 (d, J = 7.3 Hz, 2H), 7.65 (t, J = 7.4 Hz, 1H), 7.52 (t, J = 7.7 Hz, 2H), 4.29 (d, J = 5.9 Hz, 1H), 2.13 – 2.06 (m, 1H), 1.86 – 1.75 (m, 4H), 1.36 – 1.15 (m, 6H).

^{13}C NMR (101 MHz, CDCl$_3$) δ 191.0, 134.5, 134.3, 129.1, 128.6, 116.5, 47.0, 38.9, 31.8, 29.3, 25.9, 25.6, 25.4.

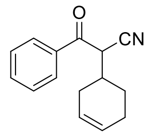

2-(cyclohex-3-en-1-yl)-3-oxo-3-phenylpropanenitrile (3ja)[5]

Upon completion the mixture was concentrated and purified via flash column chromatography (petroleum ether : ethyl acetate = 5 : 1) to give the titled product **3ja**

as a **yellow oil** (38.7 mg,86%).

^{1}H NMR (400 MHz, CDCl$_3$) δ 7.96 – 7.93 (m, 2H), 7.65 (t, J = 7.4 Hz, 1H), 7.52 (t, J = 7.8 Hz, 2H), 5.72 – 5.57 (m, 2H), 4.40 (dd, J = 15.0, 6.4 Hz, 1H), 2.45 – 2.38 (m, 1H), 2.12 – 1.92 (m, 5H), 1.68 – 1.58 (m, 1H).

^{13}C NMR (101 MHz, CDCl$_3$) δ 190.8, 134.4, 134.4, 134.4, 129.1, 129.1, 128.7, 128.6, 127.2, 126.5, 124.7, 124.4, 116.4, 116.2, 46.2, 45.1, 34.9, 34.8, 29.7, 28.0, 27.3, 25.2, 24.7, 24.3.

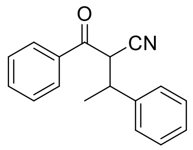

2-benzoyl-3-phenylbutanenitrile (3ga)[8]

Upon completion the mixture was concentrated and purified via flash column chromatography (petroleum ether : ethyl acetate = 5 : 1) to give the titled product **3ga** as a **yellow oil** (37.4 mg, 75%).

^{1}H NMR (400 MHz, CDCl$_3$) δ 7.92 – 7.86 (m, 2H), 7.67 – 7.60 (m, 1H), 7.53 – 7.46 (m, 2H), 7.32 – 7.24 (m, 5H), 4.62 – 4.56 (m, 1H), 3.67 – 3.55 (m, 1H), 1.56 – 1.46 (m, 3H).

^{13}C NMR (101 MHz, CDCl$_3$) δ 190.6, 190.4, 141.7, 140.3, 134.8, 134.4, 134.3, 134.2, 129.0, 129.0, 128.9, 128.7, 128.7, 128.6, 127.8, 127.7, 127.5, 127.0, 116.4, 116.2, 47.7, 47.5, 40.8, 39.7, 19.7, 17.8.

4. Characterization Data of 4 and 5

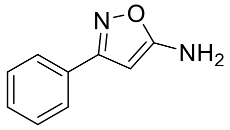

3-phenylisoxazol-5-amine (4)

Upon completion the mixture was concentrated and purified via flash column chromatography (petroleum ether : ethyl acetate = 3 : 1) to give the titled product **4** as a colorless solid(25.5 mg, 53%).[1]

^{1}H NMR (400 MHz, CDCl$_3$) δ 7.74 – 7.71 (m, 2H), 7.43 – 7.41 (m, 3H), 5.43 (s, 1H), 4.61 (s, 2H).

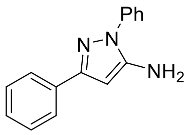

1,3-diphenyl-1*H*-pyrazol-5-amine (5)

Upon completion the mixture was concentrated and purified via flash column chromatography (petroleum ether : ethyl acetate = 3 : 1) to give the titled product **5** as a colorless solid.(39.5 mg, 56%).[1]

·**^{1}H NMR (400 MHz, CDCl$_3$)** δ 7.82 (d, J = 7.1 Hz, 2H), 7.64 (d, J = 7.4 Hz, 2H), 7.50 (t, J = 7.9 Hz, 2H), 7.41 – 7.29 (m, 4H), 5.96 (s, 1H), 3.85 (s, 2H).

5. Reference

[1] Donslund, A. S.; Neumann, K. T.; Corneliussen, N. P.; Grove, E. K.; Herbstritt, D.; Daasbjerg, K.; Skrydstrup, T. Access to *β*-Ketonitriles through Nickel-Catalyzed Carbonylative Coupling of *α*-Bromonitriles with Alkylzinc Reagents. *Chem. Eur. J.* **2019**, *25*, 9856–9860.

[2] Choi, J.; Fu, G. C. Catalytic Asymmetric Synthesis of Secondary Nitriles via Stereoconvergent Negishi Arylations and Alkenylations of Racemic *α*-Bromonitriles. *J. Am. Chem. Soc.* **2012**, *134*, 9102–9105.

[3] Zeng, G.; Liu, J.; Shao, Y.; Zhang, F.; Chen, Z.; Lv, N.; Chen, J.; Li, R. Selective Synthesis of *β*-Ketonitriles via Catalytic Carbopalladation of Dinitriles. *J. Org. Chem.* **2021**, *86*, 861–867.

[4] Kiyokawa, K.; Nagata, T.; Minakata, S. Electrophilic Cyanation of Boron Enolates: Efficient Access to Various *β*-Ketonitrile Derivatives. *Angew. Chem. Int. Ed.* **2016**, *55*, 10458–910462.

[5] Ji, Y.; Trenkl, W. C.; Vowles, J. V. A High-Yielding Preparation of *β*-Ketonitriles. *Org. Lett.* **2006**, *8*, 1161–1163

[6] Attanasi, O.; Berretta, S.; Favi, G.; Filippone, P.; Mele, G.; Moscatelli, G.; Saladino, R. Tetrabromo Hydrogenated Cardanol: Efficient and Renewable Brominating Agent. *Org. Lett*. **2006**, *8*, 4291-4293.

[7] You, J.; Lai, S.-L.; Liu, W.; Ng, T.-W.; Wang, P.; Lee, C.-S. Bipolar cyano-substituted pyridine derivatives for applications in organic light-emitting devices. *J. Materials Chem.*, **2012**, *22*, 8922-8929.

[8] Jia, Q.; Wang, W.; Lin, Y. Preparation method of polysubstituted [alpha]-ketoester. **2021**, CN112679383, Chinese Patent.

6.Copy of ^{1}H and ^{13}C NMR Spectra of Products

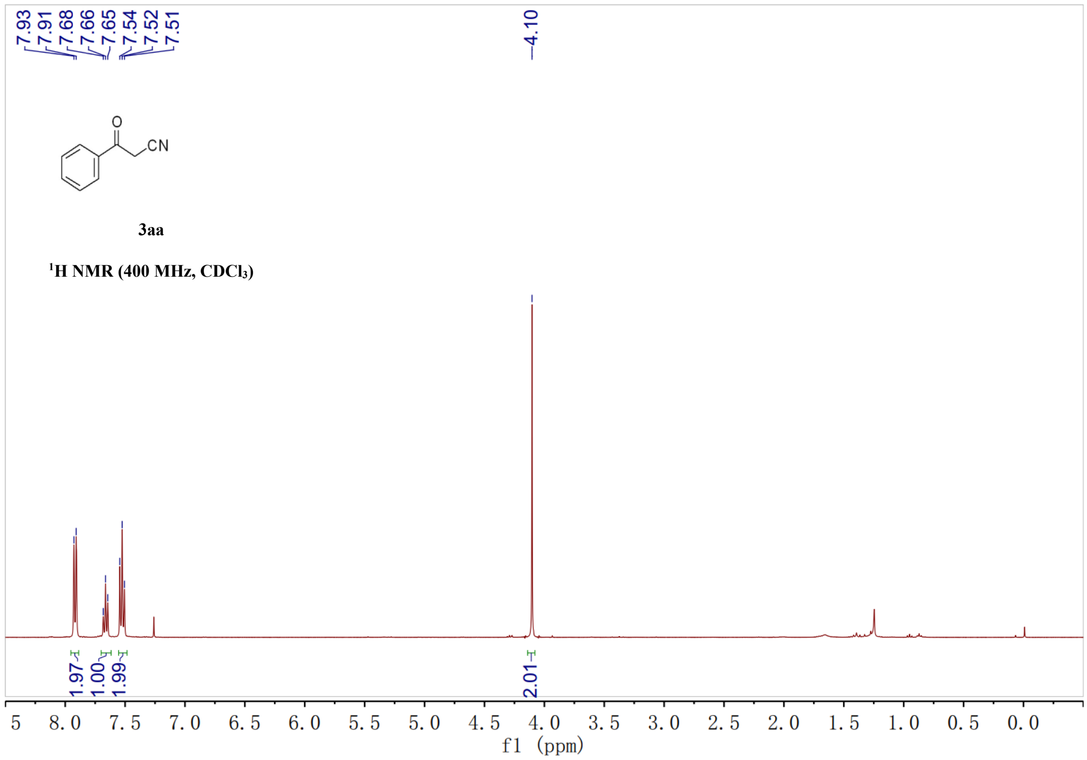

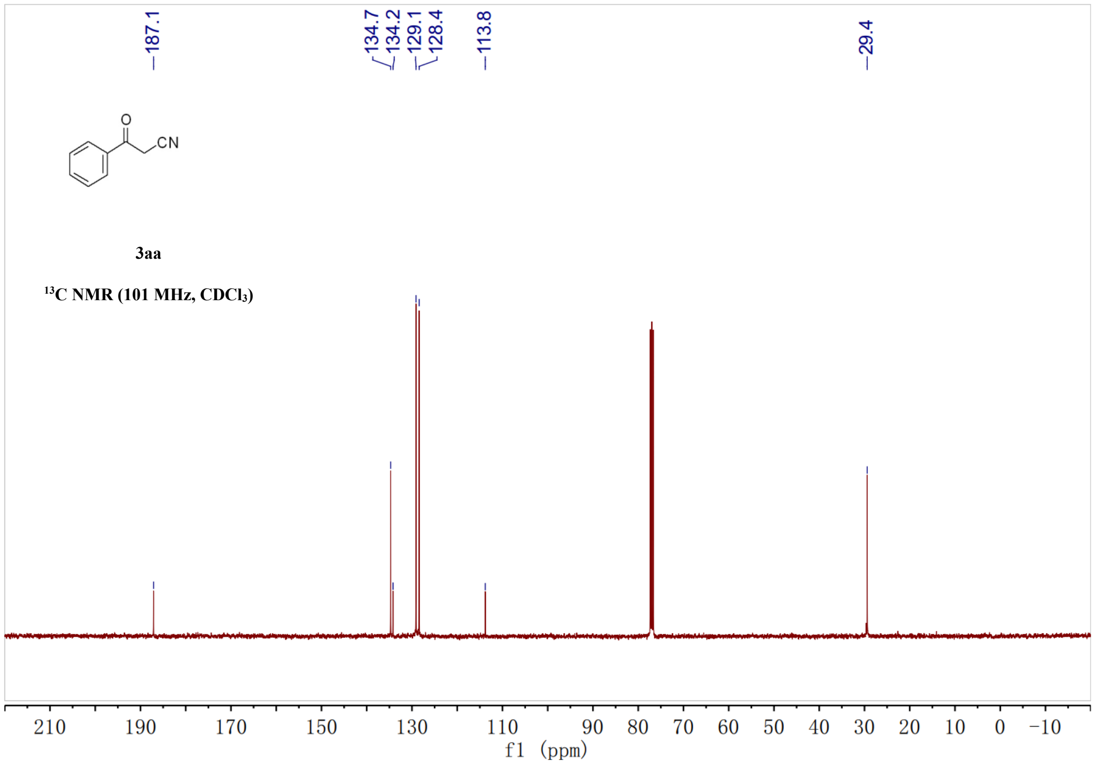

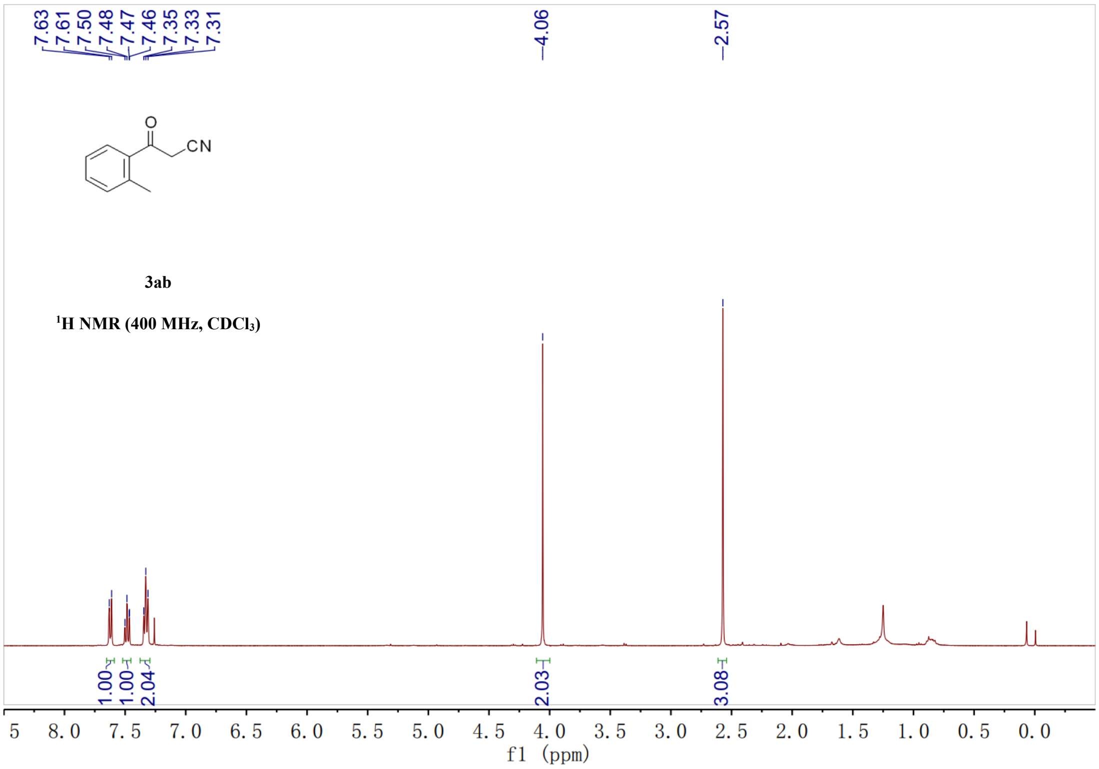
7.63
7.61
7.50
7.48
7.47
7.46
7.35
7.33
7.31
4.06
2.57
O
CN
3ab
1H NMR (400 MHz, CDCl3)
1.00
1.00
2.04
2.03
3.08
f1 (ppm)

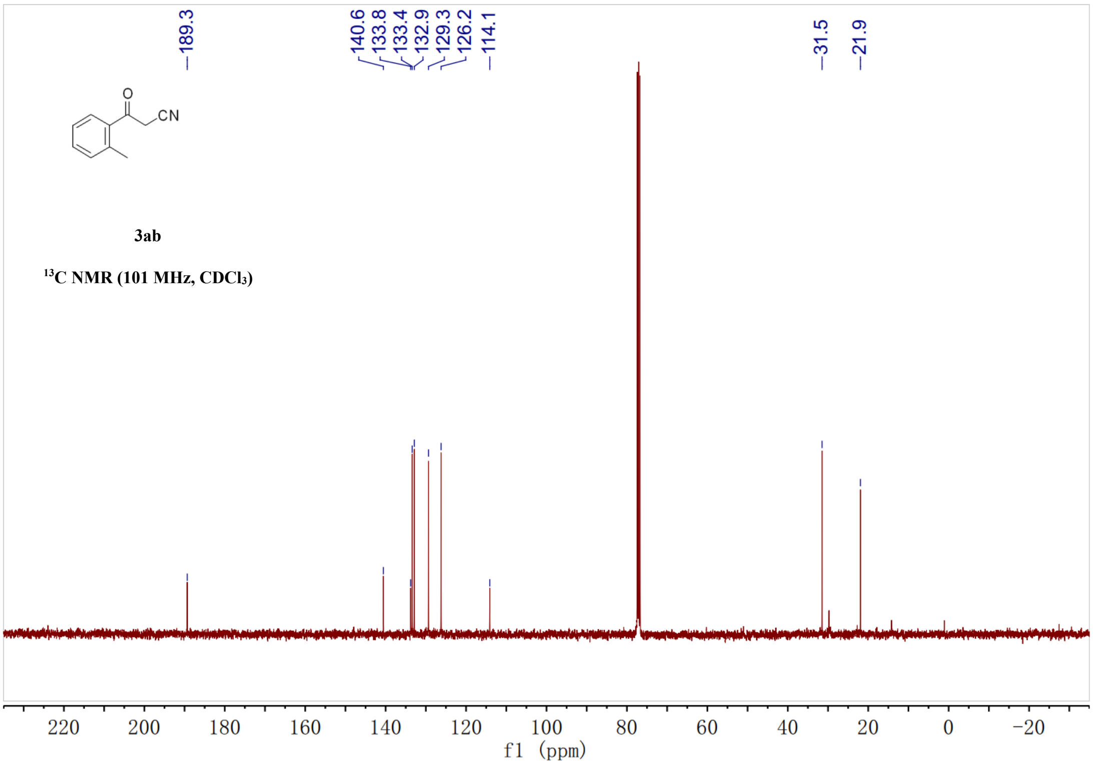
189.3
140.6
133.8
133.4
132.9
129.3
126.2
114.1
31.5
21.9
O
CN
3ab
13C NMR (101 MHz, CDCl3)
f1 (ppm)

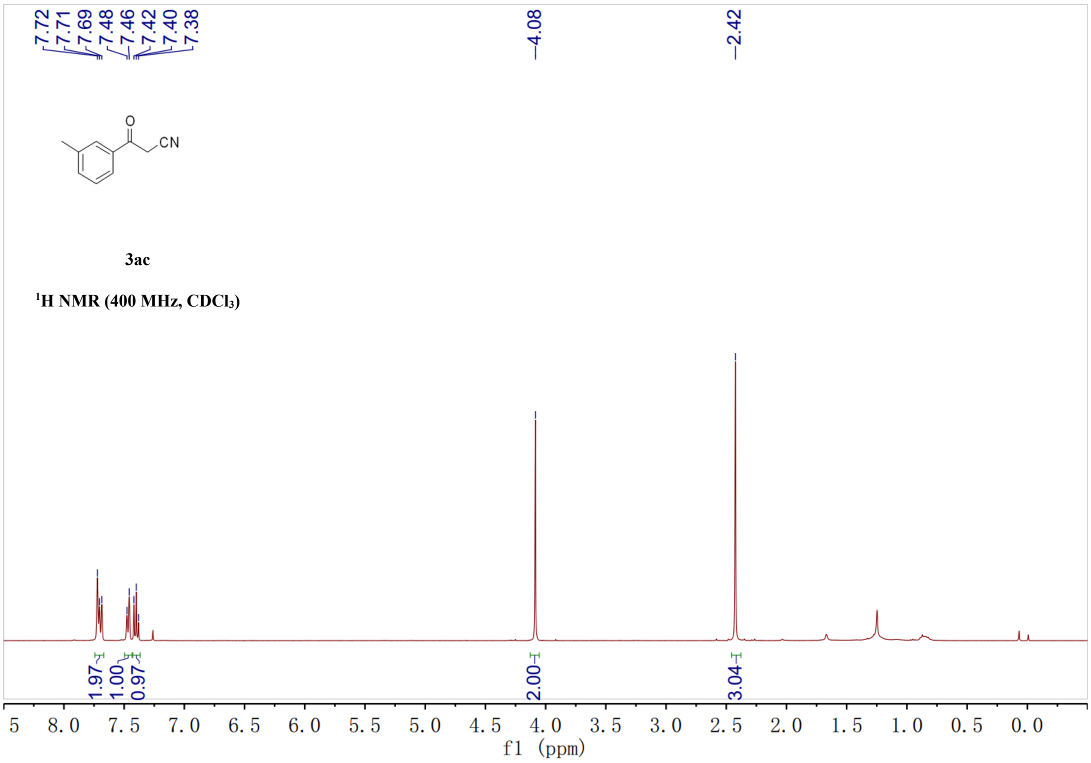
7.72
7.71
7.69
7.48
7.46
7.42
7.40
7.38
4.08
2.42
O
CN
3ac
1H NMR (400 MHz, CDCl3)
1.97
1.00
0.97
2.00
3.04
5 8.0 7.5 7.0 6.5 6.0 5.5 5.0 4.5 4.0 3.5 3.0 2.5 2.0 1.5 1.0 0.5 0.0
f1 (ppm)

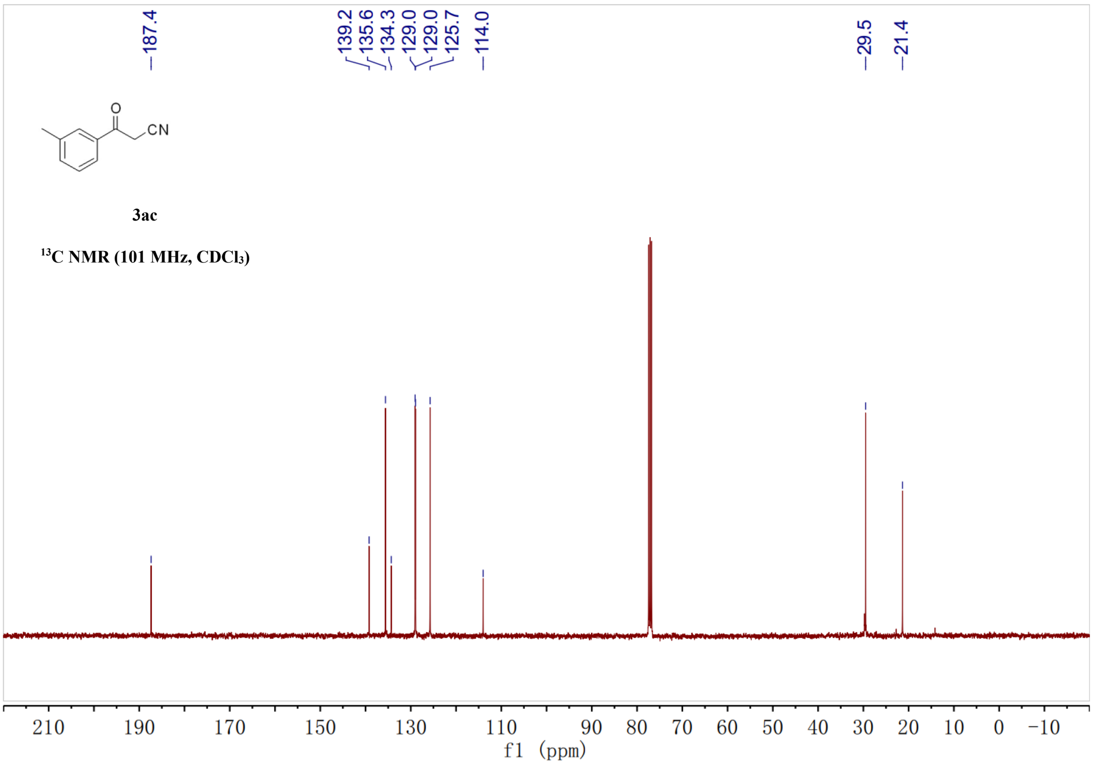
187.4
139.2
135.6
134.3
129.0
129.0
125.7
114.0
29.5
21.4
O
CN
3ac
13C NMR (101 MHz, CDCl3)
210 190 170 150 130 110 90 80 70 60 50 40 30 20 10 0 −10
f1 (ppm)

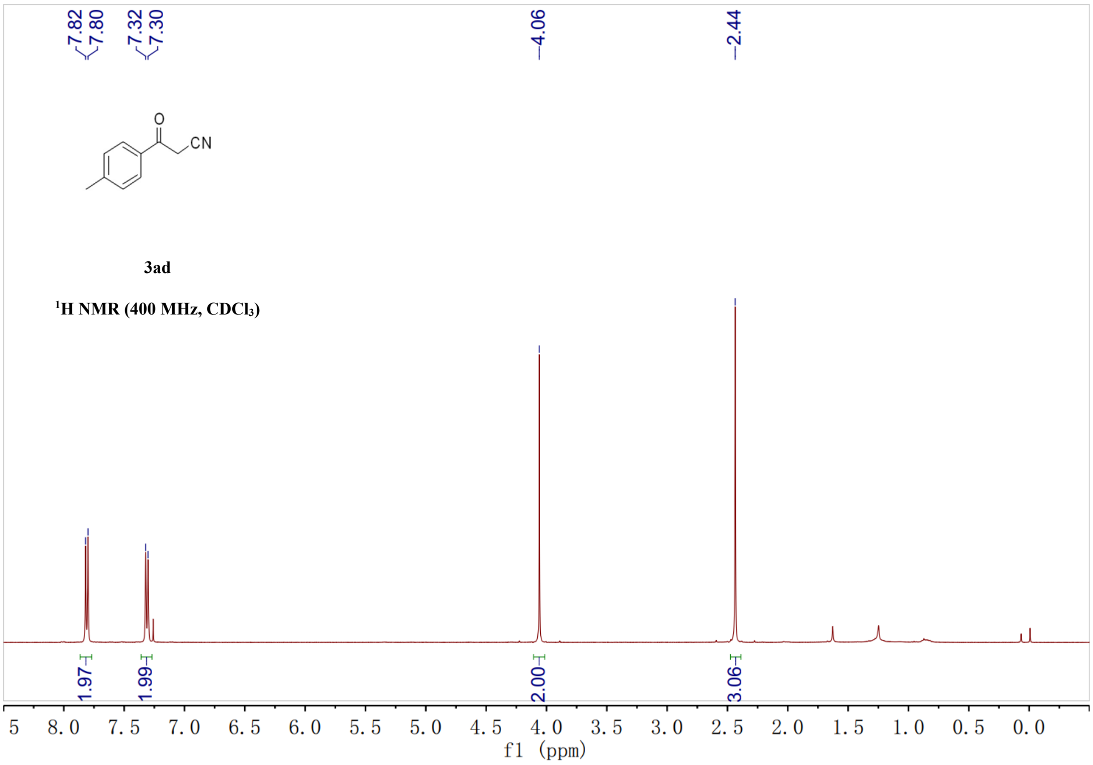
7.82
7.80
7.32
7.30
4.06
2.44
O
CN
3ad
1H NMR (400 MHz, CDCl3)
1.97
1.99
2.00
3.06
5 8.0 7.5 7.0 6.5 6.0 5.5 5.0 4.5 4.0 3.5 3.0 2.5 2.0 1.5 1.0 0.5 0.0
f1 (ppm)

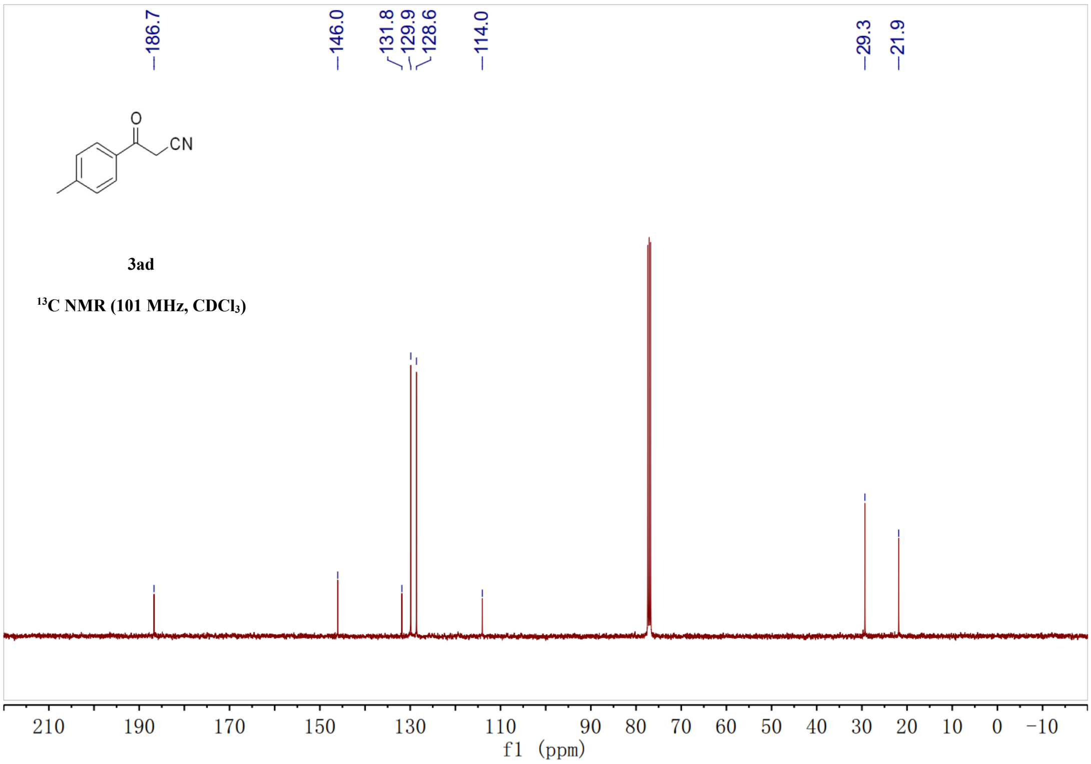
186.7
146.0
131.8
129.9
128.6
114.0
29.3
21.9
O
CN
3ad
13C NMR (101 MHz, CDCl3)
210 190 170 150 130 110 90 80 70 60 50 40 30 20 10 0 −10
f1 (ppm)

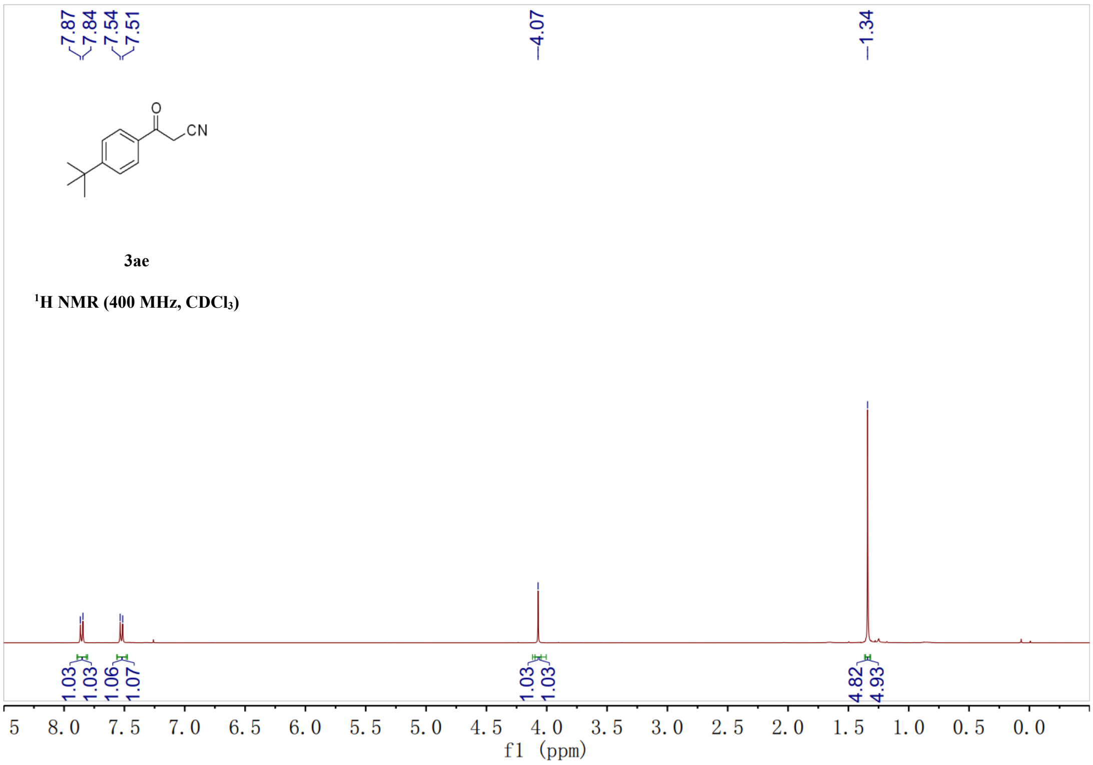
7.87
7.84
7.54
7.51
4.07
1.34
CN
O
3ae
^{1}H NMR (400 MHz, CDCl$_3$)
1.03
1.03
1.06
1.07
1.03
1.03
4.82
4.93
5 8.0 7.5 7.0 6.5 6.0 5.5 5.0 4.5 4.0 3.5 3.0 2.5 2.0 1.5 1.0 0.5 0.0
f1 (ppm)

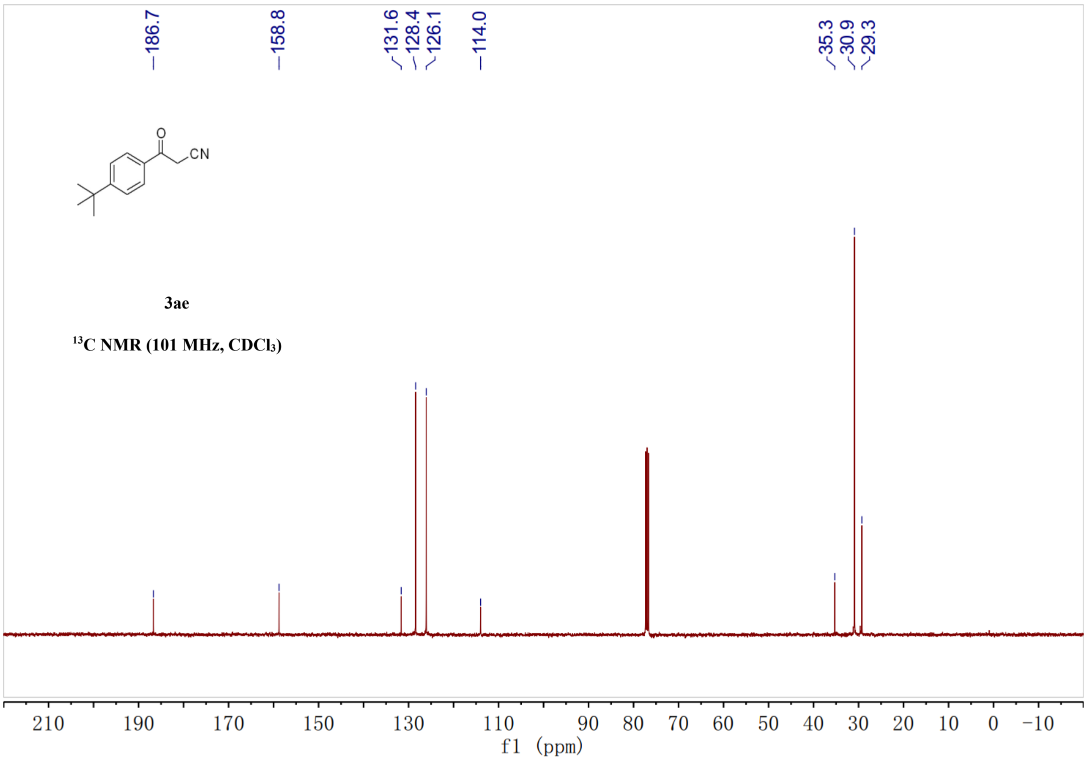
186.7
158.8
131.6
128.4
126.1
114.0
35.3
30.9
29.3
CN
O
3ae
^{13}C NMR (101 MHz, CDCl$_3$)
210 190 170 150 130 110 90 80 70 60 50 40 30 20 10 0 −10
f1 (ppm)

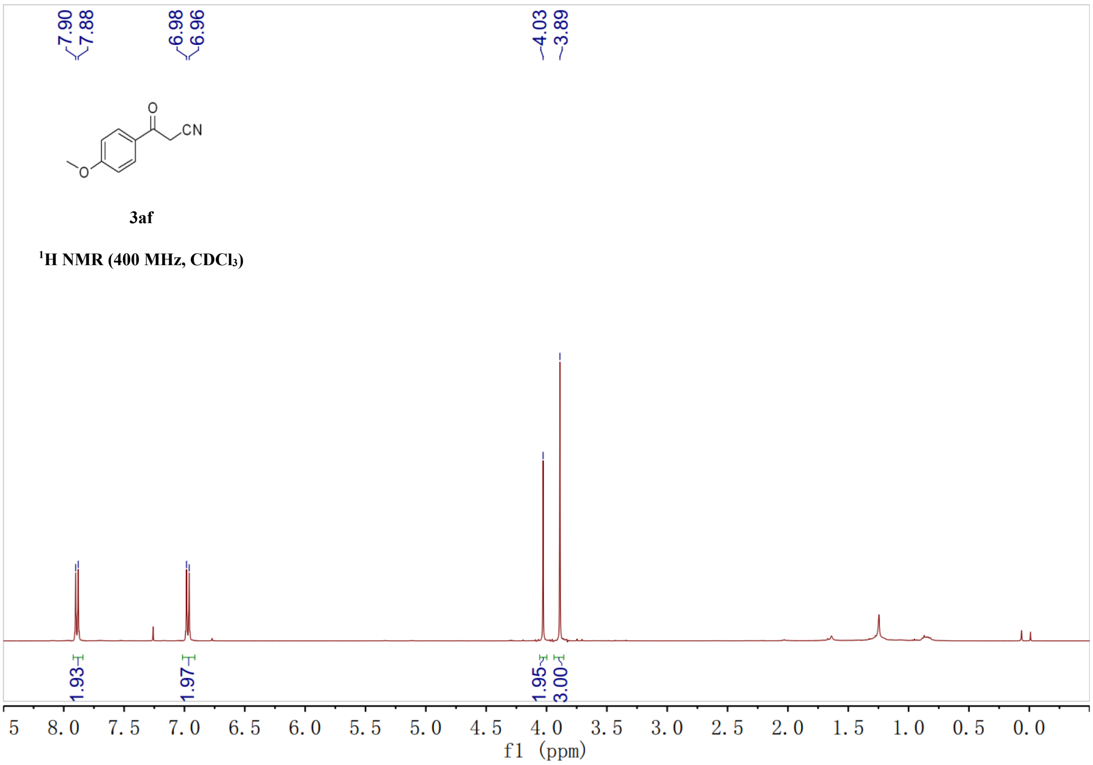
7.90
7.88
6.98
6.96
4.03
3.89
O
CN
O
3af
¹H NMR (400 MHz, CDCl₃)
1.93
1.97
1.95
3.00
8.0 7.5 7.0 6.5 6.0 5.5 5.0 4.5 4.0 3.5 3.0 2.5 2.0 1.5 1.0 0.5 0.0
f1 (ppm)

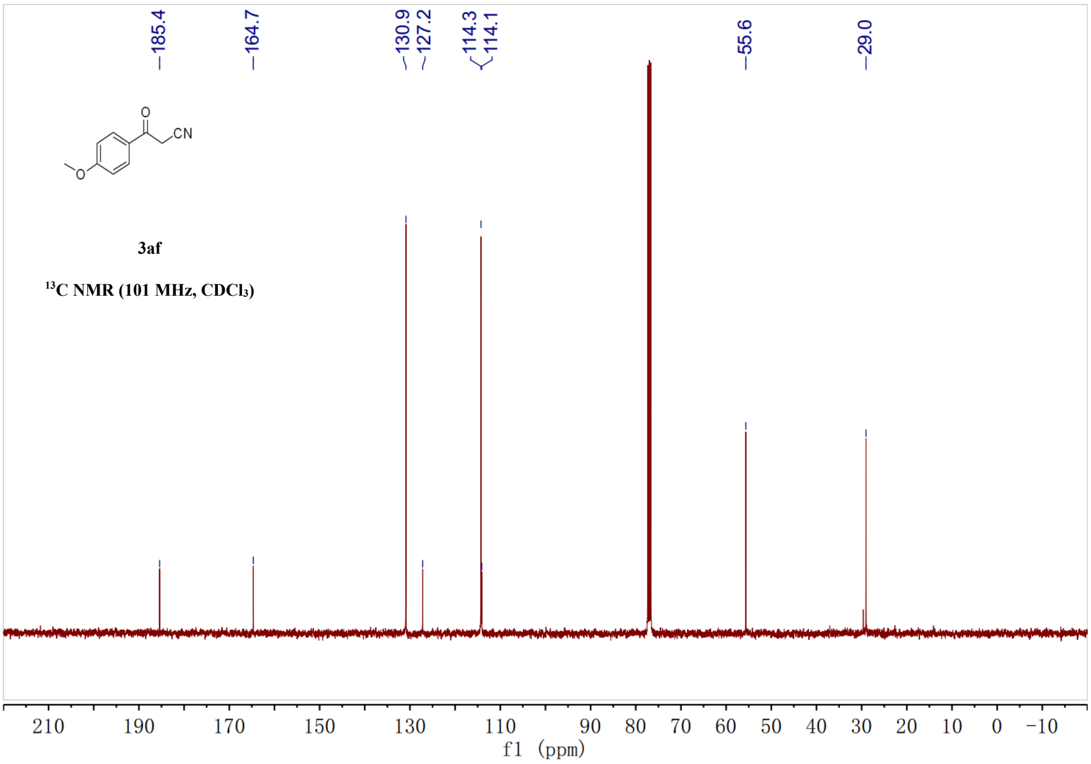
185.4
164.7
130.9
127.2
114.3
114.1
55.6
29.0
O
CN
O
3af
¹³C NMR (101 MHz, CDCl₃)
210 190 170 150 130 110 90 80 70 60 50 40 30 20 10 0 −10
f1 (ppm)

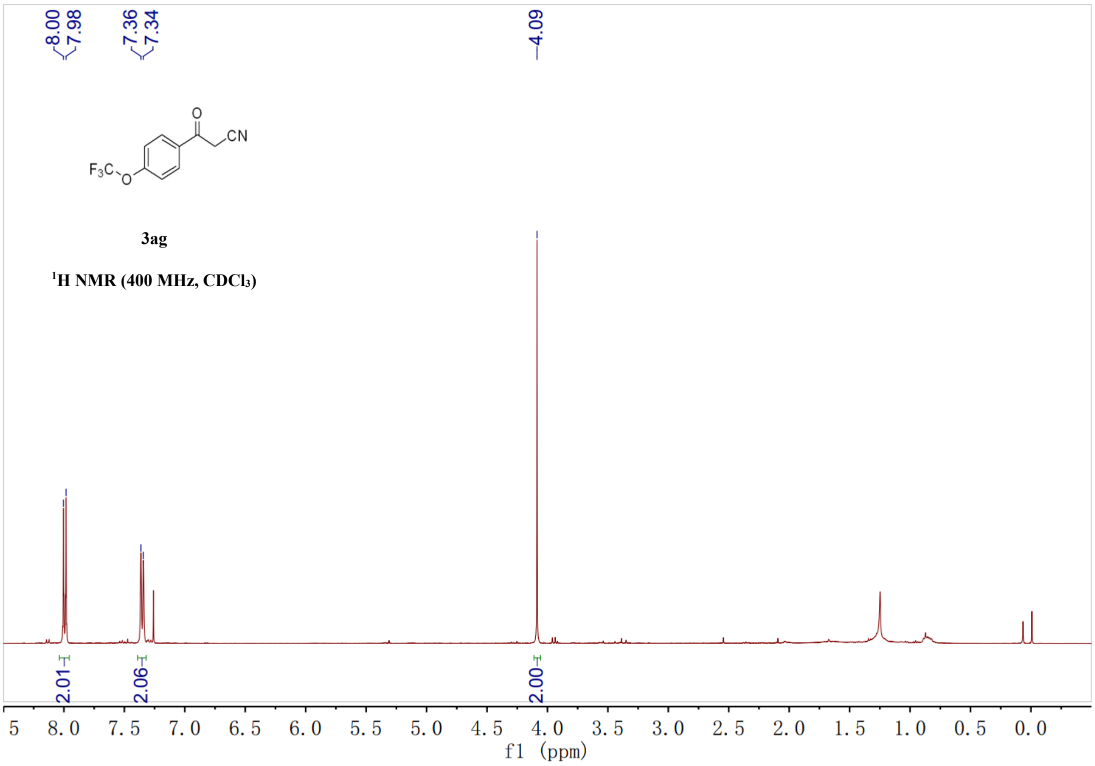
8.00
7.98
7.36
7.34
4.09
O
CN
F3C
O
3ag
1H NMR (400 MHz, CDCl3)
2.01
2.06
2.00
8.0 7.5 7.0 6.5 6.0 5.5 5.0 4.5 4.0 3.5 3.0 2.5 2.0 1.5 1.0 0.5 0.0
f1 (ppm)

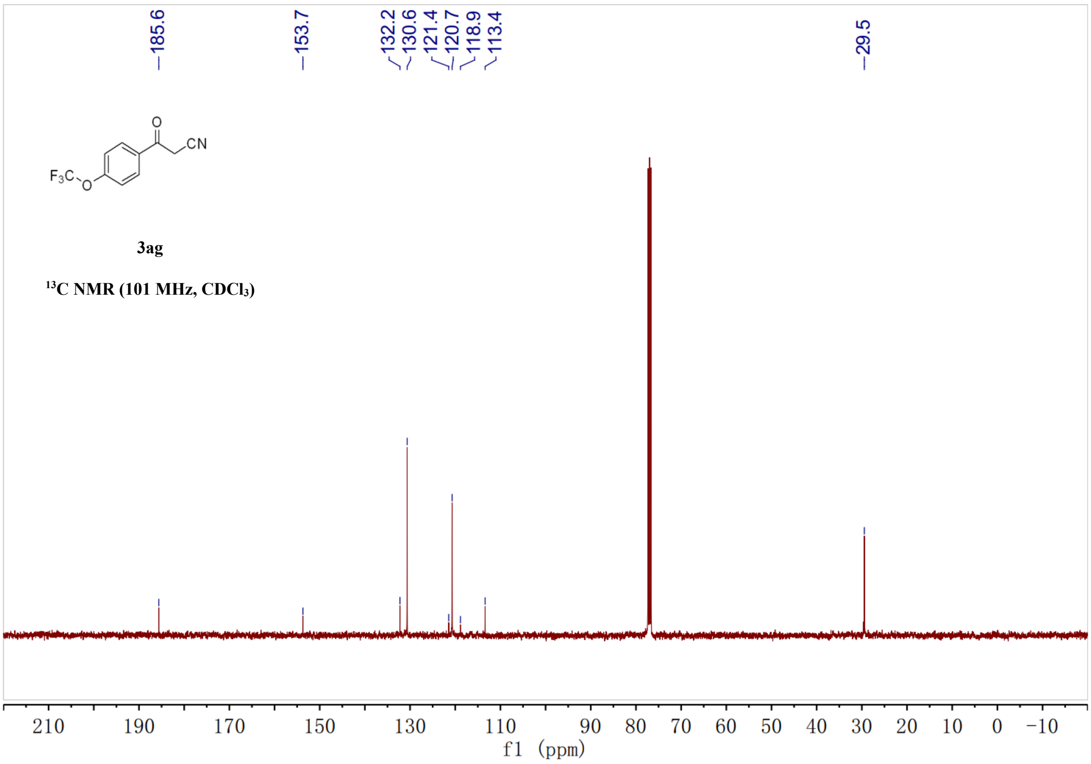
185.6
153.7
132.2
130.6
121.4
120.7
118.9
113.4
29.5
O
CN
F3C
O
3ag
13C NMR (101 MHz, CDCl3)
210 190 170 150 130 110 90 80 70 60 50 40 30 20 10 0 -10
f1 (ppm)

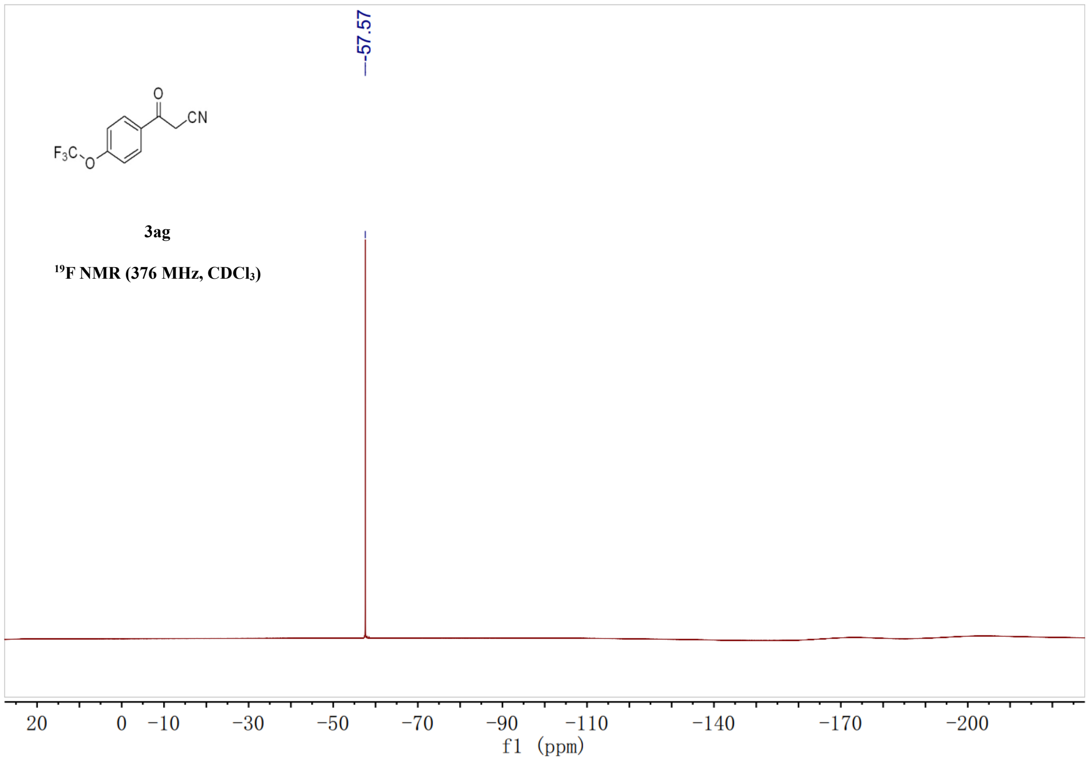
-57.57
3ag
19F NMR (376 MHz, CDCl3)
20
0
-10
-30
-50
-70
-90
-110
-140
-170
-200
f1 (ppm)

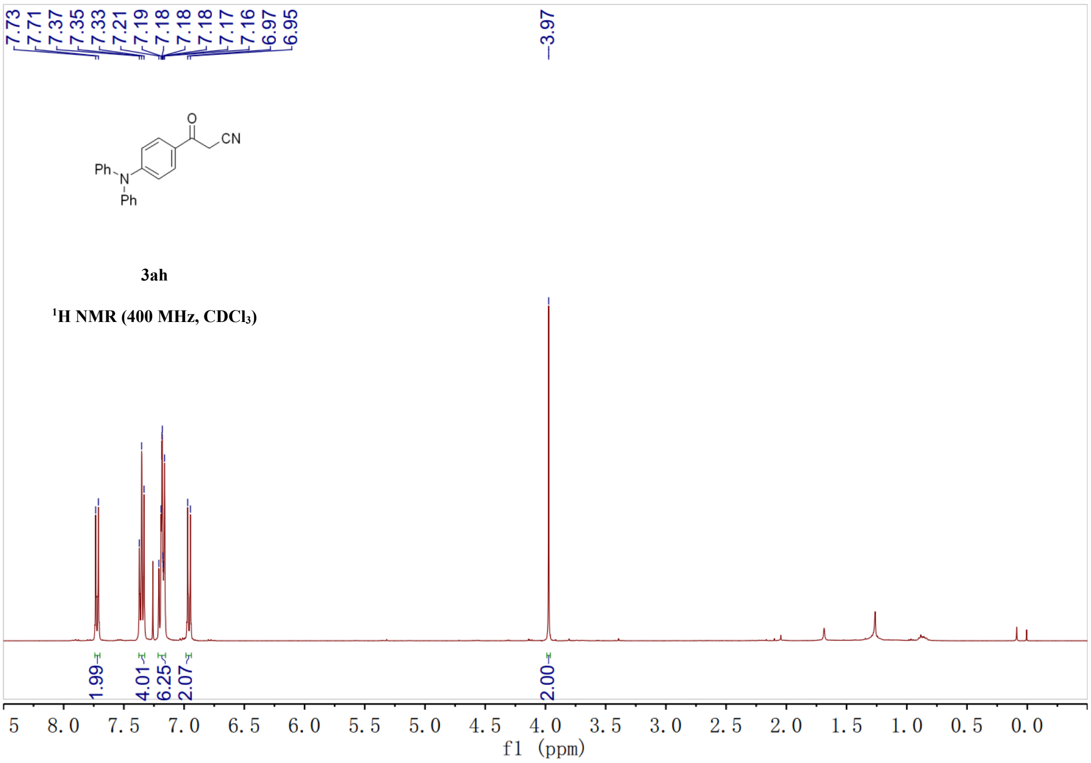

3ah

^{1}H NMR (400 MHz, $CDCl_3$)

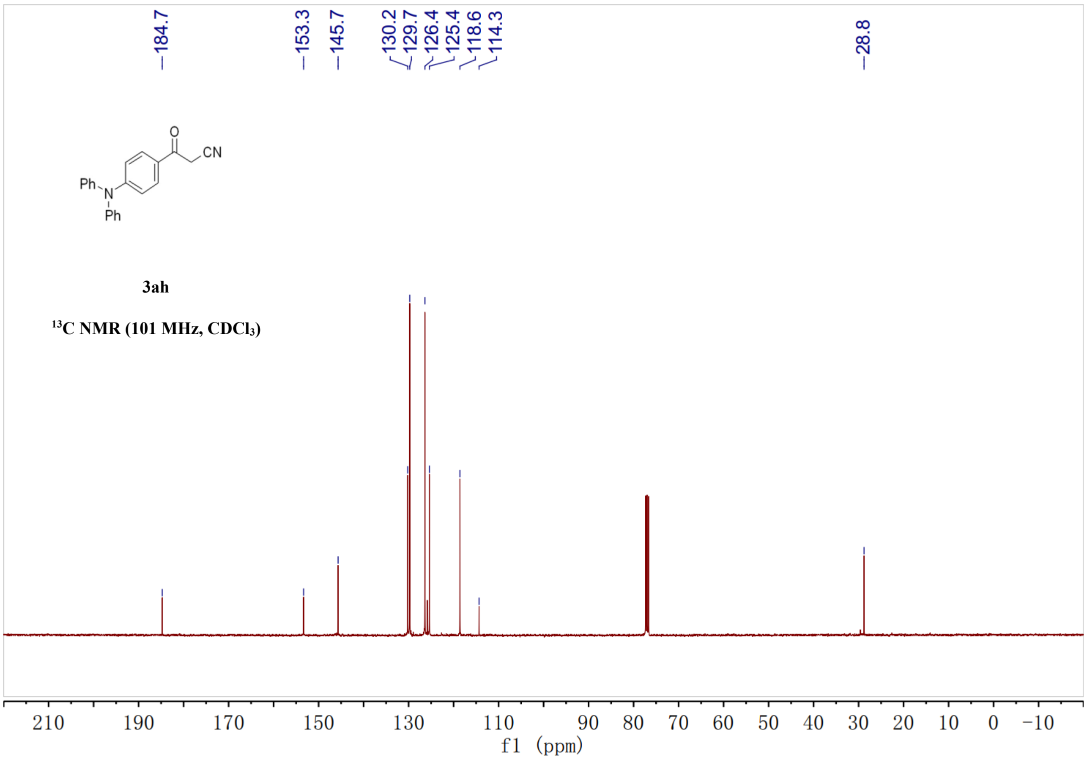

3ah

^{13}C NMR (101 MHz, $CDCl_3$)

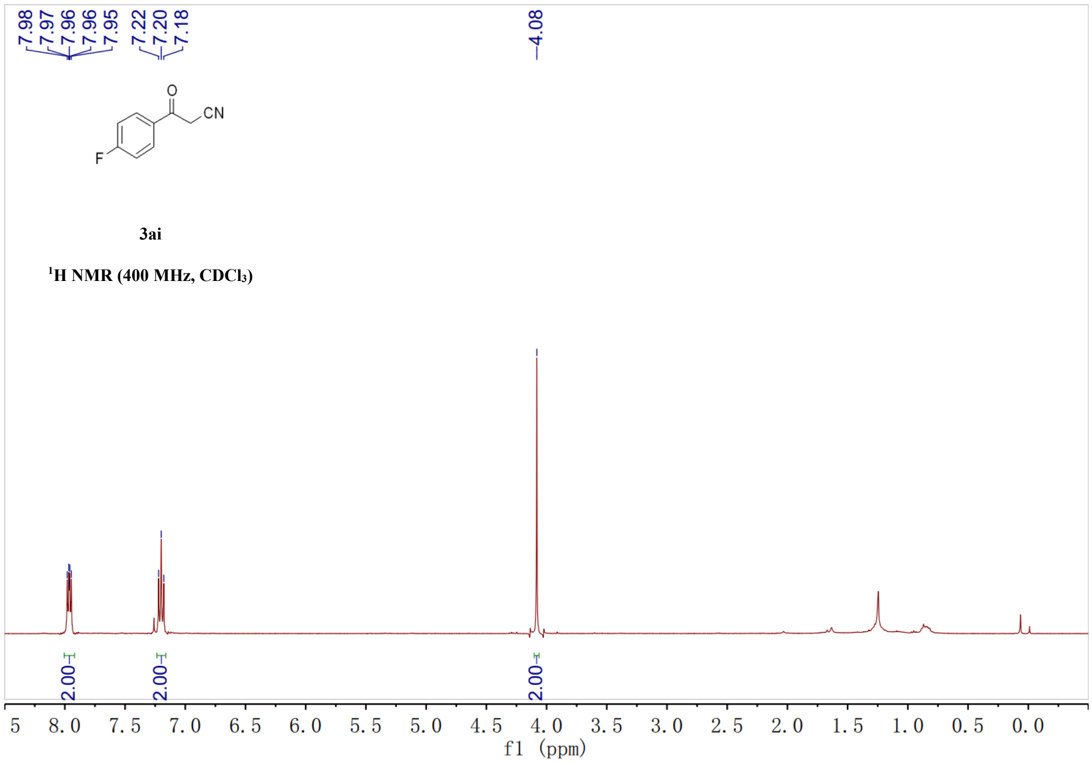
7.98
7.97
7.96
7.96
7.95
7.22
7.20
7.18
4.08
O
CN
F
3ai
^{1}H NMR (400 MHz, CDCl$_3$)
2.00
2.00
2.00
8.0 7.5 7.0 6.5 6.0 5.5 5.0 4.5 4.0 3.5 3.0 2.5 2.0 1.5 1.0 0.5 0.0
f1 (ppm)

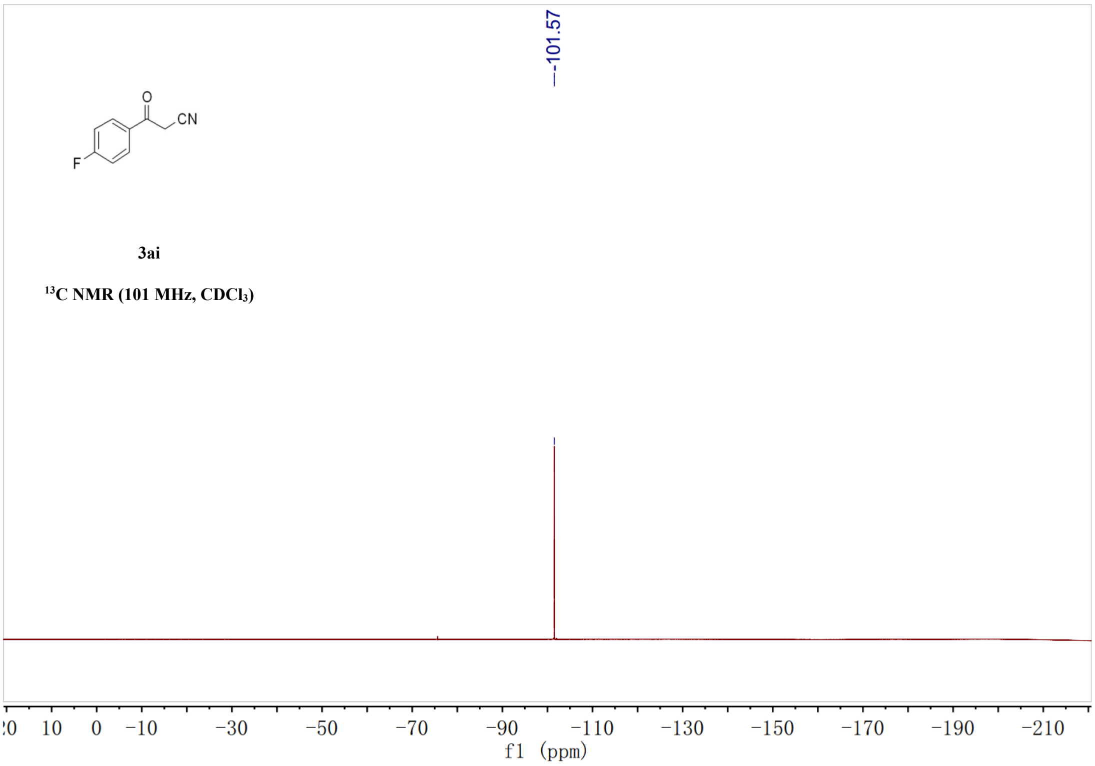
-101.57
O
CN
F
3ai
^{13}C NMR (101 MHz, CDCl$_3$)
10 0 −10 −30 −50 −70 −90 −110 −130 −150 −170 −190 −210
f1 (ppm)

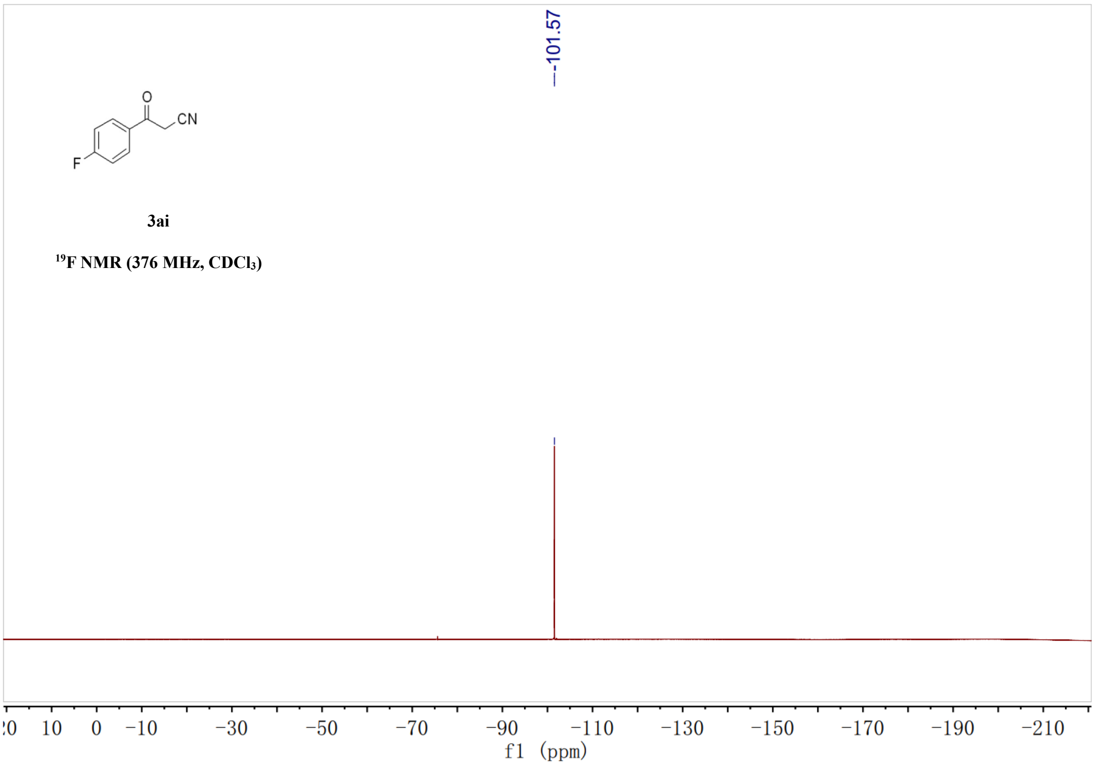

3ai

^{19}F NMR (376 MHz, $CDCl_3$)

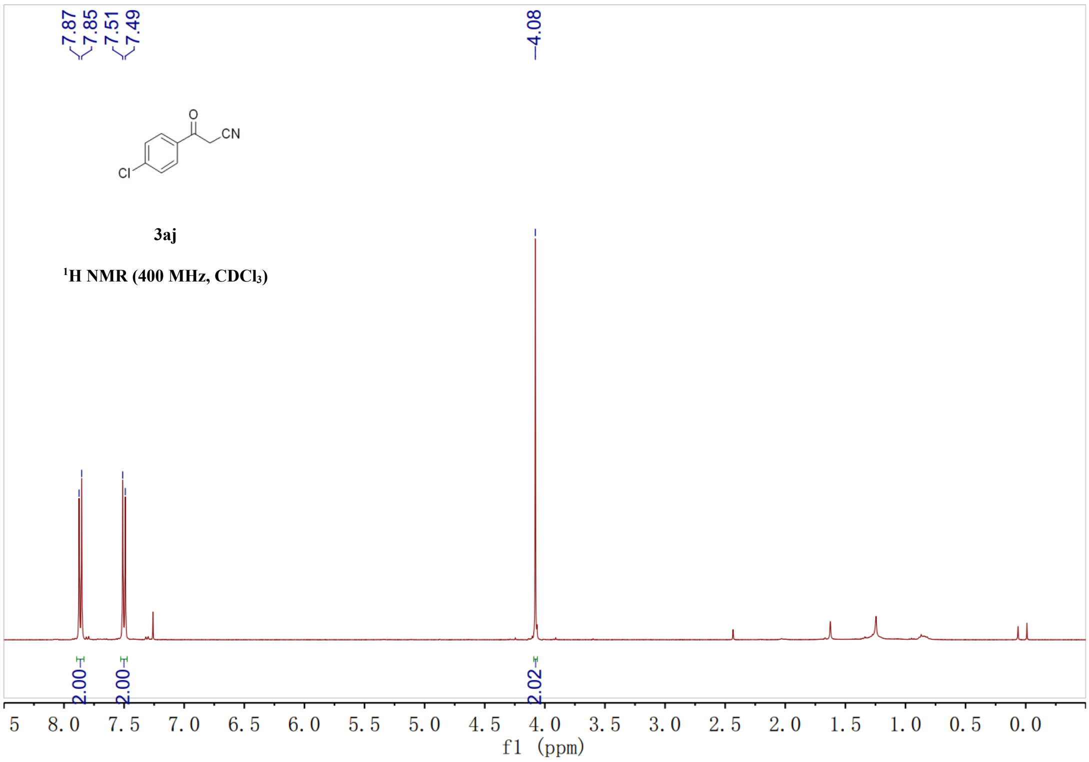
7.87
7.85
7.51
7.49
4.08
O
CN
Cl
3aj
1H NMR (400 MHz, CDCl3)
2.00
2.00
2.02
5 8.0 7.5 7.0 6.5 6.0 5.5 5.0 4.5 4.0 3.5 3.0 2.5 2.0 1.5 1.0 0.5 0.0
f1 (ppm)

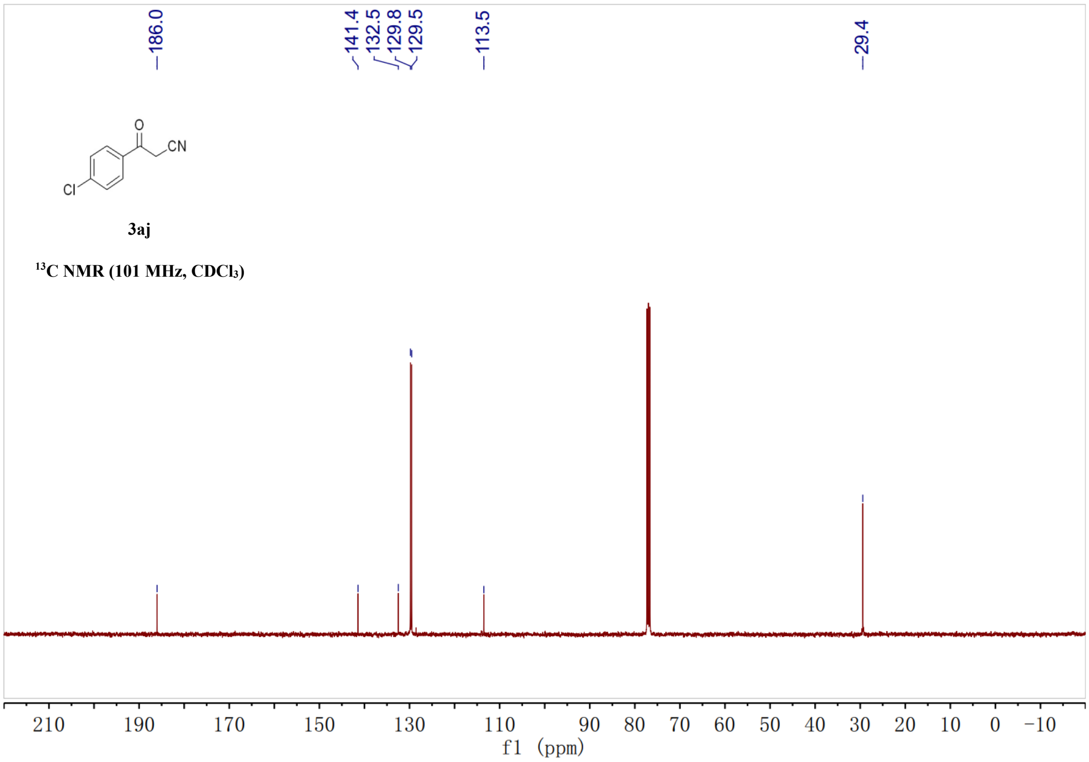
186.0
141.4
132.5
129.8
129.5
113.5
29.4
O
CN
Cl
3aj
13C NMR (101 MHz, CDCl3)
210 190 170 150 130 110 90 80 70 60 50 40 30 20 10 0 -10
f1 (ppm)

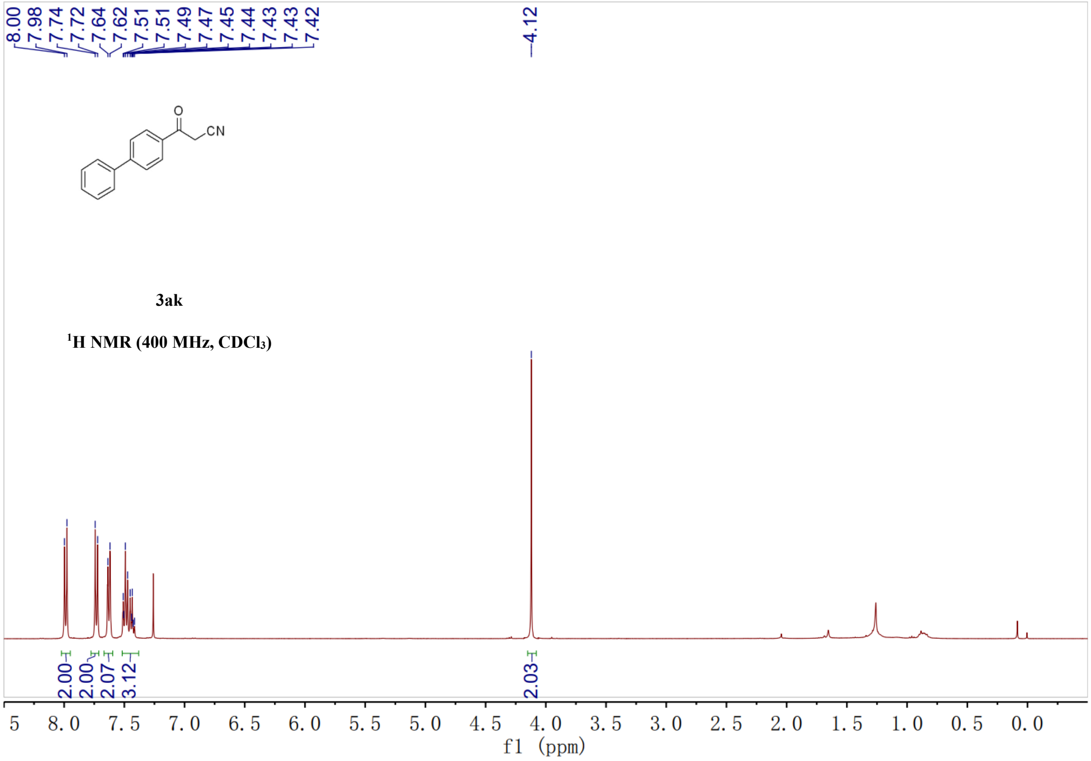
8.00
7.98
7.74
7.72
7.64
7.62
7.51
7.51
7.49
7.47
7.45
7.44
7.43
7.42
4.12
O
CN
3ak
¹H NMR (400 MHz, CDCl₃)
2.00
2.00
2.07
3.12
2.03
5
8.0
7.5
7.0
6.5
6.0
5.5
5.0
4.5
4.0
3.5
3.0
2.5
2.0
1.5
1.0
0.5
0.0
f1 (ppm)

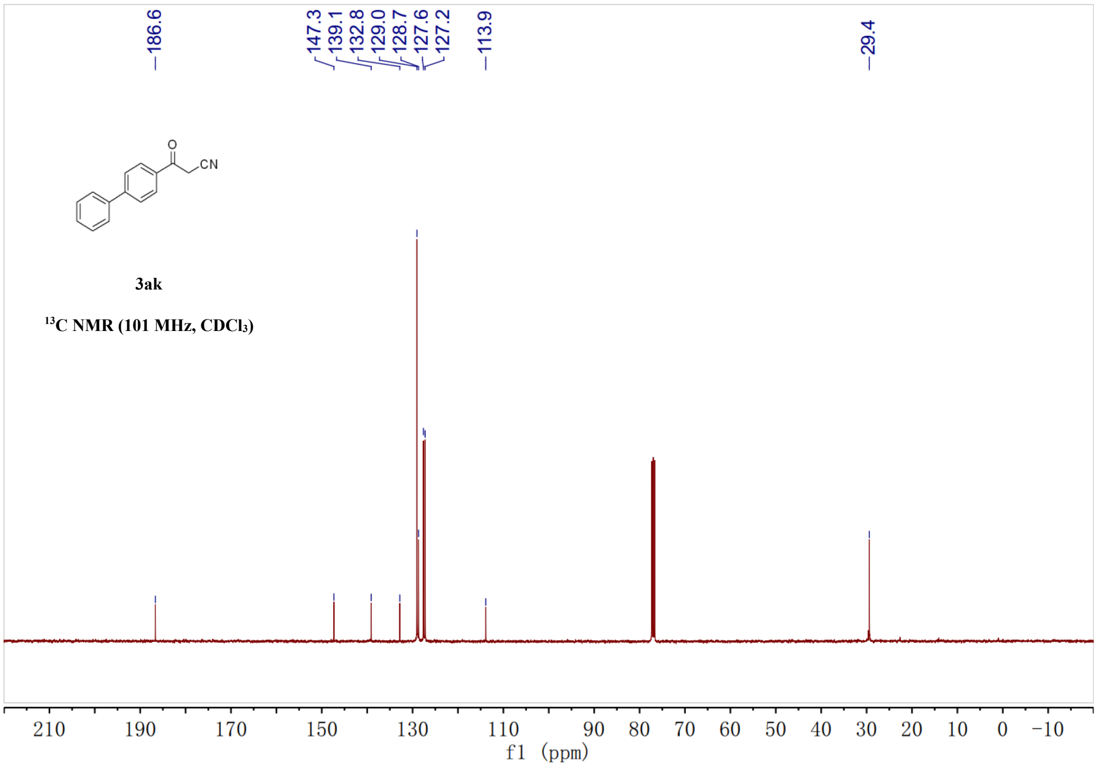
186.6
147.3
139.1
132.8
129.0
128.7
127.6
127.2
113.9
29.4
O
CN
3ak
¹³C NMR (101 MHz, CDCl₃)
210
190
170
150
130
110
90
80
70
60
50
40
30
20
10
0
-10
f1 (ppm)

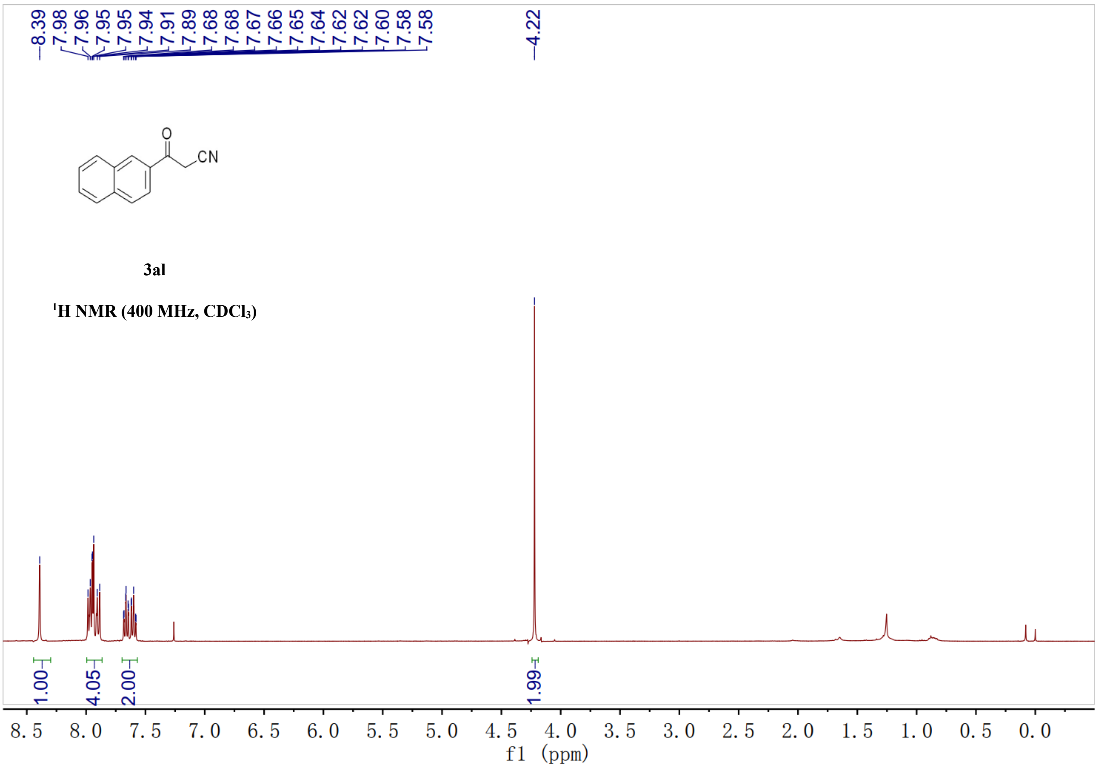
8.39
7.98
7.96
7.95
7.95
7.94
7.91
7.89
7.68
7.68
7.67
7.66
7.65
7.64
7.62
7.62
7.60
7.58
7.58
4.22
O
CN
3al
1H NMR (400 MHz, CDCl3)
1.00
4.05
2.00
1.99
8.5 8.0 7.5 7.0 6.5 6.0 5.5 5.0 4.5 4.0 3.5 3.0 2.5 2.0 1.5 1.0 0.5 0.0
f1 (ppm)

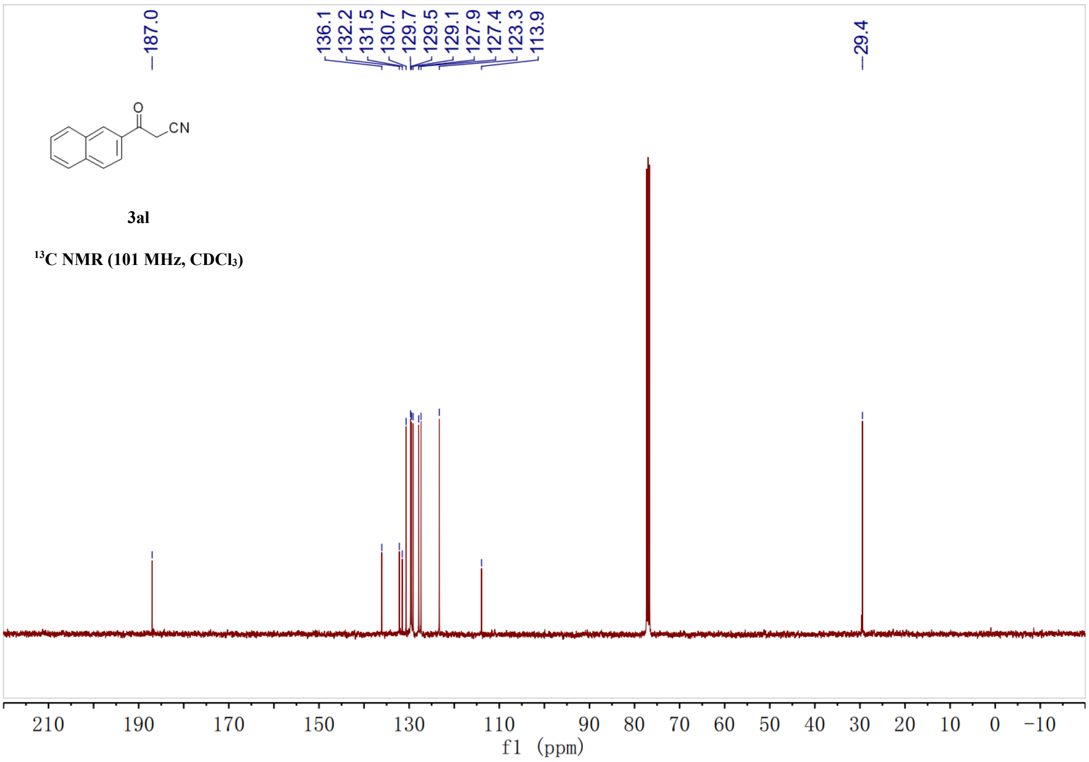
187.0
136.1
132.2
131.5
130.7
129.7
129.5
129.1
127.9
127.4
123.3
113.9
29.4
O
CN
3al
13C NMR (101 MHz, CDCl3)
210 190 170 150 130 110 90 80 70 60 50 40 30 20 10 0 -10
f1 (ppm)

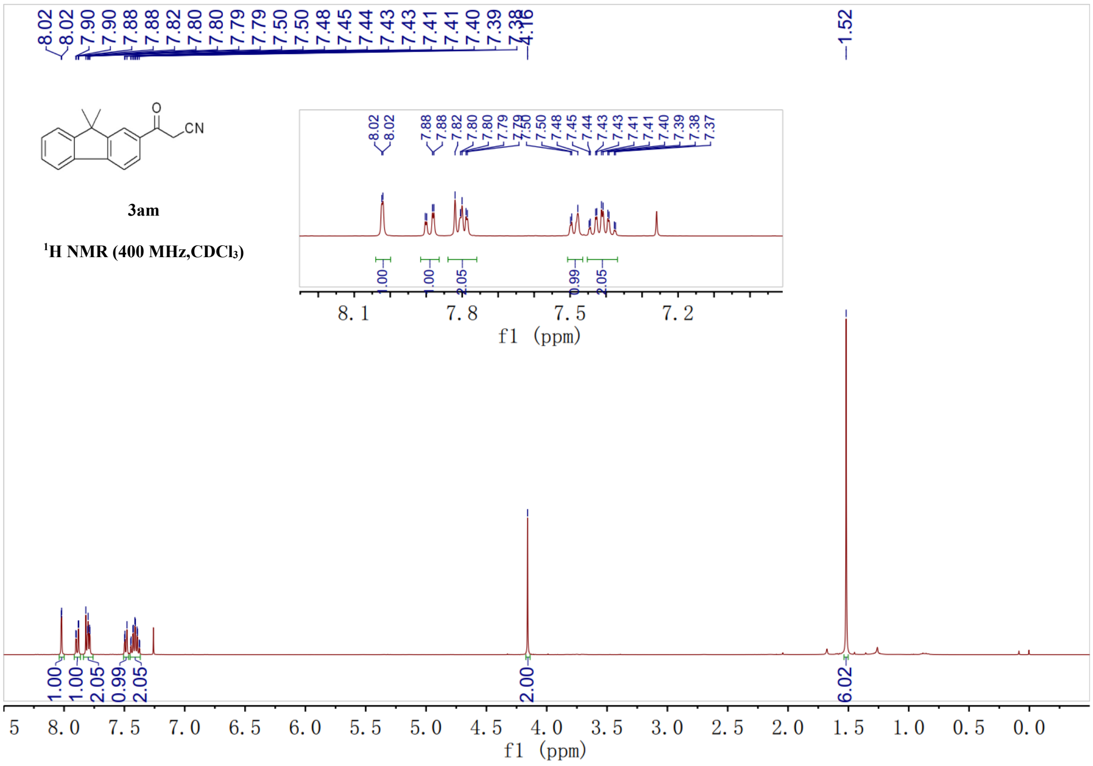

8.02
8.02
7.90
7.90
7.88
7.88
7.82
7.80
7.80
7.79
7.79
7.50
7.50
7.48
7.45
7.44
7.43
7.43
7.41
7.41
7.40
7.39
7.38
4.16
1.52
O
CN
3am
1H NMR (400 MHz,CDCl3)
1.00
1.00
2.05
0.99
2.05
8.1
7.8
7.5
7.2
f1 (ppm)
2.00
6.02
8.0
7.5
7.0
6.5
6.0
5.5
5.0
4.5
4.0
3.5
3.0
2.5
2.0
1.5
1.0
0.5
0.0

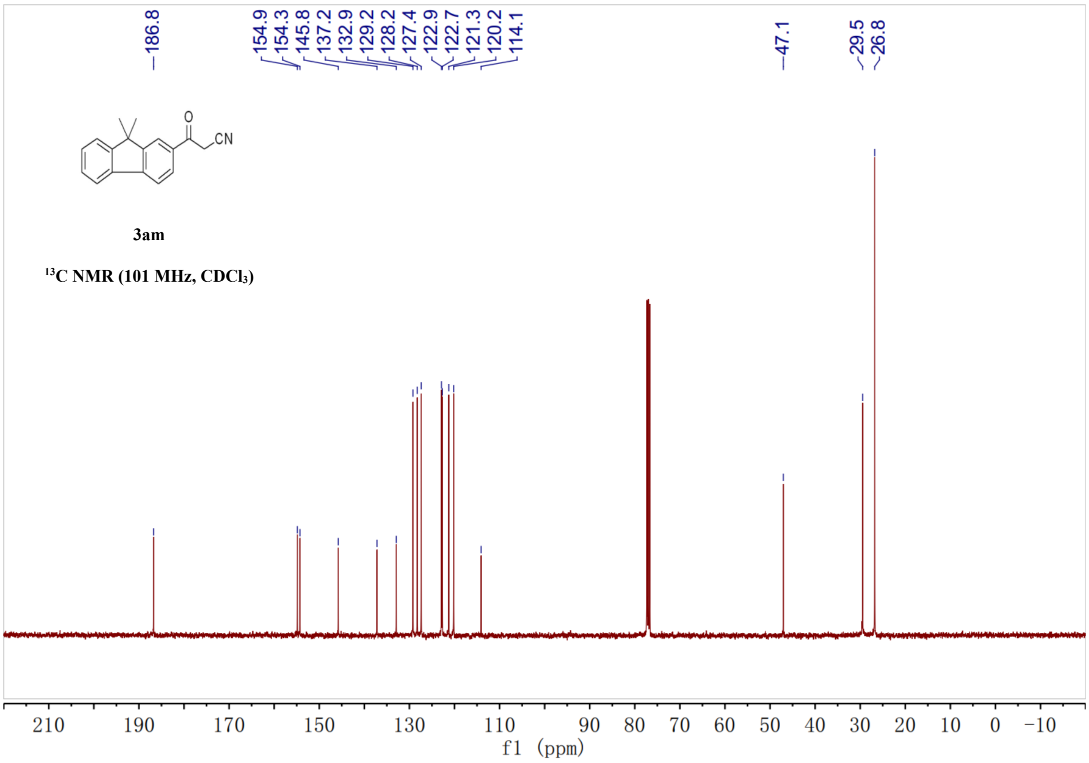

186.8
154.9
154.3
145.8
137.2
132.9
129.2
128.2
127.4
122.9
122.7
121.3
120.2
114.1
47.1
29.5
26.8
O
CN
3am
13C NMR (101 MHz, CDCl3)
210
190
170
150
130
110
90
80
70
60
50
40
30
20
10
0
-10
f1 (ppm)

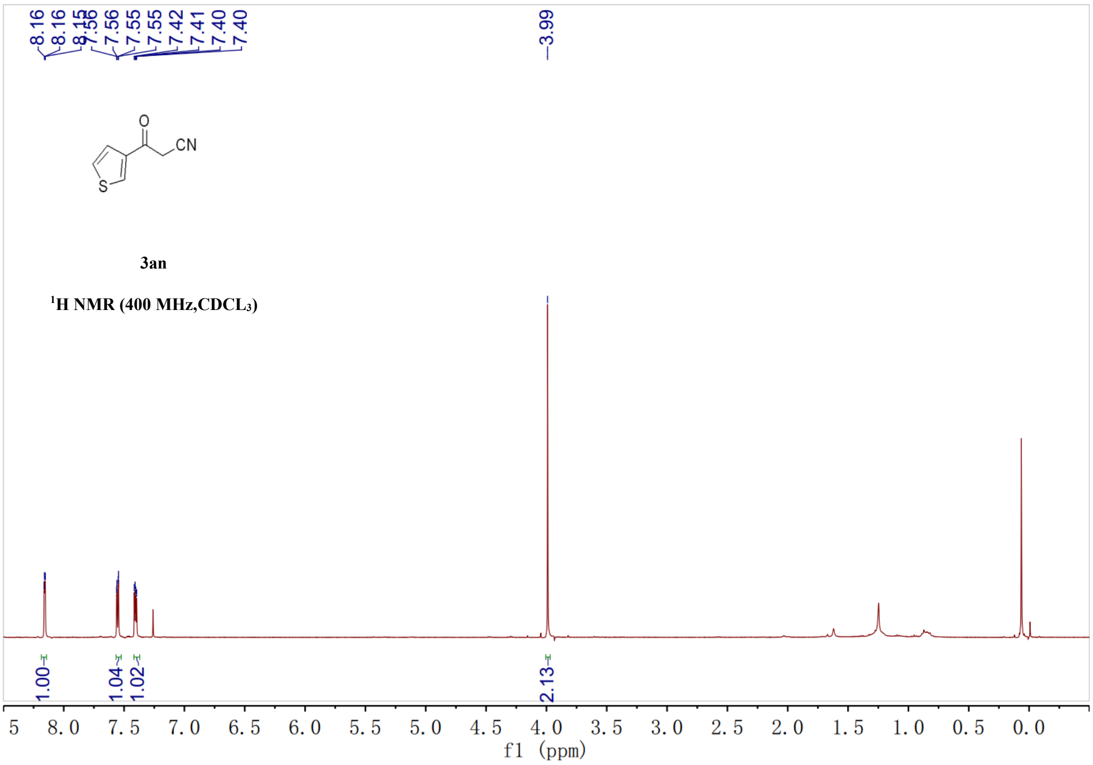
8.16
8.16
8.15
7.56
7.56
7.55
7.55
7.42
7.41
7.40
7.40
3.99
O
CN
S
3an
1H NMR (400 MHz,CDCL3)
1.00
1.04
1.02
2.13
5
8.0
7.5
7.0
6.5
6.0
5.5
5.0
4.5
4.0
3.5
3.0
2.5
2.0
1.5
1.0
0.5
0.0
f1 (ppm)

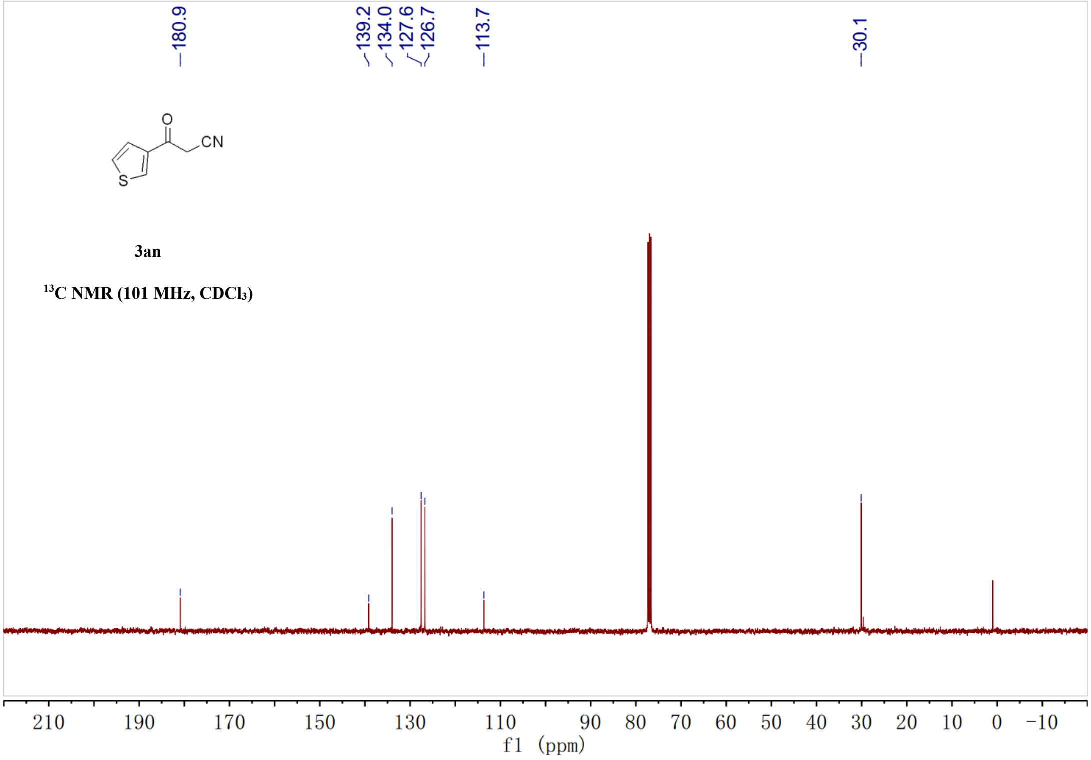
180.9
139.2
134.0
127.6
126.7
113.7
30.1
O
CN
S
3an
13C NMR (101 MHz, CDCl3)
210
190
170
150
130
110
90
80
70
60
50
40
30
20
10
0
−10
f1 (ppm)

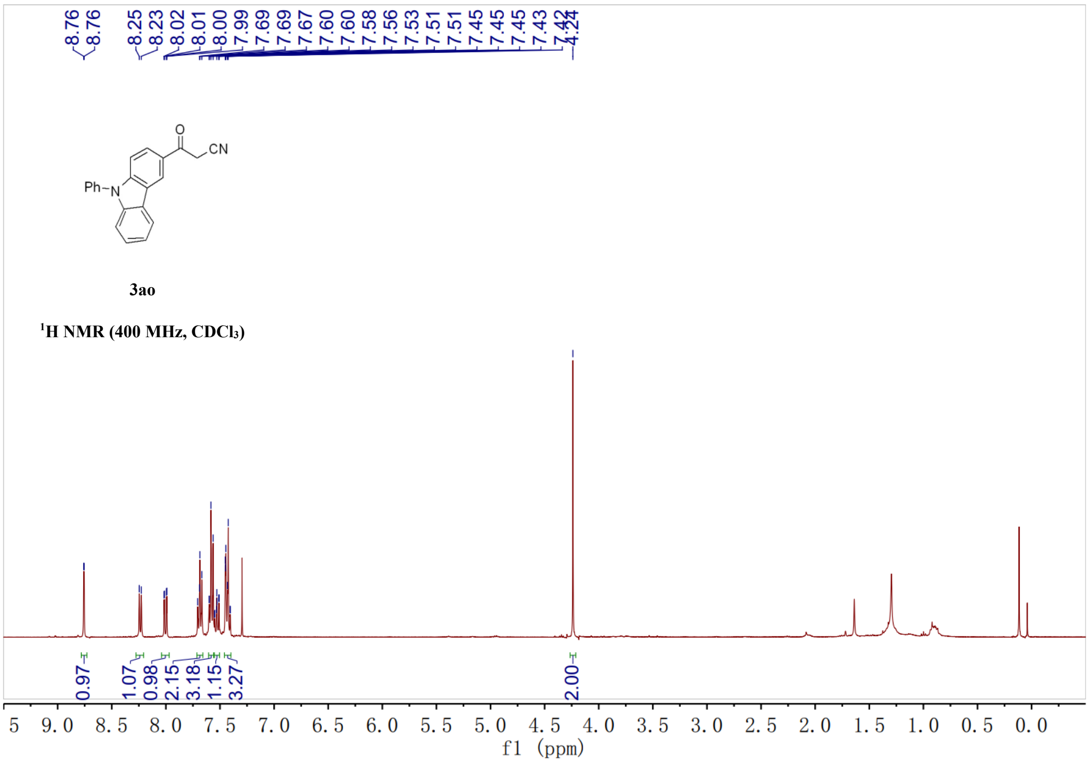
8.76
8.76
8.25
8.23
8.02
8.01
8.00
7.99
7.69
7.67
7.60
7.60
7.58
7.56
7.53
7.51
7.51
7.45
7.45
7.45
7.43
7.42
4.24
O
CN
Ph
N
3ao
1H NMR (400 MHz, CDCl3)
0.97
1.07
0.98
2.15
3.18
1.15
3.27
2.00
5
9.0
8.5
8.0
7.5
7.0
6.5
6.0
5.5
5.0
4.5
4.0
3.5
3.0
2.5
2.0
1.5
1.0
0.5
0.0
f1 (ppm)

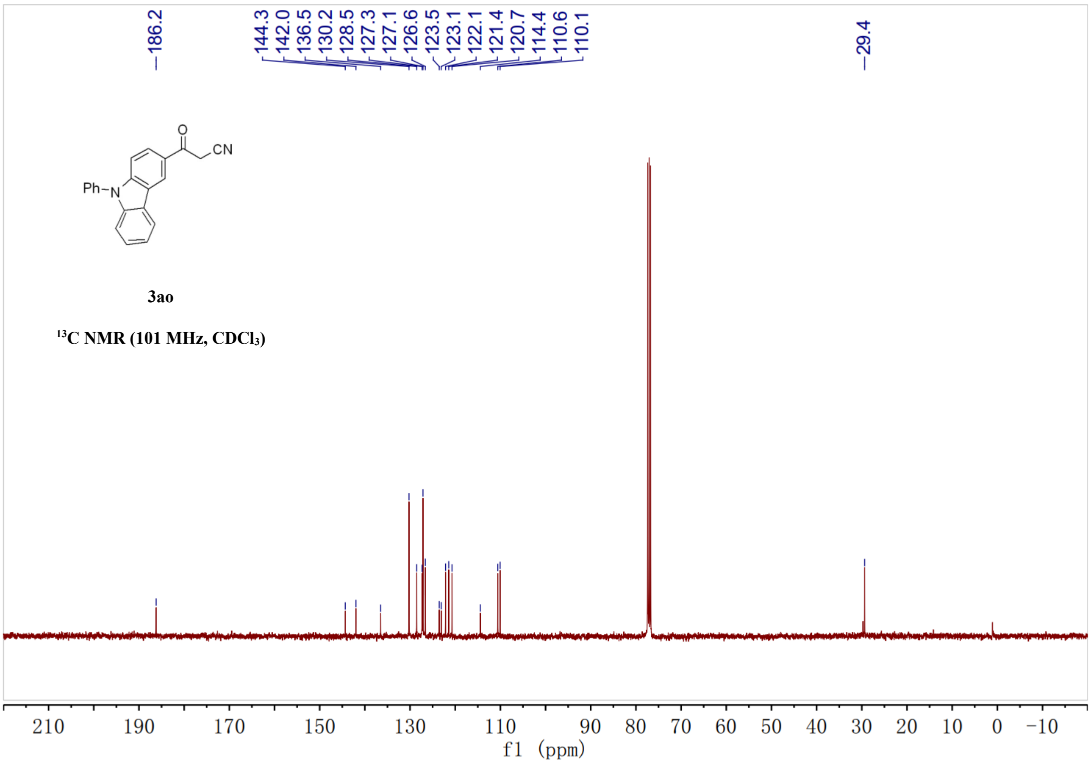
186.2
144.3
142.0
136.5
130.2
128.5
127.3
127.1
126.6
123.5
123.1
122.1
121.4
120.7
114.4
110.6
110.1
29.4
O
CN
Ph
N
3ao
13C NMR (101 MHz, CDCl3)
210
190
170
150
130
110
90
80
70
60
50
40
30
20
10
0
−10
f1 (ppm)

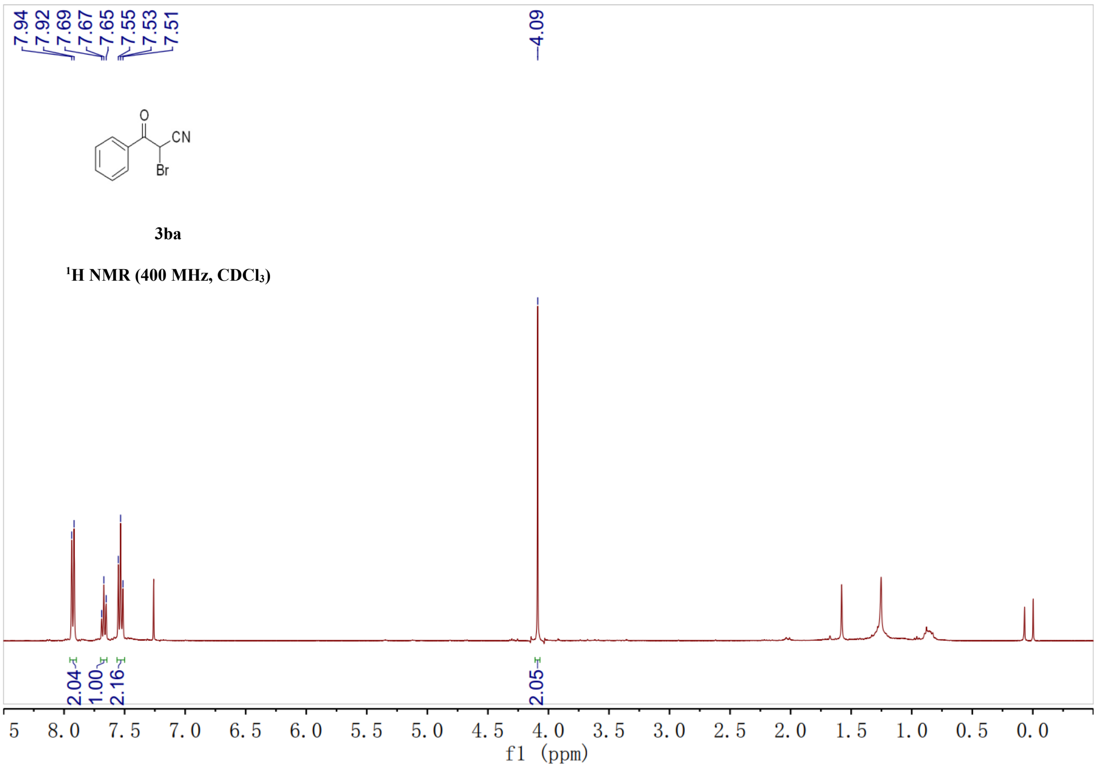
7.94
7.92
7.69
7.67
7.65
7.55
7.53
7.51
4.09
O
CN
Br
3ba
1H NMR (400 MHz, CDCl3)
2.04
1.00
2.16
2.05
f1 (ppm)

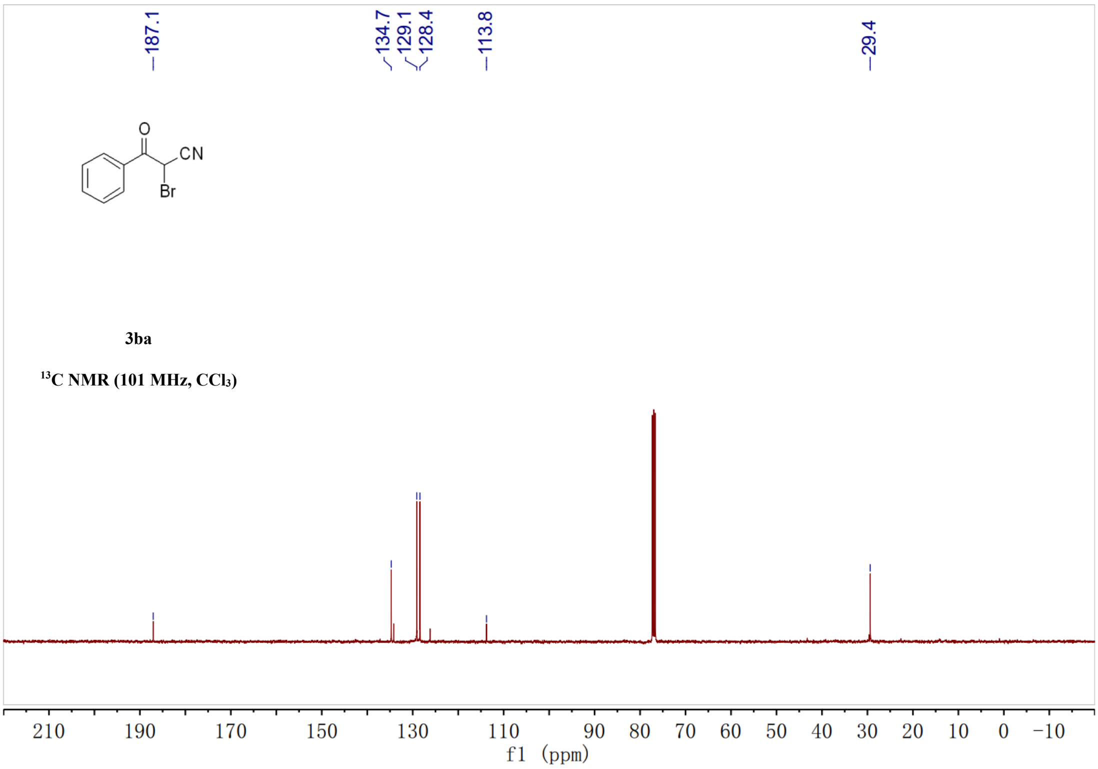
187.1
134.7
129.1
128.4
113.8
29.4
O
CN
Br
3ba
13C NMR (101 MHz, CCl3)
f1 (ppm)

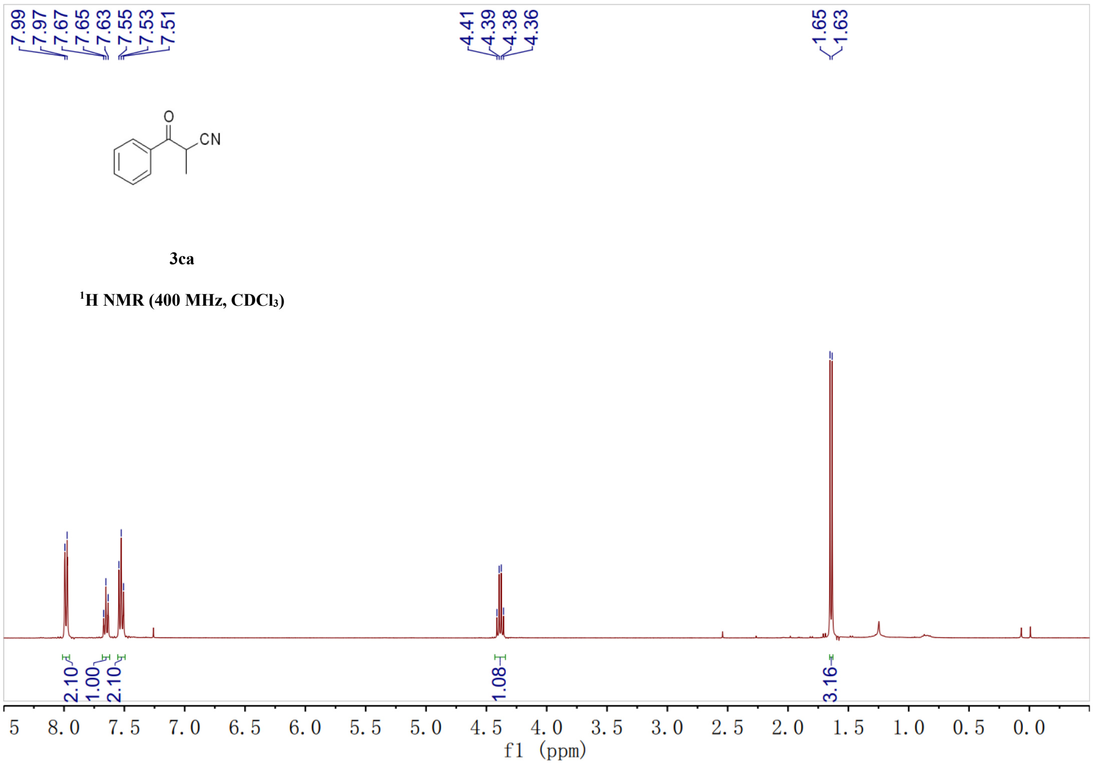
7.99
7.97
7.67
7.65
7.63
7.55
7.53
7.51
4.41
4.39
4.38
4.36
1.65
1.63
O
CN
3ca
1H NMR (400 MHz, CDCl3)
2.10
1.00
2.10
1.08
3.16
f1 (ppm)

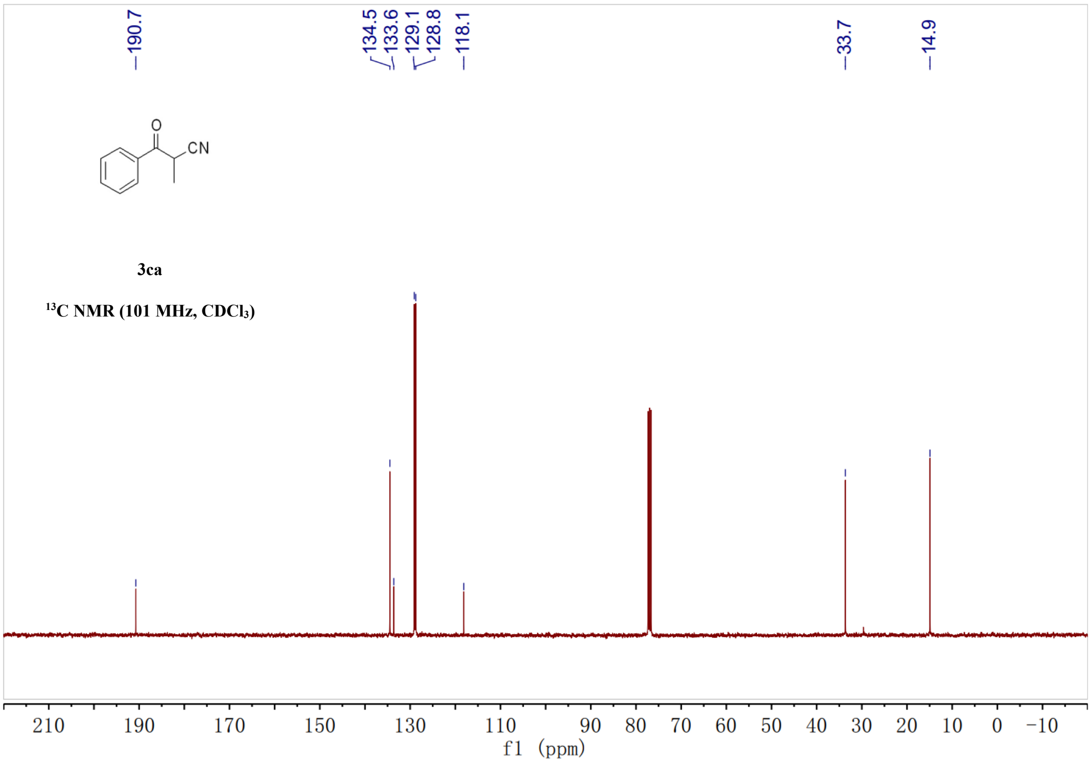
190.7
134.5
133.6
129.1
128.8
118.1
33.7
14.9
O
CN
3ca
13C NMR (101 MHz, CDCl3)
f1 (ppm)

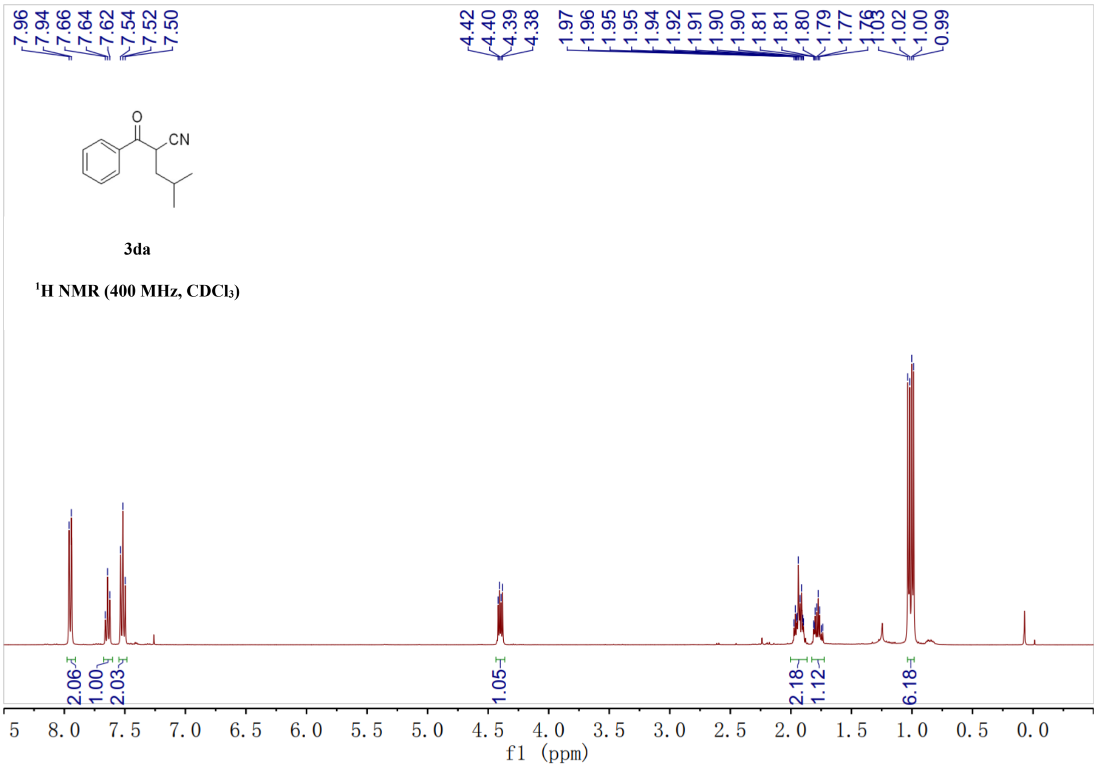
7.96 7.94 7.66 7.64 7.62 7.54 7.52 7.50
4.42 4.40 4.39 4.38
1.97 1.96 1.95 1.95 1.94 1.92 1.91 1.90 1.90 1.81 1.81 1.80 1.79 1.77 1.76 1.03 1.02 1.00 0.99
O
CN
3da
1H NMR (400 MHz, CDCl3)
2.06 1.00 2.03 1.05 2.18 1.12 6.18
5 8.0 7.5 7.0 6.5 6.0 5.5 5.0 4.5 4.0 3.5 3.0 2.5 2.0 1.5 1.0 0.5 0.0
f1 (ppm)

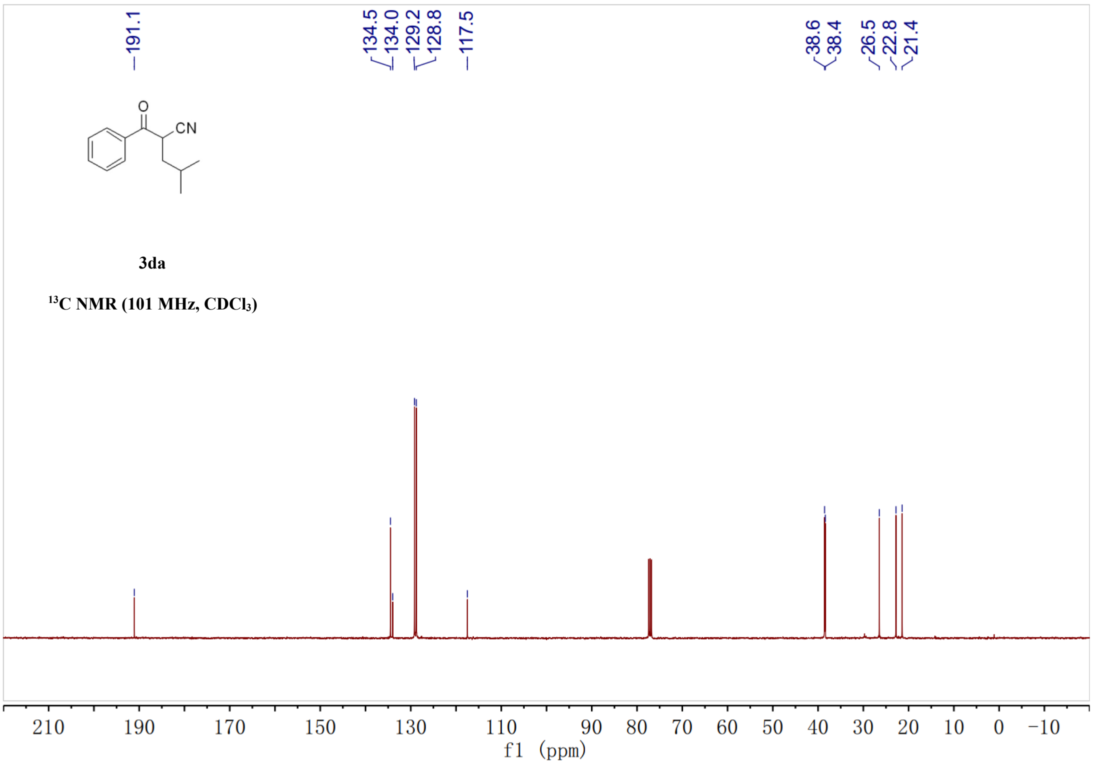
191.1
134.5 134.0 129.2 128.8 117.5
38.6 38.4 26.5 22.8 21.4
O
CN
3da
13C NMR (101 MHz, CDCl3)
210 190 170 150 130 110 90 80 70 60 50 40 30 20 10 0 −10
f1 (ppm)

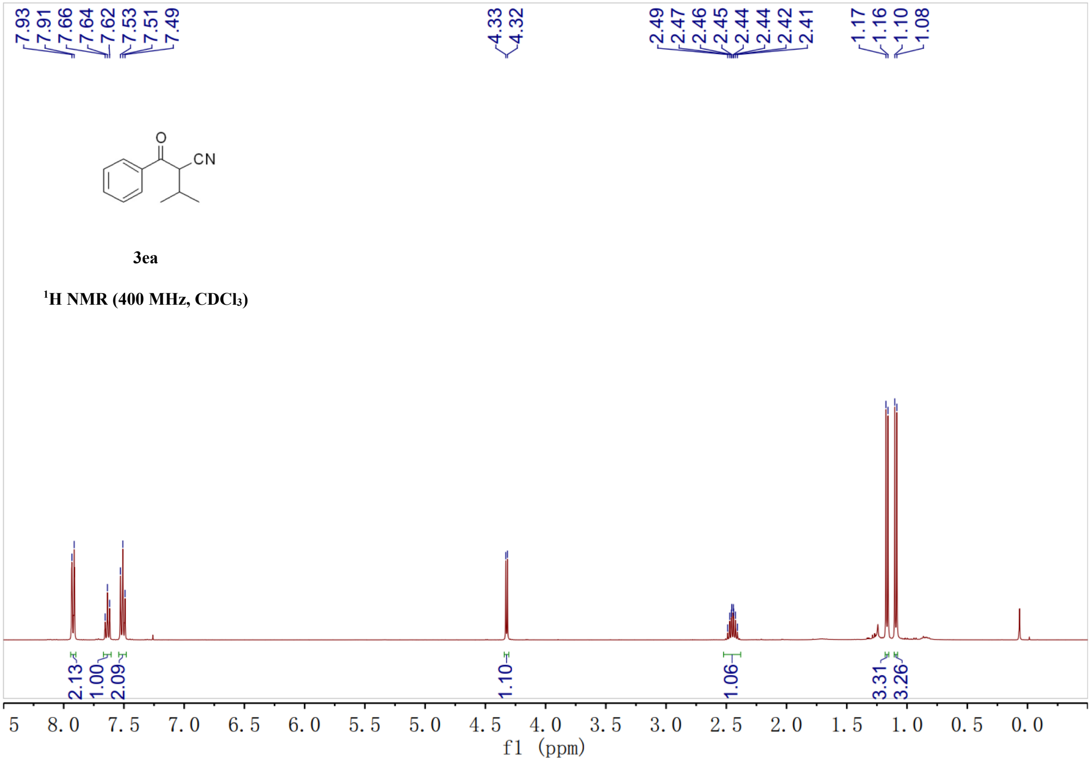
7.93
7.91
7.66
7.64
7.62
7.53
7.51
7.49
4.33
4.32
2.49
2.47
2.46
2.45
2.44
2.44
2.42
2.41
1.17
1.16
1.10
1.08
CN
O
3ea
1H NMR (400 MHz, CDCl3)
2.13
1.00
2.09
1.10
1.06
3.31
3.26
5 8.0 7.5 7.0 6.5 6.0 5.5 5.0 4.5 4.0 3.5 3.0 2.5 2.0 1.5 1.0 0.5 0.0
f1 (ppm)

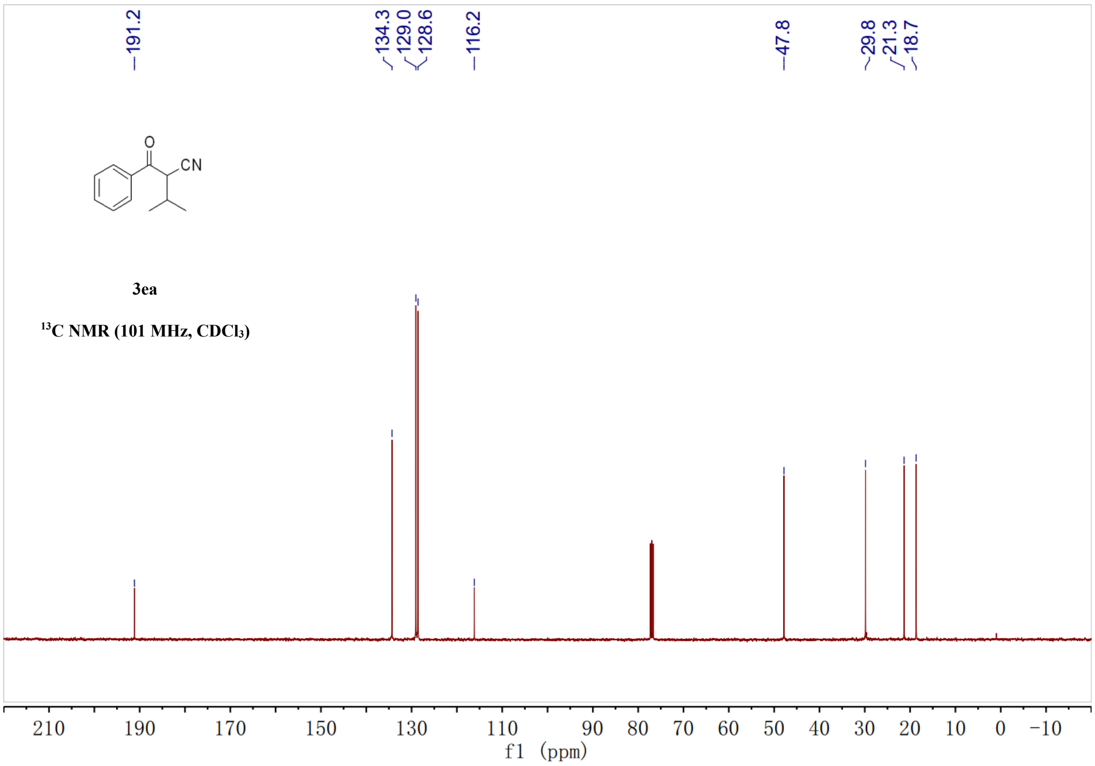
191.2
134.3
129.0
128.6
116.2
47.8
29.8
21.3
18.7
CN
O
3ea
13C NMR (101 MHz, CDCl3)
210 190 170 150 130 110 90 80 70 60 50 40 30 20 10 0 -10
f1 (ppm)

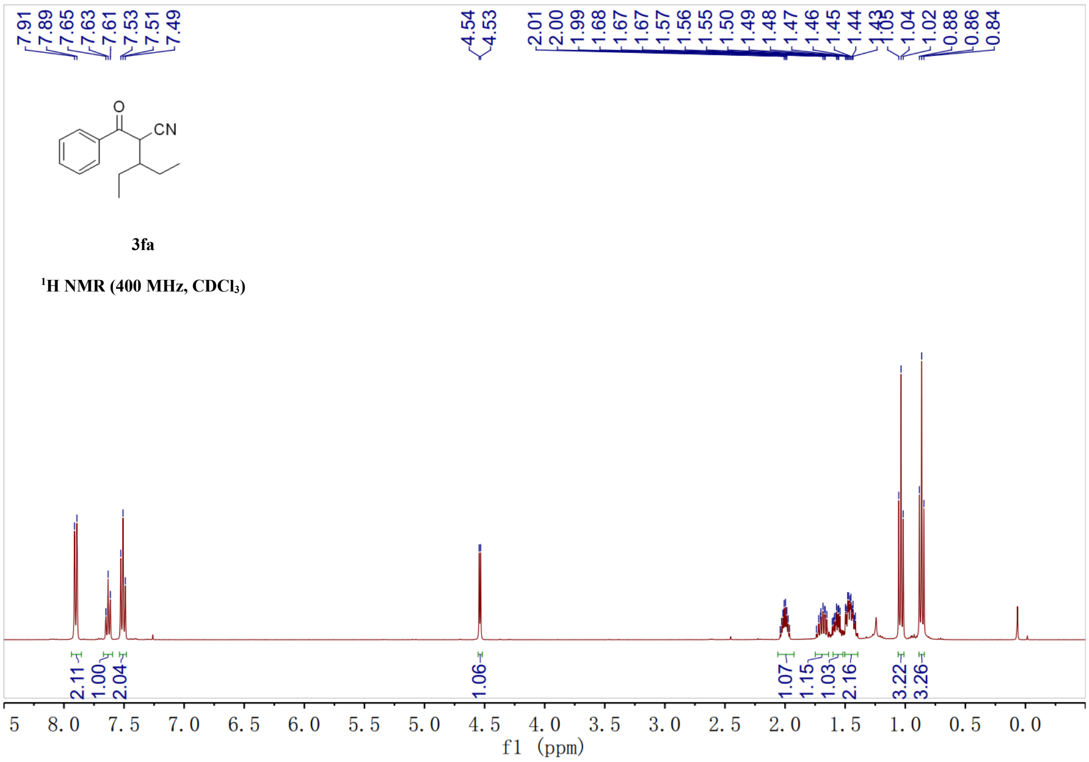

7.91 7.89 7.65 7.63 7.61 7.53 7.51 7.49
4.54 4.53
2.01 2.00 1.99 1.68 1.67 1.57 1.56 1.55 1.50 1.49 1.48 1.47 1.46 1.45 1.44 1.43 1.05 1.04 1.02 0.88 0.86 0.84
O
CN
3fa
^{1}H NMR (400 MHz, CDCl$_3$)
2.11 1.00 2.04
1.06
1.07 1.15 1.03 2.16
3.22 3.26
f1 (ppm)

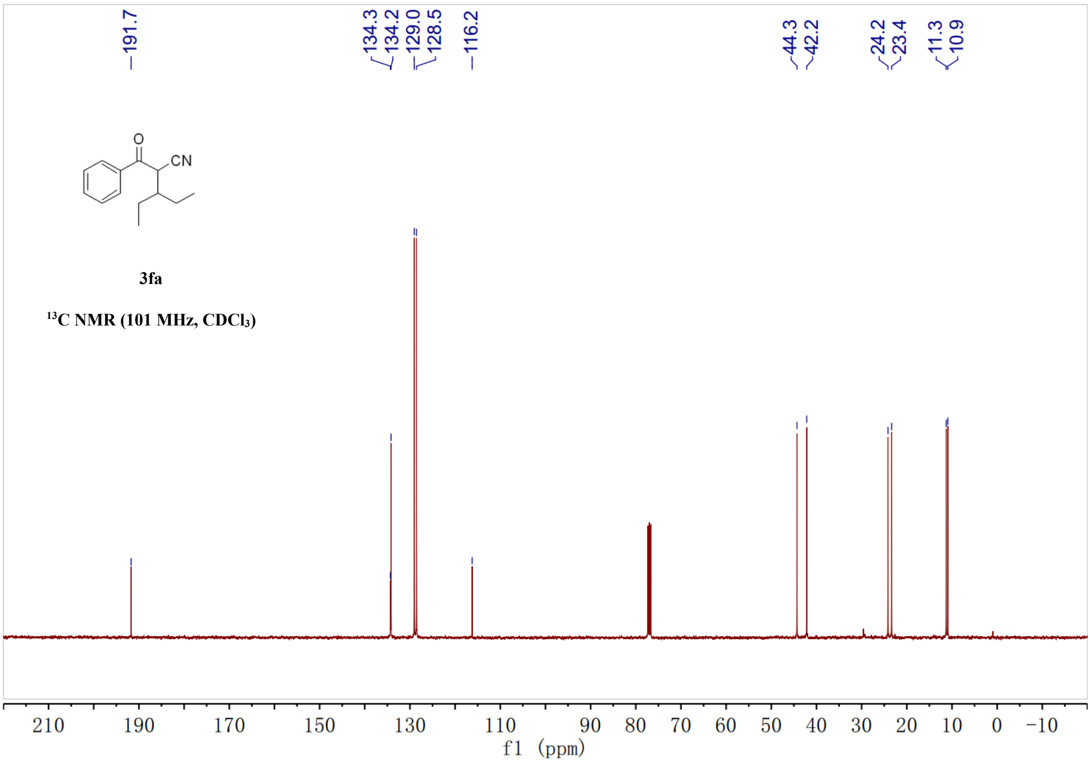

191.7
134.3 134.2 129.0 128.5
116.2
44.3 42.2
24.2 23.4
11.3 10.9
O
CN
3fa
^{13}C NMR (101 MHz, CDCl$_3$)
f1 (ppm)

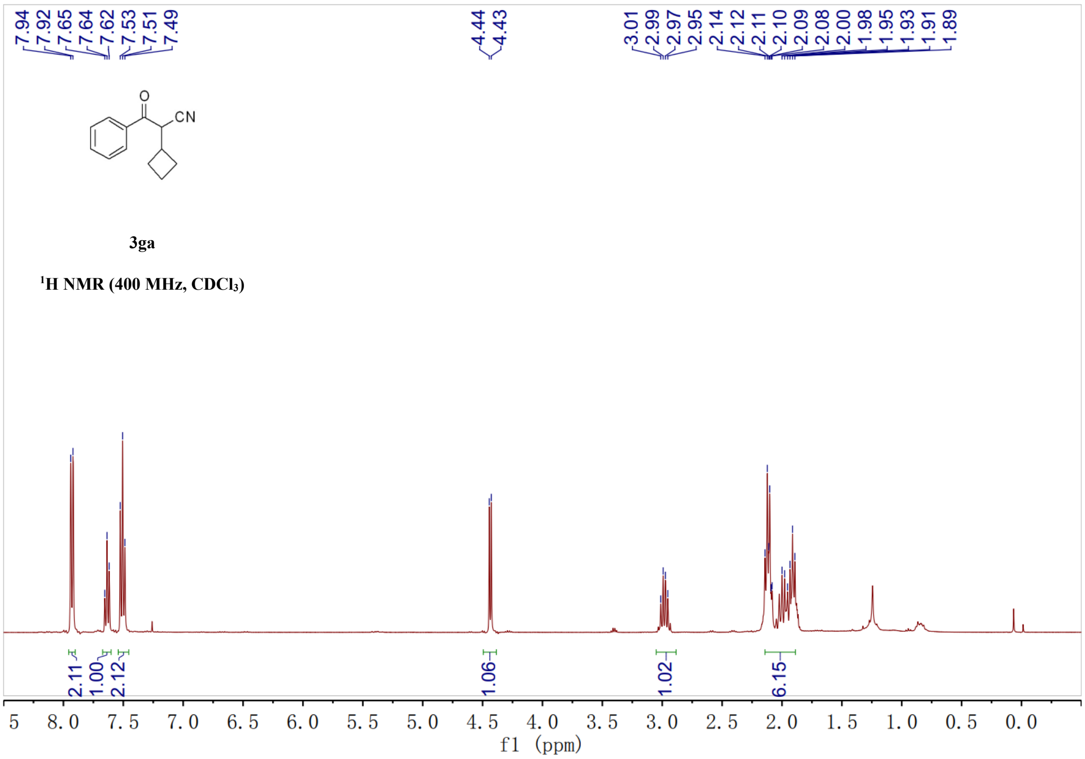
7.94
7.92
7.65
7.64
7.62
7.53
7.51
7.49
4.44
4.43
3.01
2.99
2.97
2.95
2.14
2.12
2.11
2.10
2.09
2.08
2.00
1.98
1.95
1.93
1.91
1.89
O
CN
3ga
¹H NMR (400 MHz, CDCl₃)
2.11
1.00
2.12
1.06
1.02
6.15
f1 (ppm)

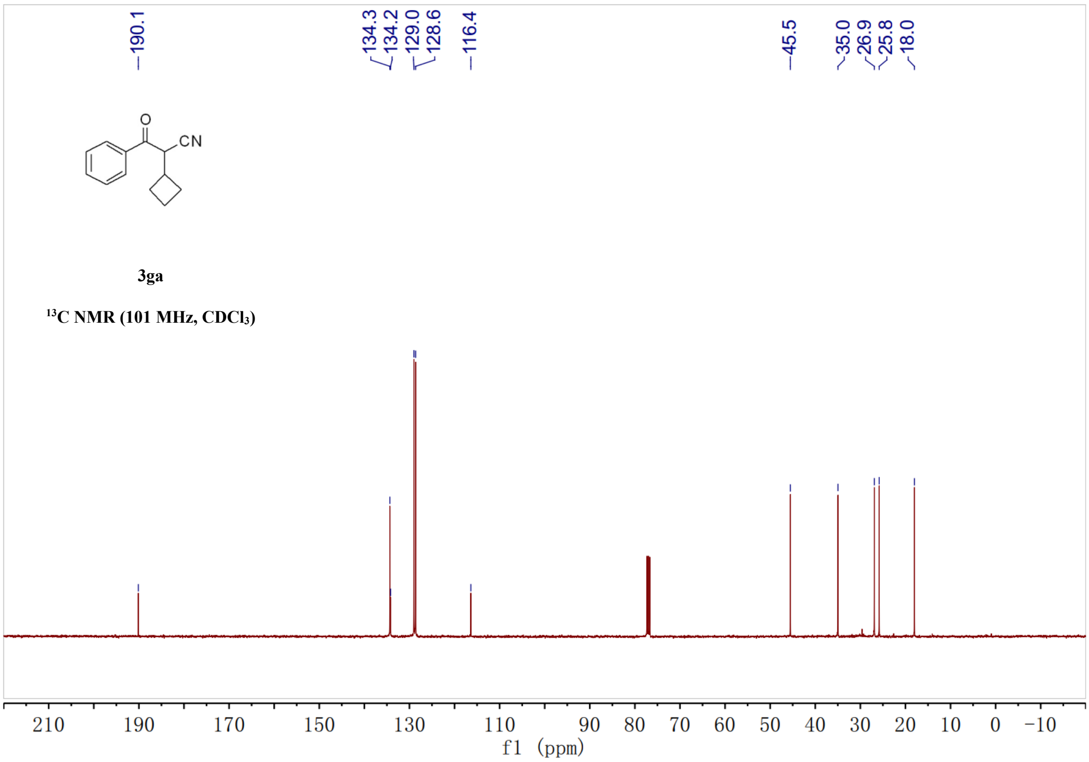
190.1
134.3
134.2
129.0
128.6
116.4
45.5
35.0
26.9
25.8
18.0
O
CN
3ga
¹³C NMR (101 MHz, CDCl₃)
f1 (ppm)

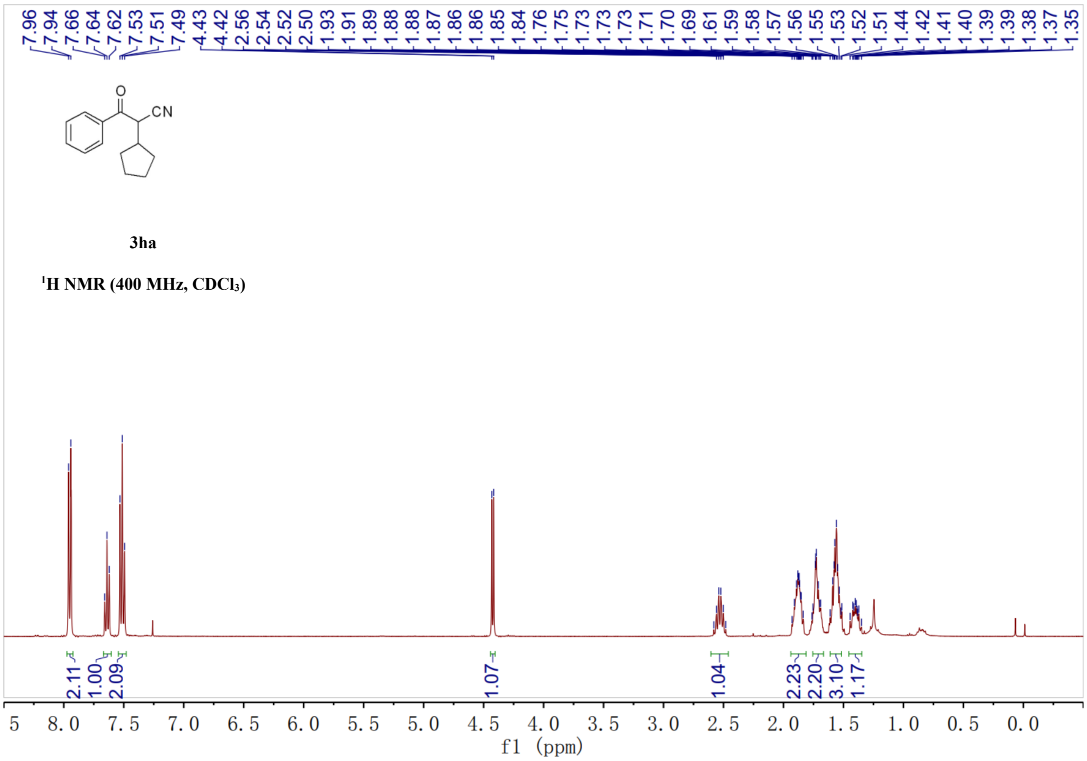
7.96 7.94 7.66 7.64 7.62 7.53 7.51 7.49 4.43 4.42 2.56 2.54 2.52 2.50 1.93 1.91 1.89 1.88 1.88 1.87 1.86 1.86 1.85 1.84 1.76 1.75 1.73 1.73 1.73 1.71 1.70 1.69 1.61 1.59 1.58 1.57 1.56 1.55 1.53 1.52 1.51 1.44 1.42 1.41 1.40 1.39 1.38 1.37 1.35
O
CN
3ha
¹H NMR (400 MHz, CDCl₃)
2.11 1.00 2.09 1.07 1.04 2.23 2.20 3.10 1.17
f1 (ppm)

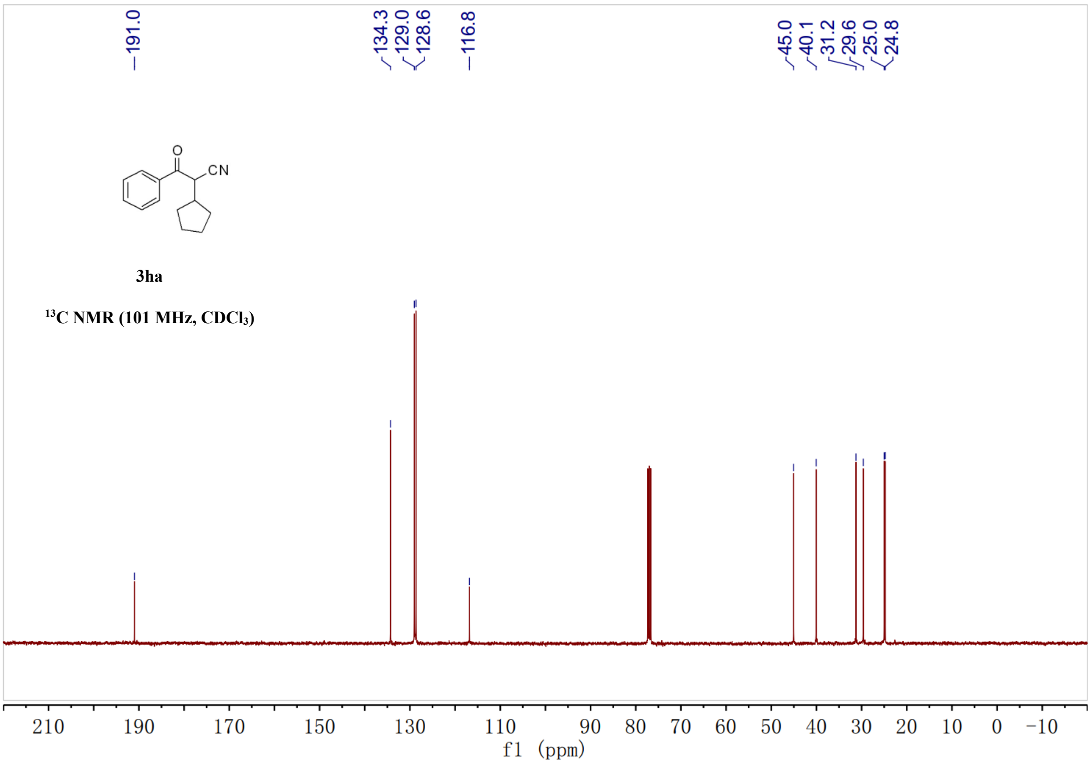
191.0 134.3 129.0 128.6 116.8 45.0 40.1 31.2 29.6 25.0 24.8
O
CN
3ha
¹³C NMR (101 MHz, CDCl₃)
f1 (ppm)

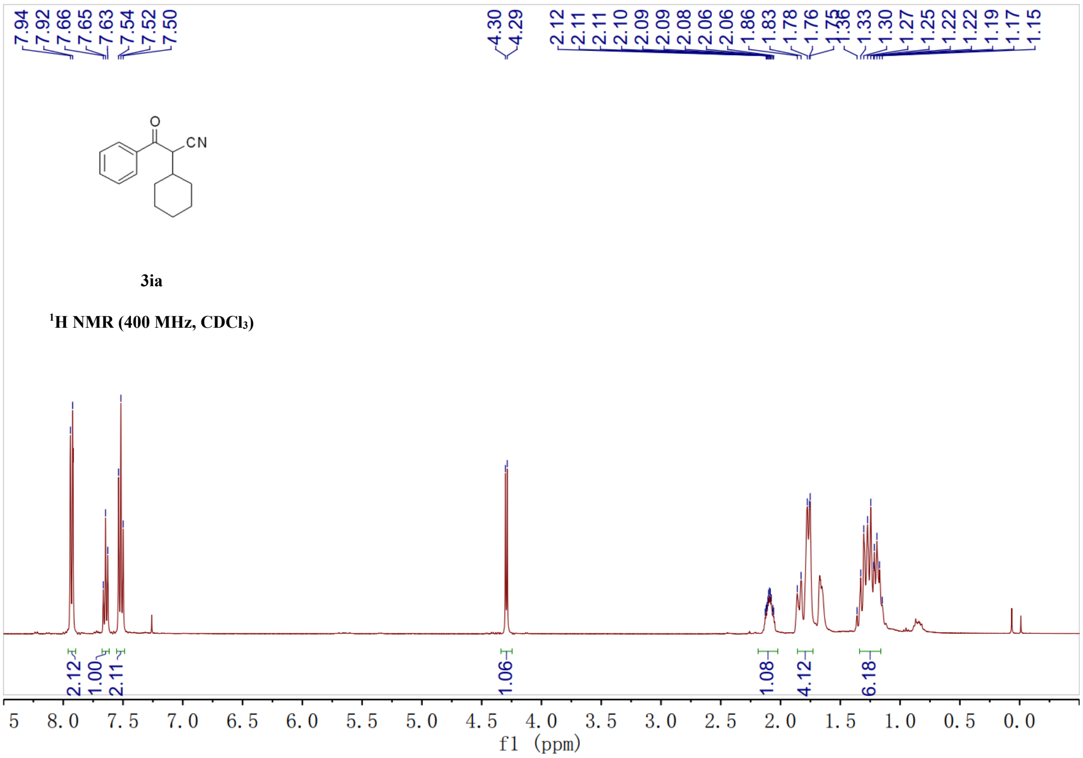
7.94
7.92
7.66
7.65
7.63
7.54
7.52
7.50
4.30
4.29
2.12
2.11
2.11
2.10
2.09
2.09
2.08
2.06
2.06
1.86
1.83
1.78
1.76
1.75
1.36
1.33
1.30
1.27
1.25
1.22
1.22
1.19
1.17
1.15
O
CN
3ia
1H NMR (400 MHz, CDCl3)
2.12
1.00
2.11
1.06
1.08
4.12
6.18
8.0
7.5
7.0
6.5
6.0
5.5
5.0
4.5
4.0
3.5
3.0
2.5
2.0
1.5
1.0
0.5
0.0
f1 (ppm)

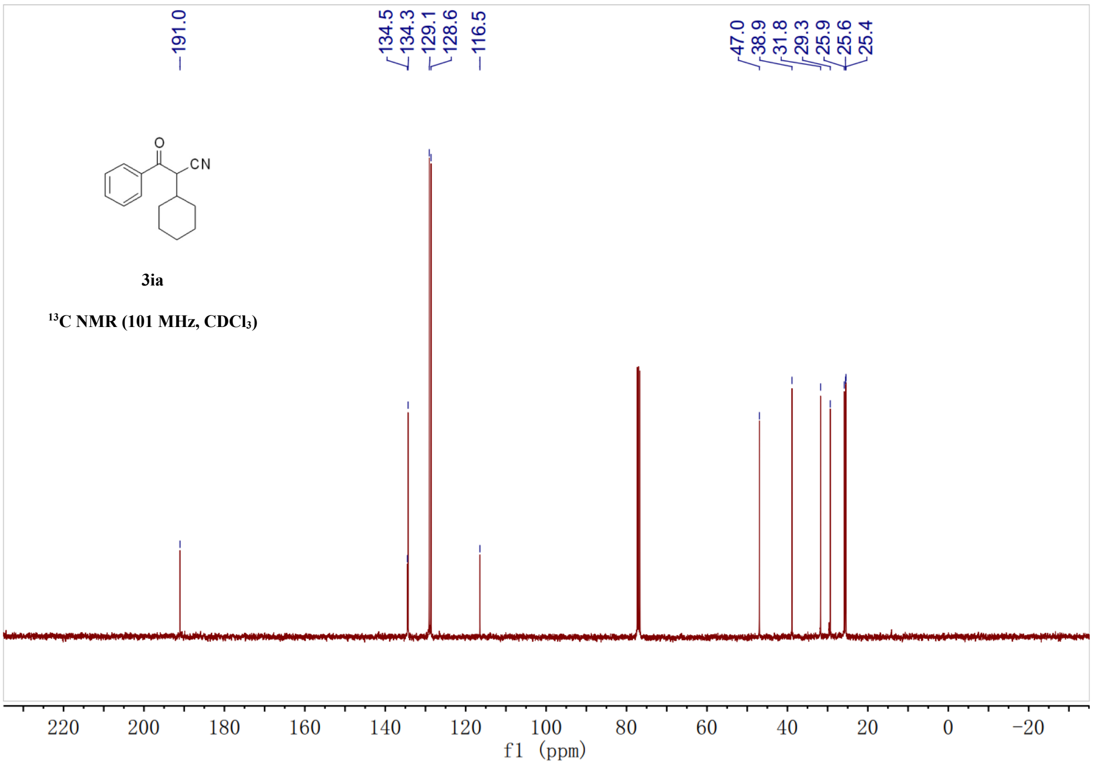
191.0
134.5
134.3
129.1
128.6
116.5
47.0
38.9
31.8
29.3
25.9
25.6
25.4
O
CN
3ia
13C NMR (101 MHz, CDCl3)
220
200
180
160
140
120
100
80
60
40
20
0
−20
f1 (ppm)

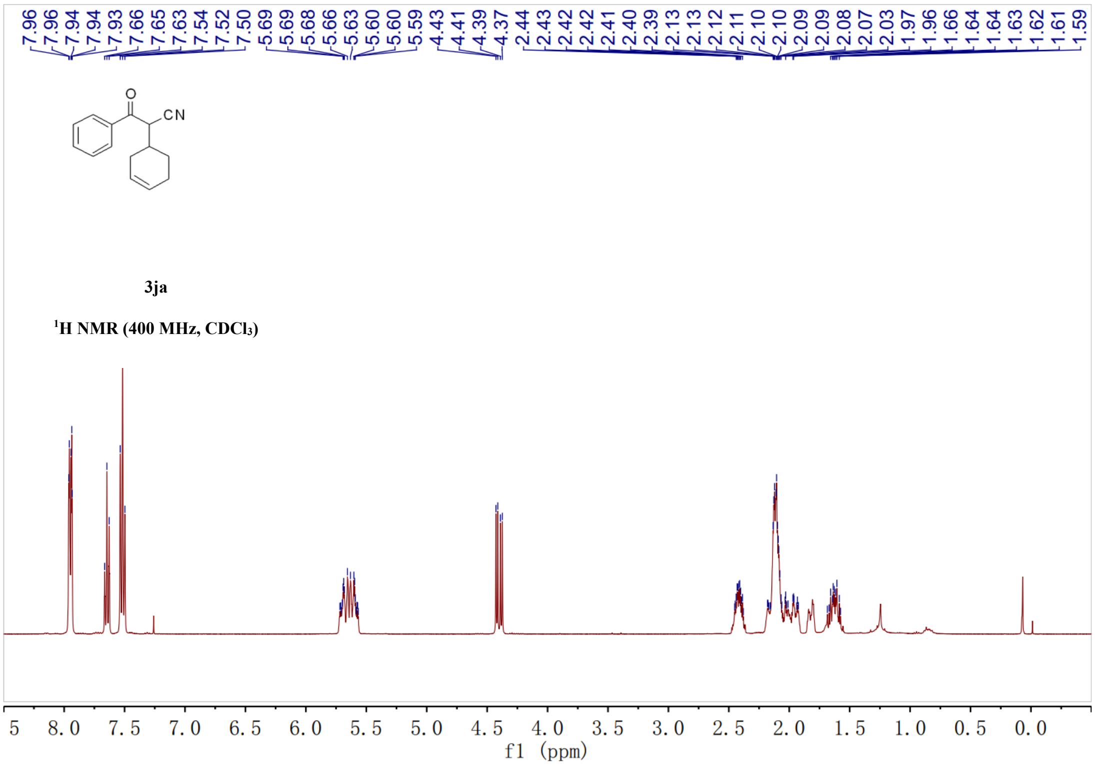
7.96 7.96 7.94 7.94 7.93 7.66 7.65 7.63 7.54 7.52 7.50 5.69 5.69 5.68 5.66 5.63 5.60 5.60 5.59 4.43 4.41 4.39 4.37 2.44 2.43 2.42 2.41 2.40 2.39 2.13 2.13 2.12 2.11 2.10 2.10 2.09 2.09 2.08 2.07 2.03 1.97 1.96 1.66 1.64 1.64 1.63 1.62 1.61 1.59
O
CN
3ja
¹H NMR (400 MHz, CDCl₃)
f1 (ppm)

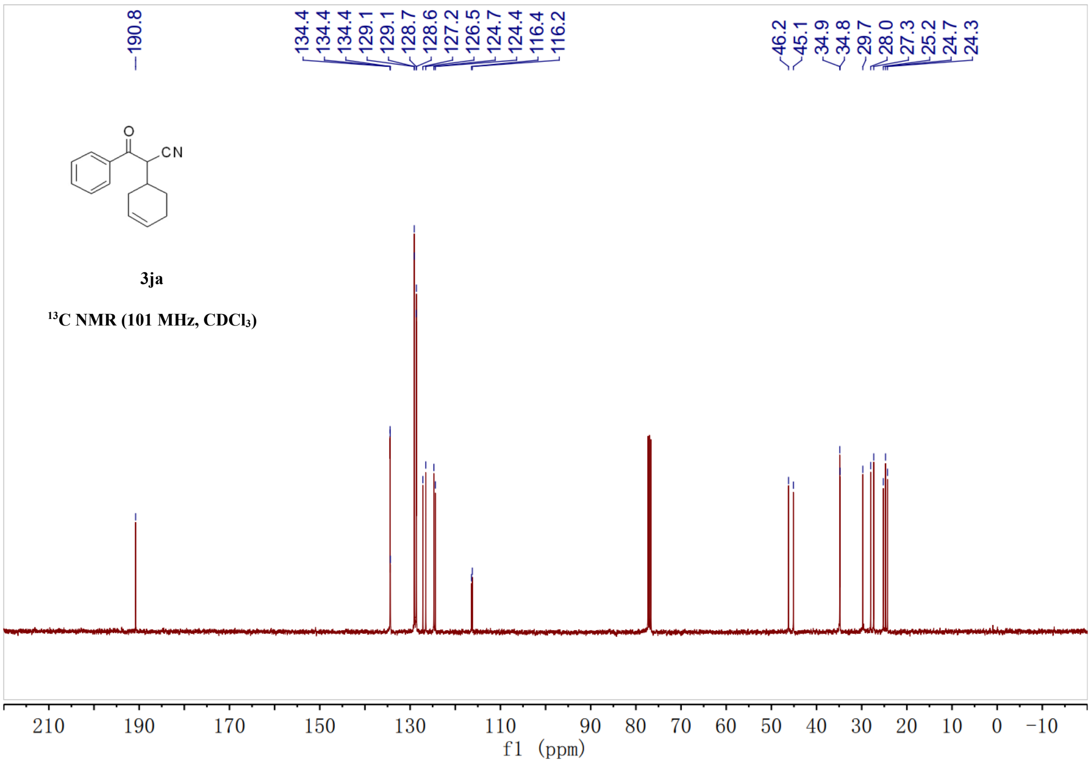
190.8
134.4 134.4 134.4 129.1 129.1 128.7 128.6 127.2 126.5 124.7 124.4 116.4 116.2
46.2 45.1 34.9 34.8 29.7 28.0 27.3 25.2 24.7 24.3
O
CN
3ja
¹³C NMR (101 MHz, CDCl₃)
f1 (ppm)

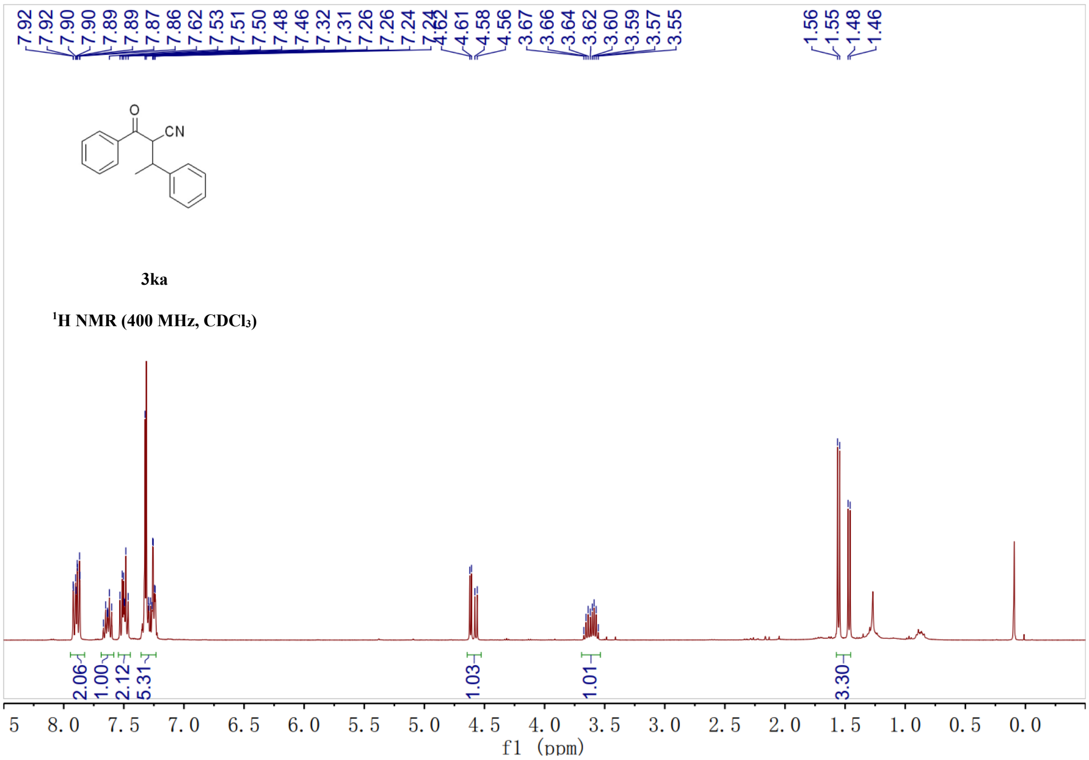
7.92 7.92 7.90 7.90 7.89 7.89 7.87 7.86 7.62 7.53 7.51 7.50 7.48 7.46 7.32 7.31 7.26 7.26 7.24 7.24 4.62 4.61 4.58 4.56 3.67 3.66 3.64 3.62 3.60 3.59 3.57 3.55 1.56 1.55 1.48 1.46
O
CN
3ka
^{1}H NMR (400 MHz, CDCl$_3$)
2.06 1.00 2.12 5.31 1.03 1.01 3.30
8.0 7.5 7.0 6.5 6.0 5.5 5.0 4.5 4.0 3.5 3.0 2.5 2.0 1.5 1.0 0.5 0.0
f1 (ppm)

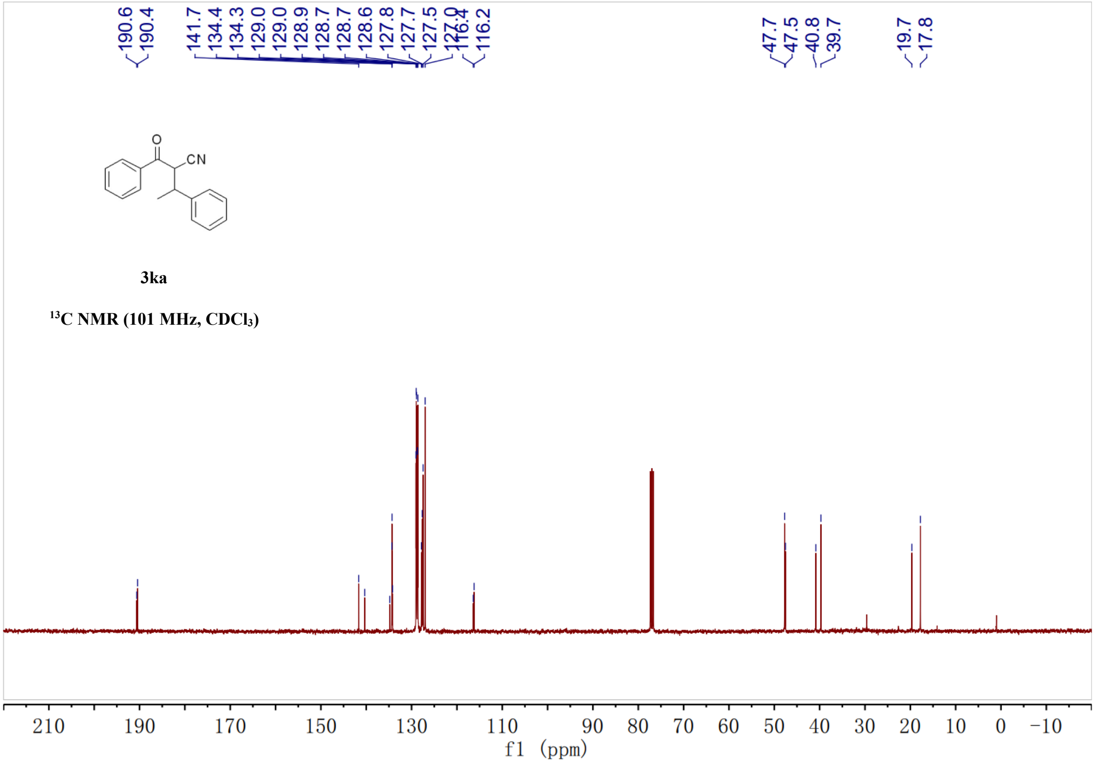
190.6 190.4 141.7 134.4 134.3 129.0 129.0 128.9 128.7 128.7 128.6 127.8 127.7 127.5 127.0 116.4 116.2 47.7 47.5 40.8 39.7 19.7 17.8
O
CN
3ka
^{13}C NMR (101 MHz, CDCl$_3$)
210 190 170 150 130 110 90 80 70 60 50 40 30 20 10 0 −10
f1 (ppm)

7. Copy of ^{1}H NMR Spectra of 4 and 5

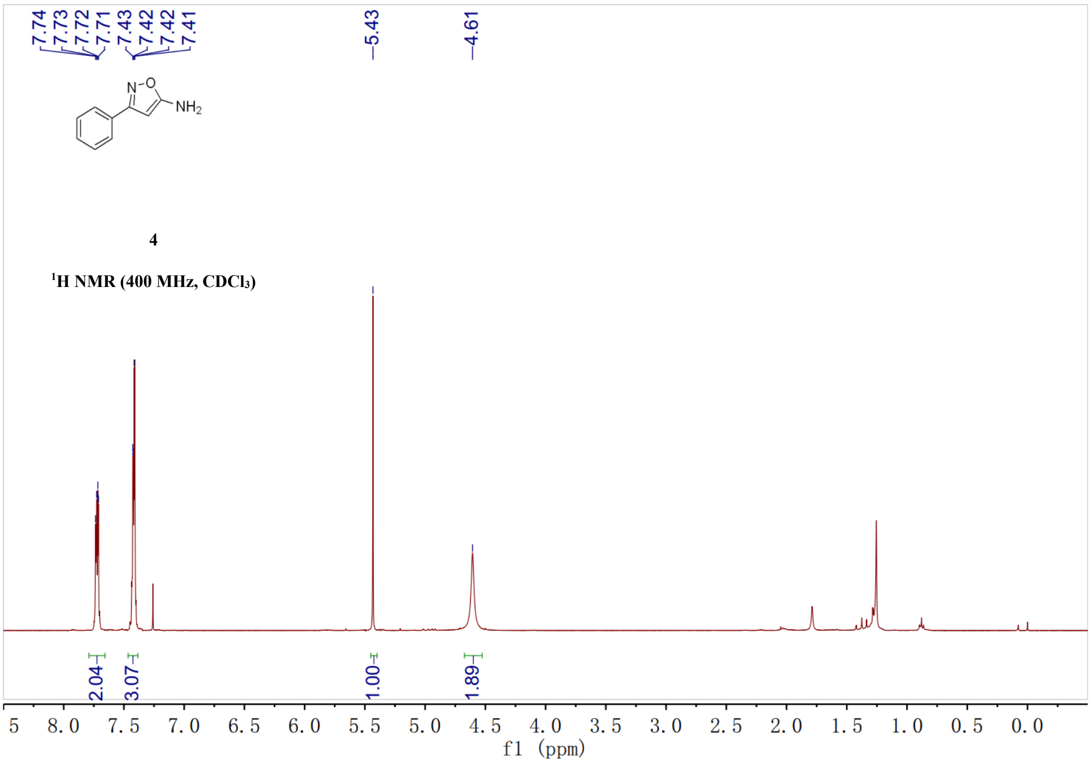

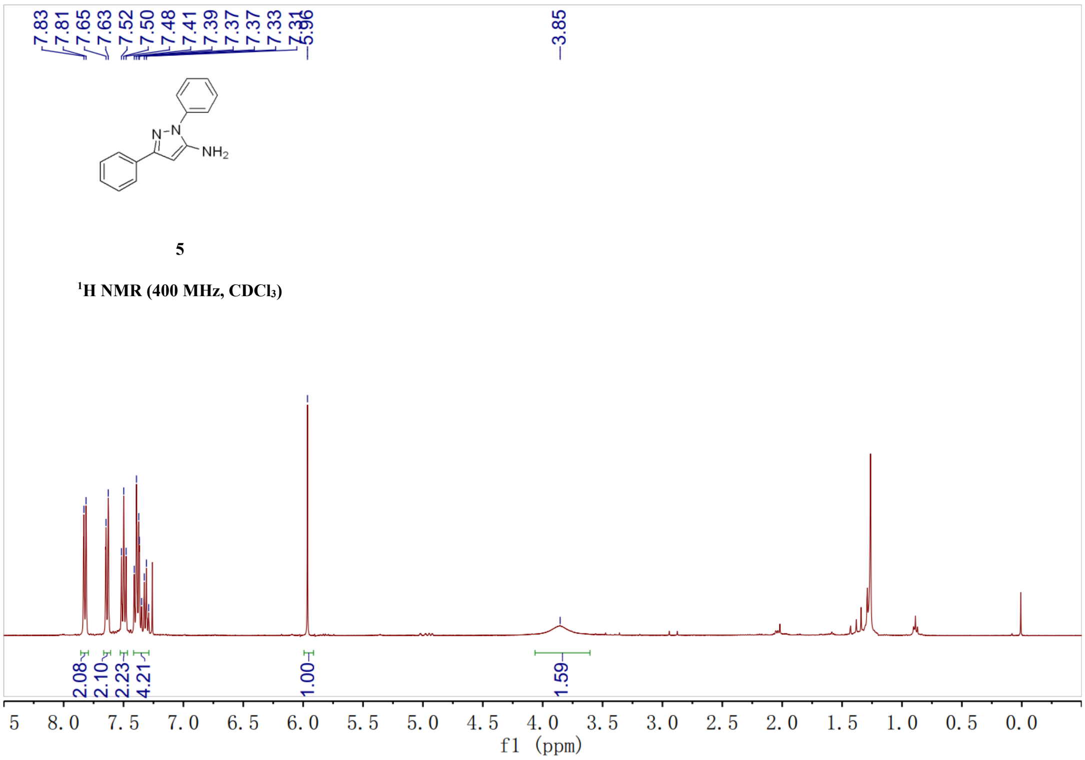

Supplement: Supplementary file 1 [file ol6c01685_si_001.pdf]
